# Altered bone marrow niche forms central innate immune memory driving cardiac dysfunction

**DOI:** 10.1038/s41467-026-76178-z

**Published:** 2026-08-26

**Authors:** Kohsaku Goto, Yukiteru Nakayama, Junichi Sugita, Tsukasa Oshima, Kunihito Kani, Atsushi Kobayashi, Naoto Setoguchi, Yuxiang Liu, Yiyi Yang, Jiaxin Ku, Ryoko Uchida, Eriko Hasumi, Takashi Nagasawa, Issei Komuro, Norihiko Takeda, Ichiro Manabe, Katsuhito Fujiu

**Affiliations:** 1 Department of Cardiovascular Medicine, Graduate School of Medicine, The University of Tokyo, Tokyo, Japan; 2 Department of Advanced Cardiology, Graduate School of Medicine, The University of Tokyo, Tokyo, Japan; 3 Stress-imprinted Immunity Project, Department of Clinical Medical Sciences, Tokyo Metropolitan Institute of Medical Science, Tokyo, Japan; 4 Department of Integrative Physiology, Institute of Science Tokyo, Tokyo, Japan; 5 Laboratory of Stem Cell Biology and Developmental Immunology, Graduate School of Frontier Biosciences, Osaka University, Suita, Japan; 6 International University of Health and Welfare, Tokyo, Japan; 7 Department of Frontier Cardiovascular Science, Graduate School of Medicine, The University of Tokyo, Tokyo, Japan; 8 Department of Systems Medicine, Graduate School of Medicine, Chiba University, Chiba, Japan; 9 Department of Biomedical Engineering, Tohoku University, Miyagi, Japan

**Keywords:** Monocytes and macrophages, Heart failure, Innate immunity, Transplant immunology

## Abstract

We previously showed that heart failure induces innate immune memory in hematopoietic stem and progenitor cells, which contributes to recurrence of heart failure and impaired stress responses in multiple organs. While the bone marrow microenvironment maintains blood cell formation, its role in this memory remains poorly understood. Here we show that bone marrow mesenchymal stromal cells expressing the leptin receptor influence hematopoietic stem and progenitor cells to promote cardiac pathology. Transplanting these stromal cells from mice with heart failure together with healthy hematopoietic cells caused inflammatory macrophages to accumulate in the heart and worsened cardiac remodeling. Mechanistically, heart failure reduced a stromal subpopulation producing heparin-binding epidermal growth factor and suppressed growth factor signaling in hematopoietic cells. Heart failure also activated a fat-forming program in stromal cells. Notably, an equivalent stromal subpopulation exists in human bone marrow, and heart failure was associated with increased bone marrow fat in humans, identifying the bone marrow microenvironment as a regulator of innate immune memory.

## Abstract (web summary)

Heart failure has been shown to induce innate like memory responses in haematopoietic stem/progenitor cells and is linked to immunopathology. Here the authors show the formation of an altered bone marrow niche is linked to innate memory and cardiac dysfunction in heart failure.

## Introduction

The prevalence of heart failure (HF) is rapidly increasing worldwide, partly due to population aging. Despite significant medical advances in recent decades^1^, HF mortality rates remain high, making HF an escalating medical and social challenge^2,3^, and there is an urgent need to develop novel therapeutic and preventive strategies. Chronic inflammation has emerged as a key contributor to the development of cardiovascular diseases (CVD), including HF^4,5^. However, anti-tumor necrosis factor drugs have failed to improve the outcomes of HF patients^6^, and results of more recent clinical trials evaluating other drugs targeting inflammation, including interleukin (IL)−1 blockades^7,8^ and colchicine^9^, have been mixed and conflicting. This highlights the complex regulatory mechanisms underlying inflammation in HF. For instance, recent studies have shown that immune cells, such as macrophages, not only promote cardiac remodeling and HF, they are also crucial to cardiac homeostasis and protection from stress^10^. As such, we need to better understand the mechanisms that regulate cardiovascular inflammation to identify effective therapeutic targets.

Recent studies have shown that innate immune memory or trained immunity in myeloid-lineage cells, characterized by persistent epigenetic and metabolic reprogramming in response to stimuli, plays pivotal roles in the defense against infection and protection from and progression of a variety of diseases, including cancer and CVD^11^. Importantly, innate immune memory can also be established in hematopoietic stem/progenitor cells (HSPCs) in the bone marrow (BM), a phenomenon referred to as central innate immune memory. This central innate immune memory not only promotes myelopoiesis but also modulates the function of their progeny myeloid-lineage cells^12^. While central immune memory can confer greater protection against reinfection^13,14^, it may also promote pathological inflammation. For example, we recently reported that hematopoietic stem cells (HSCs) in the BM can acquire an innate immune memory of cardiac stress, which exacerbates HF and predisposes other organs to pathology^15^. HF induces epigenetic alterations in HSCs, shifting their differentiation trajectory toward myeloid cells and affecting the differentiation of monocytes into macrophages. Progeny monocytes from HF-experienced HSCs more frequently give rise to proinflammatory macrophages rather than mature cardiac tissue-resident macrophages, resulting in a shift in the balance among cardiac macrophage subpopulations that appears to promote cardiac remodeling. A recent study also showed that cerebral infarction induces persistent epigenetic changes in HSPCs, leading to excess accumulation of proinflammatory monocytes/macrophages in the heart and promoting cardiac remodeling and dysfunction^16^. These findings indicate that central innate memory quantitatively and qualitatively alters monocytes/macrophages in the heart, thereby contributing to the development of cardiac dysfunction.

A variety of stimuli and diseases are known to induce central innate immune memory, yet the mechanisms by which HSPCs in BM are educated remain largely unclear. Given that HSPC memory requires stable epigenetic imprinting, it is reasonable to assume that BM niche cells play a critical role in transmitting stress signals to HSPCs. We previously demonstrated that cardiac stress suppresses transforming growth factor-β (TGF-β) signaling in HSCs, and transplantation of bone marrow from HF mice, in which TGF-β signaling in HSCs is inhibited, recapitulates the deterioration of cardiac function. These findings suggest that cardiac stress induces durable epigenomic alterations in HSCs and acts as a carrier of innate immune memory^15^. However, cardiac-stress-induced innate immune memory cannot be fully explained by impaired TGF-β signaling alone, implying that additional niche-derived cues are involved.

BM niche cells, which comprise several cell types, control HSC mobilization, proliferation, and survival^17^. Among them, mesenchymal stromal cells (MSCs) play a particularly important role in HSC homeostasis, in part by expressing key HSC retention factors, such as C-X-C motif chemokine ligand 12 (CXCL12) and stem cell factor (SCF), both of which are critical for preserving HSC stemness^18–21^. Importantly, unbiased mapping of niche–HSC interactions has shown that HSCs physically interact most strongly with MSCs among all niche populations^22^. These findings prompted us to focus on MSCs as potential carriers that transmit cardiac-stress signals to HSCs. Although several markers have been used to identify MSCs in the BM, recent studies have demonstrated that *Lepr*^+^ MSCs are crucial for maintaining HSC homeostasis^23,24^. Their involvement in central innate immune memory of cardiac stress, however, has not been investigated.

In the present study, we hypothesized that *Lepr*^+^ MSCs are involved in the formation of HSPC innate immune memory in HF. We found that HF-experienced MSCs influence HSPCs so that their progeny promote cardiac dysfunction. Mechanistically, HF reduces the MSC subpopulation that produces heparin-binding EGF-like growth factor (HBEGF), leading to alterations in HSPCs characterized by myeloid-biased hematopoiesis and excess accumulation of CCR2^hi^ macrophages in the heart. Our findings demonstrate that *Lepr*^+^ MSCs are an essential component in the education of HSPCs during the establishment of innate immune memory in HF.

## Results

### HF-experienced MSCs affect HSPCs to promote cardiac dysfunction

**Fig. 1:**
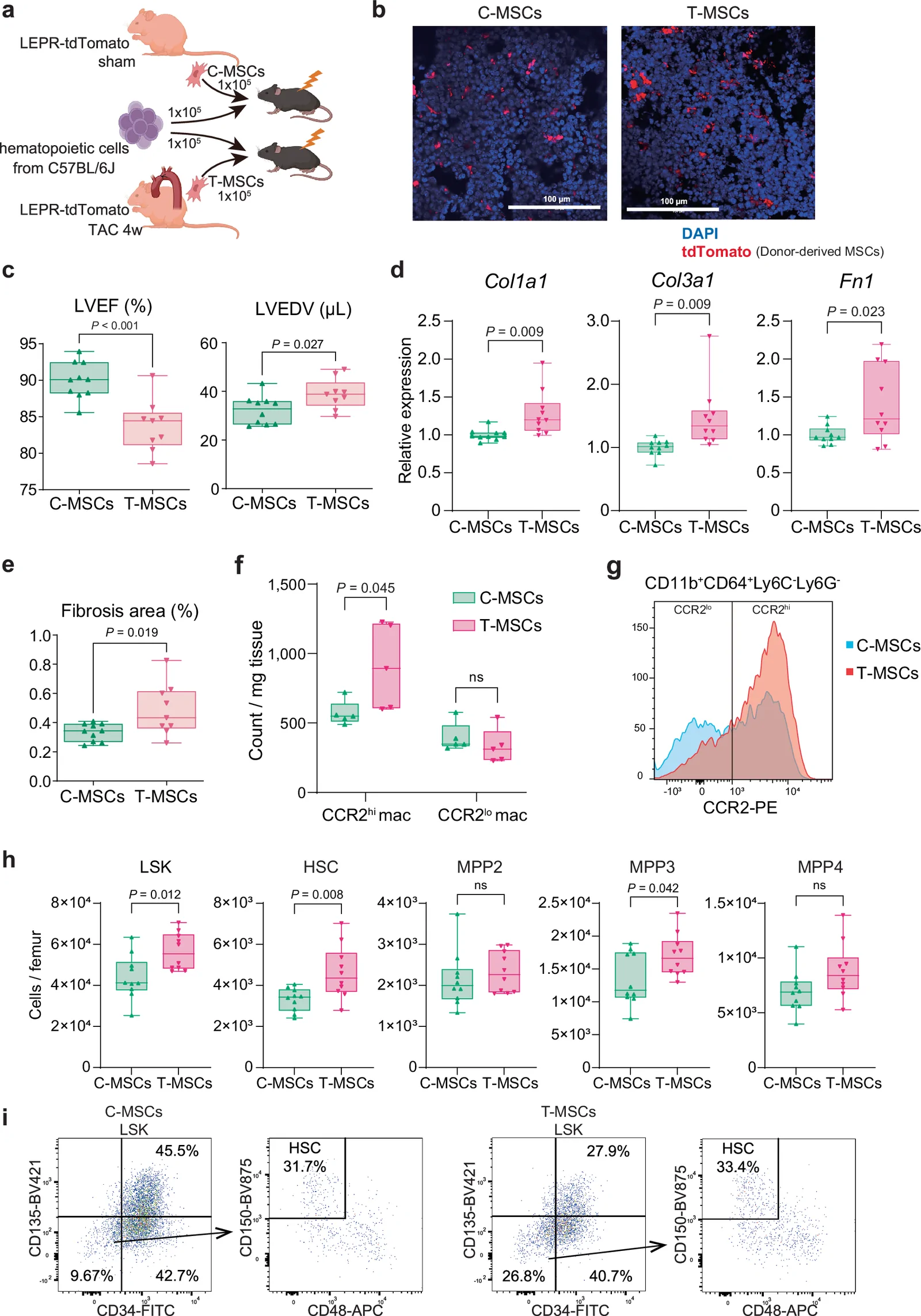
HF-experienced MSCs affect HSCs to promote cardiac dysfunction. **a** Schematic of the co-transplantation strategy of healthy cKit^+^ BM cells enriched with HSPCs and MSCs from post-sham (C-MSCs) or post-TAC (T-MSCs) male mice. **b** Representative fluorescence images of a femur 4 weeks after the co-transplantation. tdTomato signals from donor C-MSCs or T-MSCs are shown in red. **c** Echocardiographic assessment of the left ventricular ejection fraction and left ventricular end-diastolic volume 6 months after the co-transplantation. *n* = 10, 9 male mice for C-MSC and T-MSC groups, respectively. Two-tailed unpaired Student’s *t*-test. Exact *P* value: *P* = 0.0004 (two-tailed unpaired Student’s t-test; t = 4.445, df = 17). **d** Expression of cardiac fibrosis marker genes 3 months after the transplantation. *n* = 10 in each group. Two-tailed unpaired Student’s *t*-test (from two independent experiments). **e** Histological analysis of fibrotic areas identified using Picro-Sirius red staining. The same mice used for the echo studies in c were used. Representative histological images are shown in Supplementary Fig. 3d. Two-tailed unpaired Student’s *t*-test. *n* = 10 mice for the C-MSC group and *n* = 9 mice for the T-MSC group. Data are from three independent experiments with similar results. **f** Numbers of CCR2^hi^ and CCR2^lo^ cardiac macrophages 3 months after the transplantation. *n* = 5 male mice in each group. Two-tailed unpaired Student’s *t*-test. **g** Representative histogram overlays showing CCR2 fluorescence intensity in cardiac macrophages from C-MSC and T-MSC recipient mice. **h** Analysis of HSPC populations in the femur BM 3 months after the transplantation. *n* = 10 in each group. Two-tailed unpaired Student’s *t*-test. **i** Representative flow cytometry plots showing LSK and HSC populations from C-MSC and T-MSC recipient mice. Figure 1a was created in BioRender. F, K. (2026) https://BioRender.com/2v6yj5u.

We previously demonstrated that HF induces innate immune memory in HSPCs^15^, by altering the HSC epigenome, leading HSCs to preferentially differentiate into proinflammatory macrophages that promote cardiac fibrosis and dysfunction^15^. However, the mechanisms underlying the formation of innate immune memory in HSPCs remain incompletely understood. We hypothesized that MSCs might contribute to this process. To test whether HF-experienced MSCs can influence HSPCs so that their progeny induce cardiac dysfunction, we adopted a transplantation strategy in which MSCs from HF mice were co-transplanted with healthy HSPCs (Fig. 1a).

To trace the transplanted MSCs and their progeny, we generated LEPR-tdTomato mice by crossing *Lepr*-*cre* mice that express Cre recombinase from the *Lepr* locus with reporter mice containing a floxed transcriptional stop cassette upstream of tdTomato at the Rosa26 locus. We collected MSCs from LEPR-tdTomato mice 4 weeks after transverse aortic constriction (TAC) to induce HF (T-MSCs), or from control mice after sham operation (C-MSCs), and transplanted them with HSPCs from healthy mice into irradiated mice (Fig. 1a). To avoid confounding effects of sex mismatch and to ensure comparable TAC severity and donor cell loads^25^, MSC transplantation experiments were conducted using male donor cells and male recipients. Prior to MSC isolation, we assessed the establishment of cardiac dysfunction in post-TAC mice. Echocardiography demonstrated systolic dysfunction and left ventricular dilation, and plasma brain natriuretic peptide (BNP) levels were elevated (Supplementary Fig. 1). Four weeks after transplantation, tdTomato expression was detected exclusively in BM across multiple tissues, including heart (Supplementary Fig. 2a). Abundant tdTomato^+^ cells were present in the BM (Fig. 1b, Supplementary Fig. 2b), showing successful engraftment of donor MSCs. Quantification of tdTomato⁺ cells among LepR⁺ MSCs showed that approximately 90% of MSCs were donor-derived in recipient mice, with comparable engraftment efficiency between the two groups (Supplementary Fig. 2c).

Six months after co-transplantation, mice receiving T-MSCs showed decreased cardiac contractile function, chamber dilation (Fig. 1c, Supplementary Fig. 3a), and elevated BNP levels (Supplementary Fig. 3b), although there were no significant changes in the lung wet-to-dry weight ratio (Supplementary Fig. 3c). Cardiac fibrosis was more prominent in the hearts of T-MSC recipients compared to those of C-MSC recipients (Fig. 1d, e, Supplementary Fig. 3d). In the hearts of mice receiving T-MSCs, there was a marked increase in CCR2^hi^ cardiac CD11b^+^CD64^+^Ly6C^low^Ly6G^-^ macrophages, while the number of CCR2^lo^ cardiac macrophages remained unchanged (Fig. 1f, g, Supplementary Fig. 4). In the BM of the T-MSC recipients 3 months after transplantation, the numbers of long-term HSCs (LT-HSCs) and myeloid-biased MPP3 cells^26^ were higher than in recipients of C-MSCs (Fig. 1h, i, Supplementary Fig. 5). In contrast, HSPC populations were comparable between the two groups at early time points after transplantation^27^ (Supplementary Fig. 6), indicating that initial homing and engraftment of HSCs and early hematopoietic reconstitution were similar, and that the effects of T-MSC on HSPCs at 3 months were independent of initial engraftment efficiency. Collectively, these results showed that co-transplantation of HF-experienced MSCs along with healthy HSPCs promoted cardiac remodeling and functional impairment, which were accompanied by excess accumulation of CCR2^hi^ proinflammatory macrophages^28–32^. The results also suggested that T-MSC co-transplantation induced myeloid-biased hematopoiesis. These findings support the idea that MSCs educate HSPCs.

### TAC suppresses MSC HBEGF-to-HSC signaling

Having found that HF-experienced *Lepr*^+^ MSCs alter HSPCs and promote cardiac dysfunction, we next asked how MSCs affected HSPCs. To analyze alterations in MSCs and HSPCs and their communications after TAC, we performed single-cell RNA-sequencing (scRNA-seq) with *Lepr*^+^ MSCs and Lin^-^Sca1^+^cKit^+^ (LSK) cells, which were enriched in HSPCs, obtained from control (sham) and post-TAC mice 4 weeks after their procedures. Using hashtag antibodies^33^, we analyzed control and post-TAC cells from 3 and 8 male mice, respectively.

**Fig. 2:**
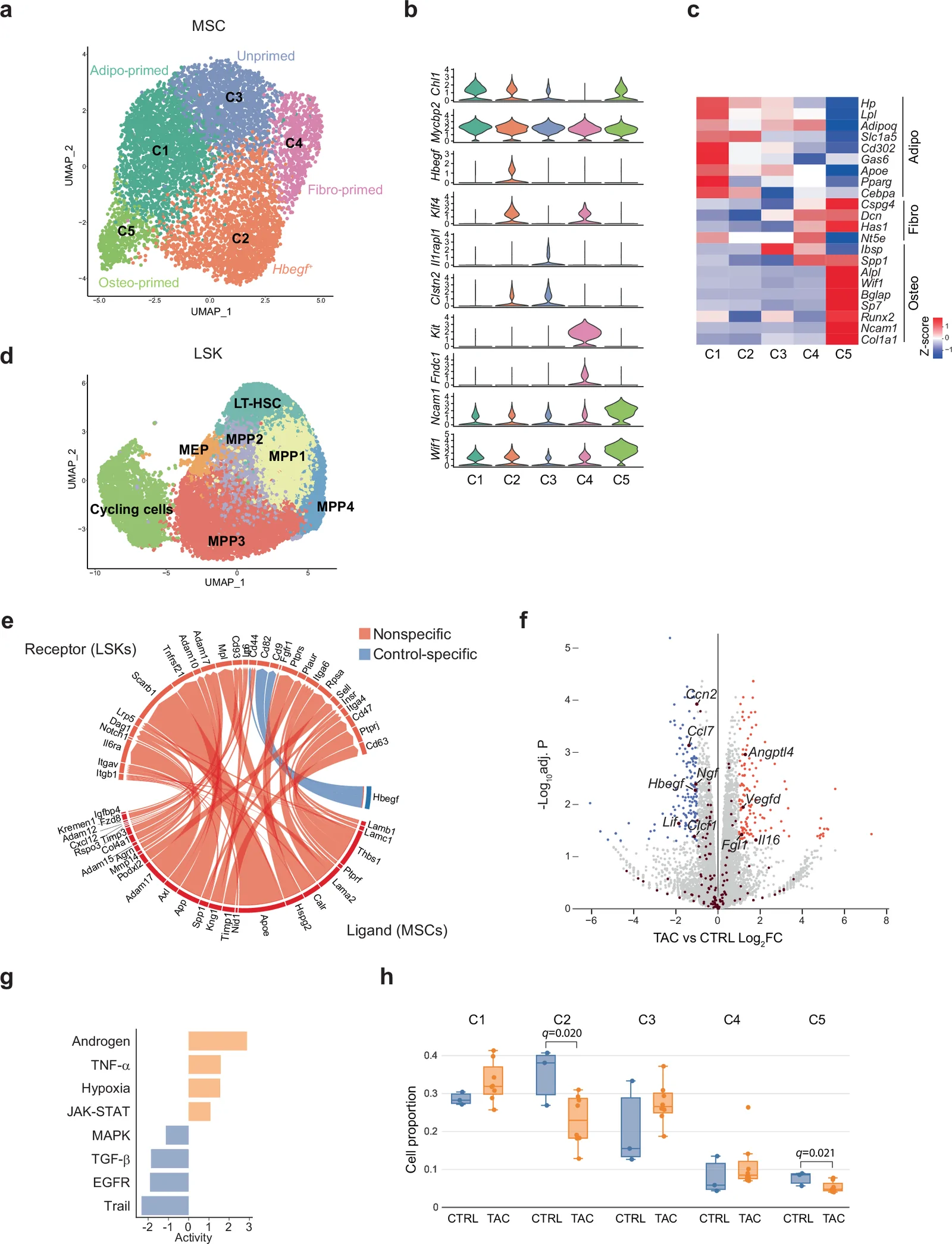
TAC suppresses MSC HBEGF-to-HSC signaling. **a** UMAP plot of *Lepr*^+^ MSCs from male mice (*n* = 3 control; *n* = 8 TAC) 4 weeks after surgery. **b** Marker gene expression in each cluster. Additional marker genes are shown in Supplementary Fig. 7. **c** Heatmap showing expression of known MSC subpopulation markers. **d** UMAP plot of LSKs from the same male mice used for MSC scRNA-seq. **e** NicheNet analysis of inferred ligand-receptor interactions between MSCs (ligand producing) and LSKs (receptor-expressing). **f** Differential gene expression analysis of MSCs. Upregulated and downregulated genes (FDR < 0.05) are shown in red and blue, respectively. Cytokine and growth factor genes are highlighted in dark red, and those with FDR < 0.05 are labeled. Differential expression was assessed using edgeR with two-sided tests, and *P* values were adjusted for multiple comparisons using the Benjamini–Hochberg method (FDR < 0.05). **g** PROGENy pathway enrichment analysis. Positive pathway scores indicate increased pathway activity in post-TAC HSCs compared with controls. Top 8 pathways are shown. **h** Proportions of cells within each MSC cluster in control (CTRL) and TAC groups. Differences in proportions were analyzed using beta regression, and *P* values were adjusted using the Benjamini–Hochberg method. Adjusted *P* values are shown only for significantly different comparisons. *n* = 3 for control male mice, *n* = 8 for TAC male mice.

Clustering of *Lepr*^+^ MSCs identified five subpopulations characterized by distinctive gene expression patterns (Fig. 2a, b, Supplementary Fig. 7a, b). Expression of previously identified MSC subpopulation markers in mouse and human datasets (Fig. 2c)^24,34^ indicated that cluster C1 cells, which were characterized by strong expression of *Chl1*, were primed to differentiate into adipocytes (adipo-primed). *Kit*^hi^ C4 cells expressed fibrogenic MSC markers (fibro-primed). *Ncam1*^hi^ C5 cells expressed osteogenic markers (osteo-primed). The lack of strong expression of known marker genes and the results of RNA velocity analysis suggested that C3 cells were baseline unprimed cells (Supplementary Fig. 7c). C2 cells were characterized by higher level expression of *Klf4*, *Id2* and immediate early response genes, such as *Jun* and *Fos* (Fig. 2b, Supplementary Fig. 7a, b), but they did not show expression of markers known to conform to the previously identified MSC subpopulations. Notably, they also expressed *Hbegf*, which encodes HBEGF. Importantly, the same five clusters, including their characteristic marker gene signatures, were also observed in MSCs isolated from female mice (Supplementary Fig. 8), indicating that the MSC subpopulation structure is conserved between sexes.

LSK cells were clustered into seven subpopulations based on the reported expression patterns of marker genes^26,35,36^. These included long-term HSCs (LT-HSCs), four multipotent progenitor subsets (MPP1-4), megakaryocyte/erythrocyte progenitor cells (MEP), and cycling cells (Fig. 2d, Supplementary Fig. 9). MPP1 shares characteristics with short-term HSCs (ST-HSCs). MPP2 and MPP3 are myeloid-biased progenitors, whereas MPP4 is a lymphoid-skewed progenitor population.

To detect intercellular interactions between MSCs and LSK cells that mediate alterations in HSPCs in HF, we used NicheNet^37^ to analyze the ligand-receptor pathways related to the gene expression changes induced in LSK cells　(LT-HSC and MPP1-4). Among the ligands produced by MSCs that might contribute to the differential gene expression in LSK cells, *Hbegf* signaling emerged as selectively diminished in post-TAC conditions (Fig. 2e). In addition, pseudobulk differential expression analysis of MSCs using edgeR^38^ showed that *Hbegf* was among the ten cytokine/growth factor genes that were differentially expressed and downregulated by TAC MSCs (Fig. 2f). Moreover, pathway analysis of HSCs (LT-HSC and MPP1) showed that in addition to TGF-β signaling, which we previously showed to be downregulated in an independent dataset^15^, EGFR signaling was downregulated in post-TAC HSCs (Fig. 2g). Together, these findings strongly suggested that HBEGF signaling from MSCs was suppressed after TAC.

While very little is known about the function of HBEGF in the BM, previous studies have shown that EGFR signaling promotes HSPC proliferation after irradiation, and its inhibition facilitates HSC mobilization induced by G-CSF^39,40^. Chang et al. recently showed that hematopoietic inhibition of EGFR signaling promotes myeloid skewing in mice and that EGF treatment activates the genes related to self-renewal in aged HSCs^41^. Notably, *Hbegf* expression was enriched in *Klf4*^+^ C2 MSCs, which was designated as the *Hbegf*^+^ cluster (Fig. 2b, Supplementary Fig. 8b). The proportion of MSCs belonging to the *Hbegf*^+^ cluster was significantly decreased after TAC, which suggested *Hbegf* downregulation in MSCs was attributable, at least in part, to the smaller *Hbegf*^+^ subpopulation. This reduction was observed in both sexes (males, Fig. 2h; females, Supplementary Fig. 8c).

### *Hbegf* knockdown in MSCs promotes myeloid skewing and cardiac dysfunction

**Fig. 3:**
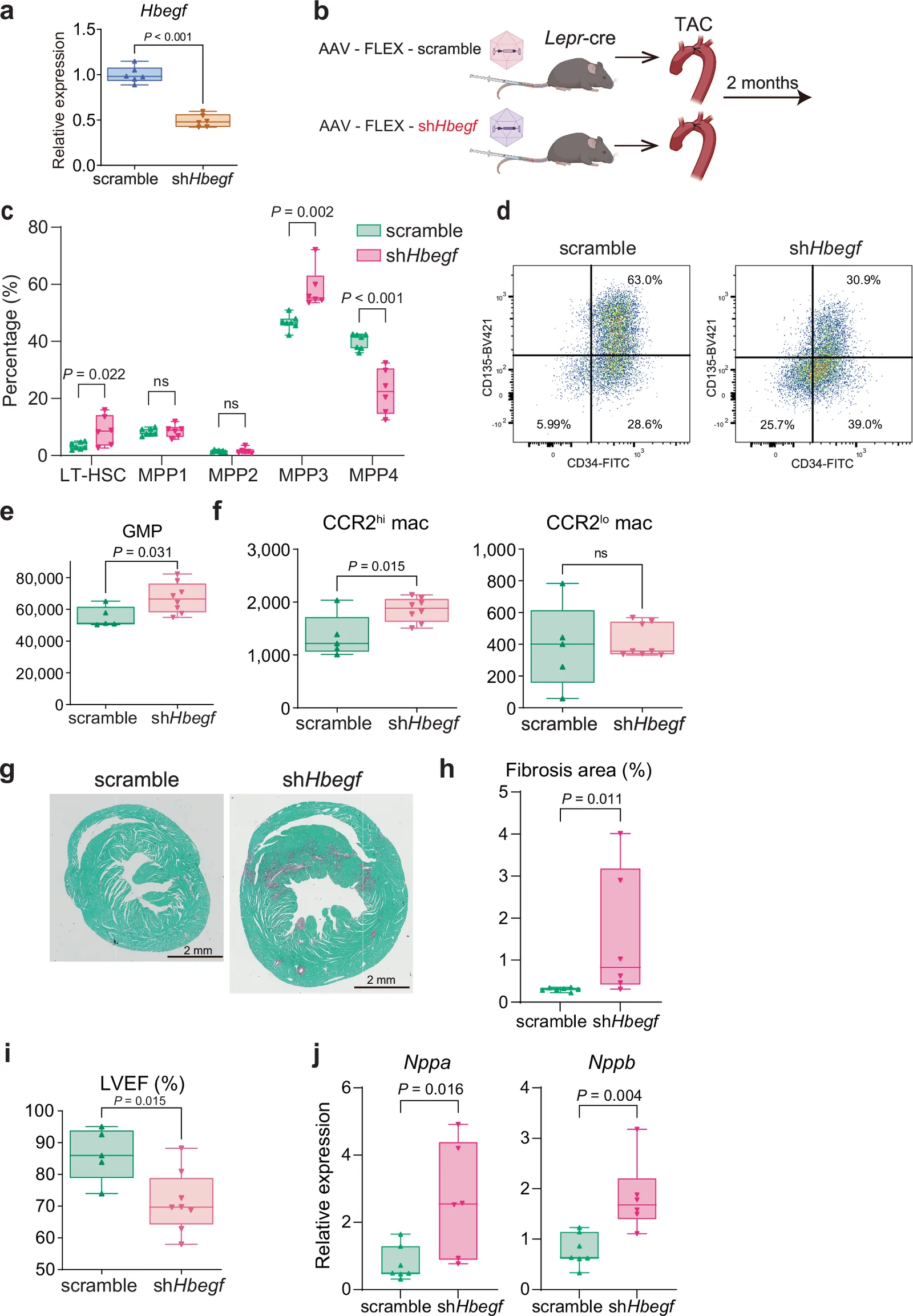
Hbegf downregulation in MSCs promotes myeloid skewing and cardiac dysfunction. **a** Expression of *Hbegf* in lentivirally generated NIH-3T3 stable cell lines overexpressing murine *Hbegf* and Cre recombinase following transfection with the AAV-FLEX–sh*Hbegf* transgene plasmid. *n* = 6 per group. Exact *P* value: *P* < 0.0001 (two-tailed unpaired Student’s t-test; t = 10.95, df = 10). **b** Schematic of the experimental workflow. *Lepr*-Cre male mice received intravenous AAV administration followed by TAC. **c** Fractions of primitive HSPC subpopulations. Exact *P* values: LT-HSC, *P* = 0.0220; MPP1, *P* = 0.8770; MPP2, *P* = 0.7289; MPP3, *P* = 0.0024; MPP4, *P* = 0.0006 (two-tailed unpaired Student’s t-tests after logit transformation; *n* = 7 scramble, *n* = 6 shHbegf). **d** Representative flow cytometry plots showing LSK populations. **e** Numbers of granulocyte/monocyte progenitor (GMP) per femur. **f** Numbers of CCR2^hi^ and CCR2^lo^ cardiac macrophages normalized to heart weight (cells per milligram of heart tissue). **g** Representative cardiac sections stained with Picrosirius Red/Fast Green; collagen fibers appear red, and the non-collagen components green. **h** Fibrosis area in cardiac tissue. **i** Left ventricular ejection fractions measured by echocardiography. **j** Ventricular *Nppa* and *Nppb* mRNA expression. Data for Fig. 3c, d, g, h, j were obtained from the same experiment (*n* = 7 scramble, *n* = 6 sh*Hbegf*). Data for Fig. 3e, f, i were obtained from independent experiments (*n* = 5 scramble, *n* = 8 sh*Hbegf*). Two-tailed unpaired Student’s *t*-tests were used for Fig. 3a, e, f, i, j and two-tailed unpaired Student’s *t*-tests after logit transformation were used for Fig. 3c, h. Figure 3b was created in BioRender. F, K. (2026) https://BioRender.com/4ux5x8g.

We then addressed whether reducing HBEGF in MSCs affects hematopoiesis and, in turn, alters the cardiac response to TAC. To selectively knock down *Hbegf* in MSCs, we generated an adeno-associated virus (AAV) vector carrying a FLEX (flip-excision) cassette encoding either scramble or anti-*Hbegf* short hairpin RNA (shRNA) expressed in a Cre-dependent manner (Supplementary Fig. 10a). We confirmed efficient knockdown of *Hbegf* by AAV-FLEX–sh*Hbegf* in Cre-expressing cells in vitro (Fig. 3a). In addition, knockdown of *Hbegf* in cultured MSCs did not alter the expression of representative MSC lineage or subcluster marker genes (Supplementary Fig. 10b).

We administered either AAV-FLEX-scramble or AAV-FLEX-sh*Hbegf* to male *Lepr*-Cre mice, and two weeks later, we performed TAC to induce cardiac pathology (Fig. 3b). Two months after TAC, mice treated with AAV-FLEX-sh*Hbegf* displayed a larger LT-HSC compartment and an expanded myeloid-primed MPP3 fraction, accompanied by a reduced lymphoid-primed MPP4 fraction in the bone marrow compared with AAV-FLEX-scramble controls, indicating a shift toward myeloid lineage commitment (Fig. 3c, Supplementary Fig. 11a). A similar trend was observed in downstream myeloid progenitor populations, whereas circulating myeloid cells and splenic monocytes remained unchanged (Fig. 3e, Supplementary Fig. 11b–g). In the heart, AAV-FLEX-sh*Hbegf* administration increased CCR2^hi^ macrophages, but not CCR2^lo^ macrophages (Fig. 3f). Ly6C^hi^ monocyte/macrophage and neutrophil abundance in the heart was not altered (Supplementary Fig. 11h). Mice receiving AAV-FLEX-sh*Hbegf* exhibited exacerbated cardiac remodeling after TAC, characterized by increased myocardial fibrosis (Fig. 3g, h) and impaired cardiac function (Fig. 3i, Supplementary Fig. 12a). Expression of the cardiac stress markers *Nppa* and *Nppb* was also significantly elevated (Fig. 3j), although the lung wet-to-dry weight ratio was comparable between the two groups (Supplementary Fig. 12b), consistent with the known resistance of C57BL/6 mice to pulmonary congestion^42,43^.

We then addressed whether the lack of *Hbegf* in MSCs might affect HSPC fate by co-transplanting MSCs isolated from male *Lepr*-Cre mice transduced with either AAV-FLEX-sh*Hbegf* or AAV-FLEX-scramble with WT LSKs into WT mice. Circulating lymphocytes were reduced in recipients of AAV-FLEX-sh*Hbegf-*transduced MSCs compared with those of control MSCs (Supplementary Fig. 13), similarly to findings in the mice that received AAV-FLEX-sh*Hbegf* in vivo (Fig. 3c, Supplementary Fig. 11). Collectively, these results showed that knockdown of *Hbegf* in MSCs reproduced the myeloid-skewed phenotype, supporting the notion that repression of *Hbegf* in MSCs is sufficient to drive the myeloid shift.

To directly assess the effects of HBEGF on HSC fate, we performed colony-forming unit (CFU) assays in vitro. We found that recombinant HBEGF increased multilineage CFU–granulocyte/erythroid/megakaryocyte/monocyte (CFU-GEMM) colonies and reduced CFU–granulocyte/monocyte (CFU-GM) in a dose-dependent manner (Supplementary Fig. 14). This pattern indicates that HBEGF enhances the multilineage differentiation potential while reducing myeloid-skewed output.

Together, these results show that *Hbegf* suppression in MSCs induces myeloid skewing and aggravates cardiac dysfunction following TAC, suggesting that MSC-derived HBEGF acts as a key regulator of hematopoietic and immune responses during cardiac stress.

### TAC activates adipogenic programs in MSCs

**Fig. 4:**
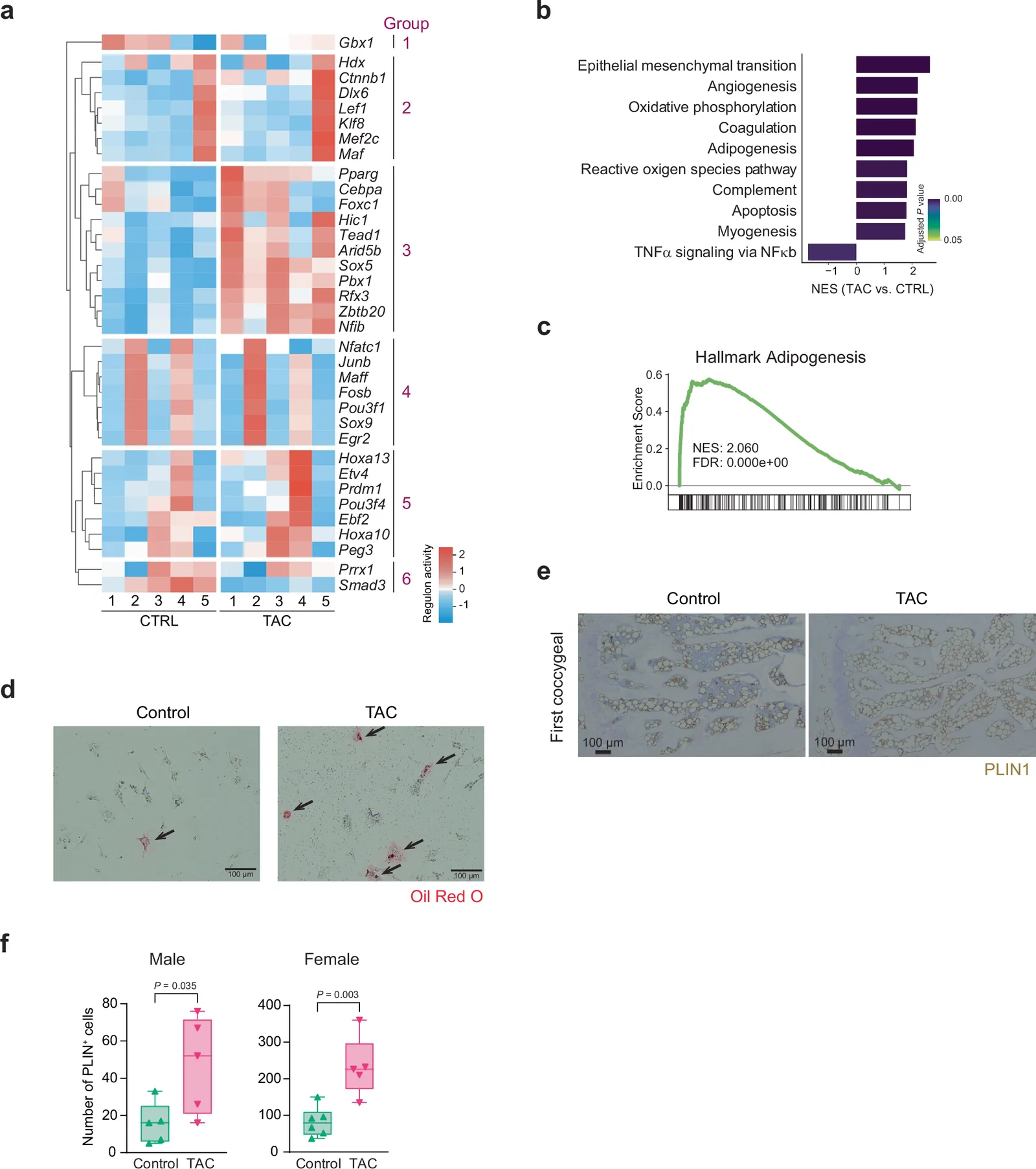
Activation of the adipogenic program in MSCs. **a** Heatmap showing the scaled average regulon activity scores of the top 5 regulons specific to each cluster in the control and post-TAC datasets. Rows represent the TFs that control the regulons, which are hierarchically clustered. Based on the dendrogram, regulons were divided into six groups exhibiting distinct activity patterns. **b** Top 10 hallmark gene sets enriched in TAC versus control MSCs, ranked based on normalized enrichment score (NES). LEPR^+^ MSCs were isolated from male mice 4 weeks after either sham or TAC operation. *n* = 2 for sham, *n* = 3 for TAC. Gene sets were ranked by normalized enrichment score, and statistical significance was determined by gene set enrichment analysis (two-sided) with Benjamini–Hochberg-adjusted *P* values. **c** GSEA enrichment plot of the adipogenesis Hallmark gene set. **d** Oil red O staining of MSC cultures derived from bone marrow cells of control and TAC 4 week mice cultured in the adipogenic medium for 7 days. *n* = 6 mice per group. Data are from three independent experiments with similar results. **e** Representative histological sections of the first coccygeal segment from control and post-TAC mice. 3,3’-diaminobenzidine (DAB) staining was used to stain PLIN1. *n* = 6 mice per group. Data are from three independent experiments with similar results. **f** Numbers of adipocytes in the distal femur analyzed with immunohistochemical imaging of PLIN1 in male and female mice. *n* = 5 for each male group; *n* = 6 for control, *n* = 5 for TAC in females. Two-tailed unpaired Student’s *t*-test.

Our results so far demonstrated that TAC specifically impaired the inferred interaction between MSCs and HSCs via HBEGF–EGFR signaling and reduced the *Hbegf*-expressing MSC population in BM. Furthermore, MSC-specific knockdown of *Hbegf* promoted myeloid-skewed differentiation in the hematopoietic lineage, similar to that observed after TAC, and enhanced myocardial remodeling. Collectively, these findings strongly suggest that changes in MSC populations after TAC play a pivotal role in modulating HSCs. Given the importance of these MSC changes, we further investigated how TAC alters MSCs by analyzing transcription factor (TF) regulons using SCENIC^44^. The activities of the top regulons specific to each cluster were hierarchically grouped across clusters and conditions (Fig. 4a). We also analyzed the activities of TFs previously shown to be involved in MSC differentiation (Supplementary Fig. 15). In the control dataset, C1 cells were characterized by high activities of *Pparg* and *Cebpa*, the adipogenic master TFs, which is consistent with the high adipocyte-associated marker gene expression. As C2 cells express higher levels of *Klf4*, its regulon activity was high in those cells (Supplementary Fig. 15). C2 cells were also characterized by higher activities of *Sox9*, *Nfatc1*, and *Pou3f1*, as well as AP1 (*Junb* and *Fosb*) and two other immediate early response TFs, *Maff* and *Egr2*. In C3 cells, the activities of *Hoxa10* and *Peg3*, which are involved in the maintenance of stemness and proliferation of several stem cell types^45^, as well as *Prrx1*, a marker of primitive mesenchymal cells^46^. The regulon activities in C3 cells from the control mice may correspond to their unprimed states, as determined from their gene expression and RNA velocity (Supplementary Fig. 7c). C4 cells exhibited high levels of activity of some of the same regulons as C2 cells. They also exhibited a high *Twist1* activity, which is involved in fibroblast activation^47^, as well as *Smad3* and *Prrx1*, the latter of which is reportedly important for myofibroblast differentiation in response to TGF-β^46^. C5 cells were characterized by high activities of TFs involved in osteogenesis, such as *Mef2c* and *Sp7* (osterix).

After TAC, the activities of the group 3 regulons were markedly upregulated widely in all clusters (Fig. 4a). Group 3 regulons include *Pparg*, *Cebpa*, and *Foxc1*, which regulate adipocyte differentiation. The regulons of other known TFs involved in adipocyte differentiation, including *Cebpb*, *Srebf*, *Klf15*, and *Foxo1*, were also upregulated after TAC (Supplementary Fig. 15), suggesting *Lepr*^+^ MSC-wide activation of adipogenic programs. While regulons specific to other MSC subpopulations (e.g., groups 4 and 5) were also activated, their activation was mostly within their respective subpopulations, suggesting cell type-specific responses (Fig. 4a). Consistent with that idea, the activities of osteogenic TFs were mainly activated only in C5 cells (Supplementary Fig. 15).

We also used bulk RNA-seq to analyze transcriptomic changes in MSCs from male mice that underwent TAC (Fig. 4b, c). Gene set enrichment analysis (GSEA) showed that upregulation of MSigDB Hallmark gene sets^48^, including those involved in epithelial-mesenchymal transition, angiogenesis, oxidative phosphorylation, coagulation, and adipogenesis in post-TAC MSCs (Fig. 4b, c). The significant enrichment of the adipogenesis gene set in TAC MSCs is consistent with the upregulation of adipogenesis-related regulons in scRNA-seq datasets.

Given the data suggesting widespread activation of the adipogenic program in MSCs, we assessed their adipogenic potential. In in vitro adipogenic differentiation assays^49,50^, we observed higher numbers of oil red O-positive adipocytes among post-TAC BM-derived MSCs than control BM-derived MSCs (Fig. 4d, Supplementary Fig. 16a, b). In addition, expressions of adipocyte marker genes, including *Fabp4*, *Adipoq*, *Pparg*, and *Plin1*, were upregulated in post-TAC BM-derived MSCs (Supplementary Fig. 16c). These results demonstrated that TAC enhanced adipogenic differentiation potential in MSCs.

We then addressed whether adipocyte numbers were increased in BM after TAC. Immunochemical staining for an adipocyte marker, PLIN1, revealed higher numbers of adipocytes in BM samples from TAC mice than from control mice, including BM from the first coccygeal segments and the femurs (Fig. 4e, Supplementary Fig. 17). Increased adipocytes in the femur after TAC was observed in both male and female mice (Fig. 4f).

To further assess the regulatory mechanism of MSCs that responds to TAC, we analyzed the TF networks that control *Hbegf* using the regulons inferred by SCENIC. Network analysis identified KLF4, and immediate early response TFs Jun and EGR1/2 as hubs situated directly upstream of *Hbegf* (Supplementary Fig. 18a). Because TAC activates adipogenic PPARγ activity, we analyzed the networks linking *Hbegf* and *Pparg*. This analysis identified Hes1 as a hub TF in addition to KLF4, C/EBPβ, and immediate early response TFs (Supplementary Fig. 19a). Hes1 was inferred to control TFs regulating adipocyte differentiation, including *Klf15*, *Yy1*, and *Cebpb*. Hes1 is an essential effector of the Notch signaling pathway^51^, and inhibition of Notch signaling has been shown to promote adipocyte differentiation in BM MSCs^52^. After TAC, *Hes1* expression was reduced across MSC subpopulations (Supplementary Fig. 19b), and GSEA showed downregulation of Notch pathway gene sets in MSCs (Supplementary Fig. 19c). In silico perturbation analysis using Dynamo^53^ showed that *Hes1* knockdown redirected predicted differentiation trajectories away from *Hbegf*^+^ C2 cluster toward adipogenic C1 cluster (Supplementary Fig. 19d). Collectively, our analyses suggest that a transcription factor network consisting of PPARγ, KLF4, and Hes1 controls MSC fate after TAC, and that reduced Notch signaling may skew MSC populations toward adipocytes.

### Human LEPR^+^ MSC subpopulation expresses *HBEGF*

**Fig. 5:**
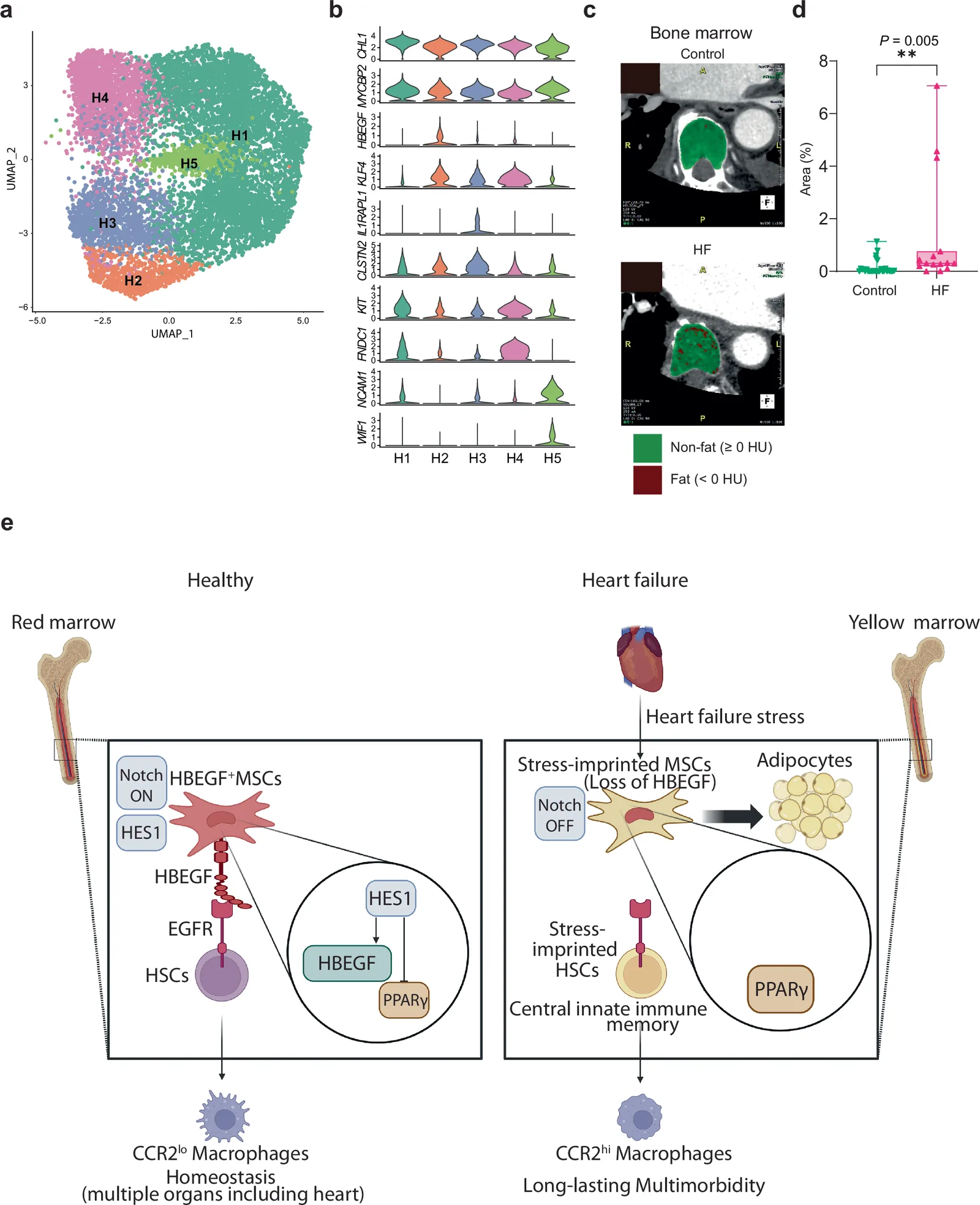
Human HBEGF^+^ MSCs and BM adiposity in HF. **a** Clustering of human *LEPR*^+^ MSCs. **b** Expression of the marker genes in human *LEPR*^+^ MSCs. Violin plot of the full marker genes corresponding to Supplementary Fig. 7b is shown in Supplementary Fig. 20a. **c** Representative axial CT images of the 7th thoracic vertebra (T7) from a healthy control subject (left) and a patient with HF (right). Bone marrow regions with CT attenuation below 0 HU, corresponding to fat, are shown in red, and regions with attenuation at or above 0 HU are shown in green. **d** Percentage areas of CT values below 0 within the range from −200 to 1000 in CT values on coronary CT images of the thoracic vertebral BM in HF patients and age- and sex-matched healthy controls. Two-tailed unpaired Student’s t-test. *n* = 30 for control, *n* = 15 for heart failure. Box-and-whisker plots show the median, the 25th and 75th percentiles (box bounds), and the minimum and maximum values (whiskers). **e** Schematic model summarizing the proposed mechanism, comparing the healthy state (left) with heart failure (right). Under homeostasis, Notch–HES1 signaling in Lepr⁺ mesenchymal stromal cells of the bone marrow niche maintains the HBEGF-expressing subpopulation and restrains the adipogenic regulator PPARγ, supporting balanced hematopoiesis and the generation of CCR2ˡᵒ macrophages that help maintain tissue homeostasis and protect multiple organs, including the heart, from stress. In heart failure, cardiac stress suppresses Notch–HES1 signaling, leading to loss of the HBEGF-expressing subpopulation and de-repression of PPARγ with adipogenic conversion of the stromal cells. The resulting decrease in HBEGF–EGFR signaling imprints a lasting memory on hematopoietic stem cells (stress-imprinted HSCs), establishing central innate immune memory that biases hematopoiesis toward CCR2ʰⁱ proinflammatory macrophages and drives long-lasting multimorbidity. A corresponding HBEGF⁺ mesenchymal stromal cell subpopulation is present in human bone marrow, and heart failure is associated with increased bone marrow adiposity in humans, indicating that this mechanism is conserved across species. Created in BioRender. F, K. (2026) https://BioRender.com/g0580kj.

To investigate the relevance of our findings in human HF, we analyzed publicly available scRNA-seq data from human BM niche cells^34^. Clustering of human *LEPR*^+^ MSCs and analysis of the marker gene expression patterns showed that the human H1 to H5 clusters correspond to the mouse C1 to C5 clusters (Fig. 5a, b, Supplementary Fig. 20a). Moreover, the expression patterns of the known marker genes in the human MSC clusters (Supplementary Fig. 20b) also largely matched those of mouse MSC clusters and suggested that H1 is adipo-primed, H3 is unprimed, H4 is fibro-primed, and H5 is osteo-primed. As in mouse C2 cells (Fig. 2b), *HBEGF* was expressed in H2 cells, which were also characterized by strong expression of *KLF4* and *ID2* as well as immediate early response genes, such as *IER5* (Fig. 5b, Supplementary Fig. 20a). Likewise, human H2 cells did not exhibit expression patterns of the known markers that conformed to previously identified MSC subpopulations, which was also true of C2 cells.

TF network analysis of human MSCs controlling *HBEGF* identified KLF4 as a network hub as in mouse MSCs (Supplementary Fig. 18b). Immediate early response TFs, such as FOS and EGR1, were inferred to directly regulate *HBEGF* as in mouse MSCs. In addition, HES1 was also identified in the TF network. These findings suggest that the structure of the human TF network is similar to that in mouse and that similar transcriptional programs control HBEGF expression across species.

To determine whether HF promotes BM adipogenesis in humans, we used coronary computed tomography (CT) to quantitatively evaluate the vertebral BM fat content in HF patients and healthy controls (Table 1, Supplementary Fig. 21).

**Table 1:** Characteristics of the patients enrolled in the retrospective cohort study

Patient characteristics (*N* = 257)		
Age (SD, years)		60.5 (19.2)
Male (%)		162 (63.4)
Patients with heart failure		15
(Etiology)	Ischemic heart disease	5
	Valvular heart disease	2
	Dilated cardiomyopathy	2
	Congenital heart disease	2
	Sarcoidosis	1
	Amyloidosis	1
	HFpEF	2

*SD*, standard deviation, *HFpEF*, heart failure with preserved ejection fraction.

In an age- and sex-matched analysis (Table 2), we detected a significant association between BM adipose tissue and history of HF (Fig. 5c).

**Table 2:** Characteristics in the age- and sex-matched cohort

	Control (*N* = 30)	HF (*N* = 15)
Age (SD, years)	67.8 (12.5)	67.7 (12.6)
Male (%)	18 (60.0)	9 (60.0)

*SD*, standard deviation, *HF*, heart failure.

The finding aligned with our observations in TAC-induced HF mice and suggested HF induced similar alterations in the BM niche across species. In addition, reanalysis of a scRNA-seq dataset of HSPCs from patients with a history of myocardial infarction (MI) undergoing coronary artery bypass graft surgery^54^ showed that EGFR and TGF-β signaling were downregulated in HSCs from MI patients (Supplementary Fig. 20c), consistent with our findings in TAC mice. These results further support the notion that alterations in the BM niche modulate these pathways, contributing to HSC responses to cardiac insults in humans.

## Discussion

This study demonstrated that cardiac stress promoted BM niche remodeling characterized by activation of the adipogenic program and reduction of the *Hbegf*^+^ MSC subpopulation. These changes affected HSPCs, leading to myeloid-skewed hematopoiesis and excessive accumulation of proinflammatory cardiac macrophages, ultimately resulting in cardiac remodeling and dysfunction. HBEGF expression in MSCs emerged as a key regulator in this process, as its downregulation was associated with changes in HSC differentiation and cardiac dysfunction. The findings that transplantation of TAC-MSCs and MSC-specific *Hbegf* knockdown each promoted myeloid-biased hematopoiesis and cardiac dysfunction strongly suggested that MSCs played a crucial role in shaping innate immune memory in HSPCs during HF.

It is increasingly recognized that the formation of innate immune memory in HSPCs (central innate immune memory) is a crucial mechanism in various chronic diseases, including cardiometabolic disease. The results of our co-transplantation experiments, which showed that HF-experienced MSCs can transfer their effects to healthy HSPCs, provided strong evidence for the role of MSCs in establishing innate immune memory. We previously showed that sympathetic nerve impairment induced by TAC suppresses Schwann cell-mediated TGF-β activation in the BM, leading to epigenetic alterations in HSCs in HF^15^. Other studies have demonstrated the involvement of IL-1β signaling in central innate immune memory after cerebral infarction^16^. These findings point to the notion that the BM niche orchestrates central innate immune memory through multiple pathways and highlight the complex cellular interactions that shape HSPC epigenetic memory. Future studies will need to elucidate how various stressors differentially activate these pathways and their potential crosstalk. For instance, it will be important to understand how the TGF-β and HBEGF pathways may interact to control MSC and HSPC responses during stress, and how these interactions contribute to the establishment of distinct types of innate immune memory. It will also be important to determine whether these mechanisms operate similarly across different cardiac insults, including MI. Several lines of evidence suggest mechanistic similarities between BM responses to TAC and MI. For instance, progressive HSPC proliferation and myeloid-biased hematopoiesis have been observed through 4 weeks after both TAC and MI^54^, and EGFR and TGF-β signaling were downregulated in HSCs from patients with a history of MI (Supplementary Fig. 20c). However, studies on acute MI suggest that some responses may differ. Acute MI triggers a rapid and transient increase in bone marrow sympathetic activity and marked reduction of retention factors such as CXCL12 and SCF, resulting in prompt HSPC mobilization^55^, which were not observed 4 weeks after TAC^15^. Further studies are needed to compare how hematopoietic lineage and niche cells respond to different cardiac insults across different time points, and how innate immune memory is shaped in various HF etiologies. Importantly, HF etiologies and presentations are divergent, and our findings may not be generalizable to all forms of HF. TAC induces pressure overload, leading to cardiac remodeling. In our experimental setting, this progressed to systolic dysfunction (HFrEF). In contrast, HF with preserved EF (HFpEF) is often associated with metabolic comorbidities such as obesity and diabetes, and the BM may respond to distinct signals including metabolic stress. Future studies will need to address how HF risk factors affect BM niche cells and innate memory formation.

HBEGF has been shown to induce EGFR-mediated signaling in both soluble/secreted and membrane-anchored forms. The latter transmits signals to adjacent cells via direct cell–cell contact, termed juxtacrine signaling^56^. We have not identified the mode of signal transmission by HBEGF in the present study. However, the proximity of *Lepr*^+^ MSCs to HSCs^57^ suggests the involvement of a juxtacrine mechanism. In juxtacrine signaling, membrane-anchored HBEGF associates with CD9 and integrin α3β1^58^, all of which are expressed in MSCs. Extracellular vesicles can also carry membrane-anchored HBEGF and may serve as vehicles for both paracrine and juxtacrine signaling^59^. Beyond HBEGF itself, extracellular vesicles released by Lepr⁺ MSCs could transfer a broader cargo of proteins, lipids and regulatory RNAs to HSPCs, potentially contributing to the epigenetic and transcriptional reprogramming that underlies innate immune memory. Such vesicle-mediated transfer could complement direct cell–cell contact, particularly given the close physical association between MSCs and HSCs in the niche, and might allow stress signals to reach HSPCs beyond the immediate perivascular microenvironment. Although addressing the specific contribution of Lepr⁺ MSC-derived extracellular vesicles in vivo remains technically challenging, future studies isolating and functionally characterizing these vesicles will be important to determine whether, and how, they participate in transmitting cardiac-stress signals to the hematopoietic compartment.　Future studies will be needed to determine the mode of HBEGF signaling and further characterize MSC-HSC communication in innate immune memory.

Another important question is whether the BM niche itself carries long-lasting memory. While systemic effectors of central innate immune memory are likely to be primarily HSPC progeny, BM niche cells may also retain memory of stress, similar to what has been observed in epithelial cells and fibroblasts in other contexts^60,61^. This possibility of innate immune memory in the BM would have important implications for understanding disease recurrence, progression, and interaction as well as drug development. In that regard, the increased adiposity due to skewed differentiation of MSCs may lead to lasting changes in the BM niche, potentially constituting innate immune memory in the BM tissue.

Analysis of TF regulon activity showed that TAC activated group 3 cluster-specific regulons, including PPARγ, C/EBPα, NFIB, and PBX1, which are important regulators of adipogenesis (Fig. 4a)^62–64^. Additionally, the activity of other key adipogenic regulators, such as KLF15, SREBF1 and FOXO1, was increased in MSCs after TAC (Supplementary Fig. 15). Along with the increased numbers of adipocytes across the BM in both sexes, these findings indicate that the adipogenic program is activated in MSCs after TAC. KLF4, a key regulator of HBEGF^+^ MSCs in both mouse and human, and PPARγ emerged as central hub TFs controlling MSC fate in HF. It should be noted that the scRNA-seq analyses of female mice showed that TAC similarly reduced the *Hbegf*⁺ MSC cluster and expanded the adipo-primed MSC cluster, suggesting that heart failure-induced adipogenic reprogramming of MSCs occurs regardless of sex. However, the functional consequences of heart failure-experienced MSCs, including their effects on HSC differentiation, myeloid skewing, and cardiac macrophage phenotypes, were examined only in male mice in the present study. Whether these effects are fully sex-independent remains to be determined.

While the mechanisms linking HF to MSC response are not fully understood, several lines of evidence point to sympathetic nerve dysfunction. We previously showed that impaired sympathetic nerve activity reduces TGF-β production by Schwann cells and subsequently promotes HSPC proliferation and skewed differentiation^15^. Maryanovich et al. showed that sympathetic denervation and constitutive deletion of *Adrb3* impair MSC function and HSC maintenance capacity^65^. These results suggest that sympathetic neuropathy is involved in the response of MSCs to HF. Our TF network analysis identified Hes1 as a link between KLF4 and PPARγ networks, and suggests that reduced Notch signaling in BM skews MSC differentiation after TAC, although how Notch signaling is impaired after TAC remains to be elucidated. Previous studies have shown that β-adrenergic receptor signaling controls Notch ligand expression in several cell types^66–68^, suggesting possible regulation of Notch signaling by sympathetic impairment in HF. Importantly, we previously showed by parabiosis experiments that circulating factors are not sufficient to induce hematopoietic changes in the parabiont of mice that underwent TAC^15^. Taken together, these findings strongly suggest that sympathetic nerve dysfunction serves as a key signal that communicates cardiac stress to the BM niche.

Finally, our analysis of human data indicated that the *HBEGF*^+^ MSC subpopulation and BM adiposity were associated with HF. Together with our mouse studies, these results established the BM niche as a critical component of HF progression and recurrence. Current HF therapies primarily target cardiac dysfunction directly, but our results suggest that the BM niche is a potential therapeutic target, though targeting the BM niche presents unique challenges, including the need for a delivery system and the potential systemic effects of modulating MSC function. Nonetheless, the increased BM adiposity may serve as a potential biomarker for disease progression as well as innate immune memory. In that regard, it will be important to assess BM adiposity in other chronic diseases that likely involve innate immune memory, including atherosclerosis.

In conclusion, this study identifies MSC-derived HBEGF as a critical regulator linking cardiac stress to hematopoietic reprogramming and highlights the BM niche as a promising therapeutic and diagnostic target in HF (Fig. 5e).

## Methods

### Ethics statement

This research complies with all relevant ethical regulations. All animal experiments were approved by the University of Tokyo Ethics Committee for Animal Experiments and were performed in accordance with the institutional guidelines for animal experiments at the University of Tokyo. The retrospective human cohort study was approved by the Research Ethics Committee of the Graduate School of Medicine, the University of Tokyo (reference no. 2650-14) and was conducted in accordance with the principles of the Declaration of Helsinki.

### Mice

C57BL/6J mice were purchased from Clea Japan. C57BL/6 mice congenic for the CD45 locus (CD45.1 mice) were purchased from Sankyo Labo Service Co (Tsukuba, Japan). B6.129-Lepr^tm3(cre)Mgmj^/J (♯032457) and B6.Cg-Gt(ROSA)26Sor^tm14(CAG-tdTomato)Hze^/J (#007914) were from The Jackson Laboratory (USA). To generate *Lepr-cre;LoxP-tdTomato* (LepR-tdTomato) mice, B6.129-Lepr^tm3(cre)Mgmj^/J was crossed with reporter mice containing a floxed transcriptional stop at the Rosa26 locus (B6.Cg-Gt(ROSA)26Sor^tm14(CAG-tdTomato)Hze^/J). Offspring from this cross are subsequently referred to as LEPR-tdTomato mice. All mice were housed in separate cages under specific pathogen-free conditions at the University of Tokyo animal facility, under a 12-h light/12-h dark cycle at an ambient temperature of 23 ± 2 °C and a relative humidity of 50 ± 10%. Mice received standard mouse chow and water ad libitum. All experiments were approved by the University of Tokyo Ethics Committee for Animal Experiments and strictly adhered to the guidelines for animal experiments at the University of Tokyo.

### Isolation of mesenchymal stromal cells (MSCs)

The femurs, tibiae, and pelvis were collected from mice and thoroughly cleaned to remove the adherent muscles^18,69^. The edges of the cleaned bones were then cut and the bones were flushed with cell suspension medium (2%FBS + PBS). The collected whole bone marrow was digested with collagenase type II (1 mg/mL) and DNase Ⅰ (25 U/mL) in HBSS for 30 min at 37 °C. Bone fragments were digested with collagenase type Ⅰ (2.5 mg/mL) (Sigma-Aldrich, C0130) and DNase I (25 U/mL) in HBSS for 45 min at 37 °C. After digestion, cells were washed with cell suspension medium and filtered through a 100 µm cell strainer (Greiner). After removing erythrocytes from the samples with PharmLyse solution (BD), Mouse CD45 MicroBeads (Miltenyi) and Mouse Lineage Cell Depletion Kit (Miltenyi) were added and incubated for 15 min at 4 °C in MACS buffer (PBS supplemented with 0.5% bovine serum albumin and 2 mM EDTA). The samples were then washed, filtered through a 40 µm cell strainer, and separated using an autoMACS (Miltenyi). The negatively selected cells were washed with FACS buffer (PBS supplemented with 5% FBS) and mixed with antibodies for flow cytometry. Lepr^+^ or tdTomato^+^ cells were sorted as MSCs. For single-cell RNA sequencing (scRNA-seq), MSCs were sorted twice to improve the purity of Lepr⁺ cells, first using yield mode followed by purity mode on the cell sorter. Both MACS buffer and FACS buffer were supplemented with actinomycin D (2 µM; Sigma-Aldrich) to minimize transcriptional artifacts during cell isolation.

### Isolation of cKit^+^ cells and Lin^-^Sca1^+^cKit^+^ (LSK) cells

Bone marrow cells were harvested from the tibiae, femurs and the pelvis by crushing the bone with a mortar and pestle in MACS buffer^70^. The cells were passed through a 23-gauge needle several times and filtered through a 70-μm cell strainer. After removing the erythrocytes from the samples with PharmLyse solution (BD), the cells were washed with MACS buffer and filtered through a 40 μm cell strainer (Greiner). Mouse CD117 Microbeads (Miltenyi) were added to the cells and incubated for 15 min at 4 °C. After washing and filtering through a 40-μm cell strainer, the samples were separated using autoMACS (Miltenyi). Isolated cells in the positive selection were washed and suspended in PBS. For the bone marrow transplantation described below, these cells were collected as cKit^+^ cells. For flow cytometric analysis of hematopoietic stem and progenitor cells (HSPCs) and myeloid progenitor populations, the isolated c-Kit⁺ cells were further stained with fluorochrome-conjugated antibodies and analyzed by flow cytometry.

For the scRNA-seq, LSK cells were collected using flow cytometry. After MACS sorting for cKit^+^ cells, samples were stained with appropriate antibodies (referred to “Flow cytometry” section). After staining, cell sorting was performed with a FACS Aria III instrument (BD). FACS buffer with actinomycin D (2 µM) was used for washing the samples and for cell suspensions.

### MSC transplantation

Transplantation of bone marrow cells supplemented with MSCs was carried out as follows, based on the previous reports^71^. Donor cells for MSCs were prepared by excluding hematopoietic-lineage cells and endothelial cells with a magnetic cell separation system (MACS), using Mouse CD45 MicroBeads (Miltenyi), Mouse CD31 MicroBeads (Miltenyi), and Mouse Lineage Cell Depletion Kit (Miltenyi). The preparation of cKit^+^ hematopoietic cells was described above. The cell suspensions were washed with PBS twice and resuspended in PBS, after which Y-27632 (FUJIFILM Wako Pure Chemical Production) was added to the MSC suspension to a concentration of 10 µM. Five-week-old recipient male mice whole-body irradiated at a dose of 9 Gy were then intravenously injected with 1 × 10^5^ cKit^+^ hematopoietic cells and 1 × 10^5^ MSCs per mouse.

### Cardiac tissue homogenization

Cardiac tissues were processed as previously described^15^ with minor modifications. Briefly, mice were anesthetized and intravenously injected with 2 mg biotin-conjugated anti-CD45 antibody. The thoracic cavity was opened, the heart was exposed, and 10 mL of PBS was perfused via the left ventricle. The whole heart was excised, and the atria and atrioventricular valves were removed. The biventricles were mechanically minced using curved scissors and enzymatically digested in 1 mL DMEM containing collagenase I (450 U/mL; Sigma-Aldrich), hyaluronidase (60 U/mL; Sigma-Aldrich), and DNase-I (60 U/mL; Sigma-Aldrich) for 45 min at 37 °C with gentle agitation. Following digestion, the suspension was vortexed for 20 s, passed through a 40-μm cell strainer, and washed with 12 mL of cold HBSS supplemented with 2% FBS and 0.2% BSA. Cells were centrifuged at 400 × *g* for 5 min, washed with PBS, and resuspended in FACS buffer.

### Flow cytometry

Cell suspensions in FACS buffer were first incubated with FcR blocking reagent (BioLegend) for 5 min at 4 °C and then with fluorochrome-conjugated antibodies.

To isolate MSCs, samples were first stained with Ter119-PerCP/Cy5.5 (clone TER-119, 116228, BioLegend), CD45-PerCP/Cy5.5 (clone 30-F11, 103132, BioLegend), CD71-BV421 (clone R17217, 113813, BioLegend), CD144-APC (clone BV13, 1380112, BioLegend), CD31-PE/Cy7 (clone 390, 102418, BioLegend), CD41-FITC (clone MWReg30, 133904, BioLegend), CD61-FITC (clone 2C9.G2, 104306, BioLegend), CD11b-FITC (clone M1/70, 101206, BioLegend), Gr1-FITC (clone RB6-8C5, 108406, BioLegend), CD45-FITC (clone 30-F11, 103108, BioLegend), CD45R-FITC (clone RA3-6B2, 103206, BioLegend), CD5-FITC (clone 53-7.3, 100606, BioLegend) and Leptin R Biotinylated Antibody (BAF497, R&D) for 60 min at 4 °C. This was followed by a second staining with antibodies against streptavidin-PE (405204, BioLegend).

To isolate LSK cells and LT-HSCs, samples were stained with CD45.2-V500 (clone 104, 562130, BD Biosciences), Sca1-PE (clone D7, 108108, BioLegend), cKit-APC/Cy7 (clone 2B8, 105814, BioLegend), CD34-FITC (clone RAM34, 553733, BD Biosciences), CD135-BV421 (clone A2F10, 135313, BioLegend), CD48-PE/Cy7 (clone HM48-1, 103424, BioLegend), CD150-BV785 (clone TC15-12F12.2, 115937, BioLegend) and the lineage markers CD4-PerCP/Cy5.5 (clone GK1.5, 100433, BioLegend), CD8a-PerCP/Cy5.5 (clone 53-6.7, 100734, BioLegend), CD11b-PerCP/Cy5.5 (clone M1/70, 101228, BioLegend), Ly6G-PerCP/Cy5.5 (clone 1A8, 127616, BioLegend), B220-PerCP/Cy5.5 (clone RA3-6B2, 103236, BioLegend), CD127-PerCP/Cy5.5 (clone SB/199, 121113, BioLegend), Ter119-PerCP/Cy5.5 for 90 min at 4 °C.

To analyze myeloid progenitor cells including granulocyte–monocyte progenitors (GMPs), cells were stained with CD45.2-V500 (clone 104, 562130, BD Biosciences), Sca1-PE (clone D7, 108108, BioLegend), cKit-APC/Cy7 (clone 2B8, 105814, BioLegend), CD34-FITC (clone RAM34, 553733, BD Biosciences), CD16/32-APC (clone 93, 101326, BioLegend), together with lineage markers identical to those used for LSK cell isolation, for 90 min at 4 °C.

To analyze cardiac macrophages, cells were first stained with CCR2-PE (FAB5538P, R&D Systems) for 15 min at 4 °C, followed by staining with CD45.2-BUV395 (clone 104, 564616, BD), CD11b-BV421 (clone M1/70, 101236, BioLegend), CD64-APC (clone X54-5/7.1, 139306, BioLegend), Ly6G-PE/Cy7 (clone 1A8, 127618, BioLegend), Ly6C-APC/Cy7 (clone HK1.4, 128026, BioLegend), Ter119-PerCP/Cy5.5 and Streptavidin-PerCP/Cy5.5 (405214, Biolegend) for 30 min at 4 °C.

After staining, the cell suspensions were washed twice with FACS buffer, resuspended in FACS buffer supplemented with propidium iodide or 4’,6-Diamidino-2-Phenylindole (DAPI) (BD, 564907), and subjected to flow cytometric analysis and cell sorting using a FACS Aria III (BD) or a FACS Aria Fusion (BD).

Initial gating was performed to exclude debris and dead cells. Forward scatter (FSC) and side scatter (SSC) parameters were used to identify the main cell population, followed by exclusion of dead cells using DAPI staining (BD Biosciences, 564907). Doublets were excluded based on FSC-A/FSC-H gating. Hematopoietic populations and MSCs were defined using established marker combinations. The results were analyzed using FlowJo (BD).

### Analysis of proliferating HSCs

Whole-body irradiated recipient C57BL/6J mice were intravenously injected with 1 × 10⁵ c-Kit⁺ hematopoietic cells isolated from CD45.1 mice together with 1 × 10⁵ MSCs from control or TAC B6J mice. To label cells that entered S phase during the preceding 24 hours, recipients were intraperitoneally injected with 30 μg/g body weight of 5-ethynyl-2′-deoxyuridine (EdU) 24 h before euthanasia. Bone marrow cells were harvested from femurs as described above. After enrichment of CD117⁺ cells using MACS (Miltenyi Biotec), cells were stained with antibodies against CD45.1-BV711, Sca-1-PE, c-Kit-APC/Cy7, CD34-FITC, CD135-BV421, CD150-BV786, and lineage markers (CD4, CD8, CD11b, Ly6G, B220, CD127, Ter119)-PerCP/Cy5.5 for 90 min at 4 °C. Cells were then processed using the Click-iT Plus EdU Alexa Fluor 647 Flow Cytometry Assay Kit (Thermo Fisher Scientific) according to the manufacturer’s instructions. The percentage of EdU-positive cells among CD45.1⁺Lin⁻Sca-1⁺c-Kit⁺CD34⁻Flt3⁻CD150⁺ HSCs was determined by flow cytometry. Data were acquired using a FACS Aria Fusion (BD Biosciences).

### Transverse aortic constriction (TAC)

TAC was performed as described previously^72^. In brief, 8-week-old male mice or 11-week-old female mice were anesthetized, then intubated and ventilated using a volume-cycled rodent ventilator with a tidal volume of 0.4 mL of room air and a respiratory rate of 100 breaths/min. The heart was exposed by cutting the proximal portion of the sternum. The aorta was constricted between the innominate and left common carotid arteries by placing a 7-0 nylon suture against a blunted needle (26-gauge for males, 27-gauge for females). Sham-operated mice underwent the same anesthesia and surgical procedures without aortic constriction.

### Echocardiography

Echocardiography was performed on awake, non-anesthetized mice. Mice were gently restrained in a supine position, and heart rates were maintained between 650–700 beats per minute during image acquisition. Transthoracic M-mode echocardiography was performed to assess cardiac dimensions and contractility using a Vevo3100 imaging system (FUJIFILM, VisualSonics). The mid-portion of the left ventricle, containing the papillary muscles, was imaged in the parasternal short-axis view. Interventricular septum thickness (IVST), left ventricular diastolic dimension (LVDd), left ventricular systolic dimension (LVDs), and left posterior wall thickness (PWT) were measured. The left ventricular ejection fraction (LVEF) was calculated using the Teichholz method.

### **BNP measurement with ELISA**

To measure BNP, whole blood was collected through retro-orbital bleeding into tubes coated with K3-EDTA (Greiner), after which plasma was separated by centrifugation for 10 min at 1000 × *g* at 4 °C. Plasma BNP levels were measured using a RayBio Mouse BNP Enzyme Immunoassay Kit (RayBiotech, Catalog #: EIAM-BNP) according to the manufacturer’s instructions. All samples were diluted 8-fold and applied in duplicate.

### Lung wet-to-dry weight ratio

The right and left lung lobes were excised, briefly blotted to remove excess surface fluid, and immediately weighed to obtain the wet weight. The tissues were then dried in a constant-temperature oven at 60 °C overnight, after which the dry weight was measured. The wet-to-dry weight ratio was calculated as the ratio of wet weight to dry weight.

### Purification of RNA and real-time PCR

Total RNA was purified from tissues and cells using RNeasy and RNeasy Plus Micro kits (Qiagen) according to the manufacturer’s instructions^73^. Prior to RNA sequencing, the cell lysates were passed through a gDNA Eliminator spin column to eliminate potential contamination by genomic DNA. Total RNA was converted to cDNA using Superscript III (Thermo Fisher). Quantitative real-time PCR analyses were carried out using a Lightcycler 480 system (Roche) with 18S rRNA serving as an internal control. The sequences of the primers used are as follows: *18s*, 5’-AAA CGG CTA CCA CAT CCA AG-3’ and 5’-CGC TCC CAA GAT CCA ACT AC-3’; *Hbegf*, 5’-GAG TTC CGT ACT CCC TCT TGC A-3’ and 5’-CAG CCA AGA CTG TAG TGT GGT C-3’; *Col1a1*, 5’-CAT GTT CAG CTT TGT GGA CCT-3’ and 5’-GCA GCT GAC TTC AGG GAT GT-3’; *Col1a2*, 5’-CAA GCA TGT CTG GTT AGG AGA G-3’ and 5’-AGG ACA CCC CTT CTA CGT TGT-3’; *Col3a1*, 5’-TCC CCT GGA ATC TGT GAA TC-3’ and 5’-TGA GTC GAA TTG GGG AGA AT-3’; *Fn1*, 5’-CGA GGT GAC AGA GAC CAC AA-3’ and 5’-CTG GAG TCA AGC CAG ACA CA-3’; *Fabp4*, 5’-TCA CCA TCC GGT CAG AGA GTA-3’ and 5’-GCC ATC TAG GGT TAT GAT GCT C-3’; *Adipoq*, 5’-GTT CCT CTT AAT CCT GCC CA-3’ and 5’-CTC CTG TCA TTC CAA CAT CTC-3’; *Pparg*, 5’-AAG ACA ACG GAC AAA TCA CCA-3’ and 5’-GGG GGT GAT ATG TTT GAA CTT G-3’; *Plin1*, 5’-TGA AGG GTG TTA CGG ATA ACG-3’ and 5’-ATG TCT CGG AAT TCG CTC TC-3’; *Wif1*, 5’-CTT TGC TGG GAA CAG TGC CTC A-3’ and 5’-GGT CCT AAG GAT GGT GTT GCC T-3’; *Ier5*, 5’-GAA CTC GGA GGA GCA GCC AGT-3’ and 5’-CTT TTC CGT AGG AGT CCC GAG A-3’; *Nppa*, 5’-CCT AAG CCC TTG TGG TGT GT-3’ and 5’-CAG AGT GGG AGA GGC AAG AC-3’; *Nppb*, 5’-CTG AAG GTG CTG TCC CAG AT-3’ and 5’-CCT TGG TCC TTC AAG AGC TG-3’; *tdTomato*, 5’-CTG TTC CTG TAC GGC ATG G-3’ and 5’-TCT TTG ATG ACG GCC ATG T-3’.

### Histology

Anesthetized mice were perfused with 10 mL of PBS via the left ventricle, after which the femurs and coccyx were excised and fixed in 10% neutral buffered formalin (NBF) (FUJIFILM Wako Pure Chemical Production). After samples were decalcified in KC-X (FALMA) for 1–2 h and neutralized in 5% sodium sulfate (FUJIFILM Wako Pure Chemical Production) for 30 min, they were paraffin-embedded and cut into 4-µm-thick sections. The sections were then dewaxed and stained with hematoxylin/eosin. The stained sections were dehydrated in absolute alcohol, cleared in xylene, and permanently mounted. To measure the number of bone marrow adipocytes in histology, a microscope (Nikon) equipped with a 4x objective was used to examine the distal portion of the femurs. One of the authors measured the number of Plin1-positive bone marrow adipocytes visualized using HRP-DAB. Inappropriate samples in which some parts of bone marrow were lost during sample preparation were removed from analysis. Only clearly visualized samples were assessed.

Photos of heart tissue were taken using an Olympus APX100 microscope. One transverse section of the heart cut at the papillary muscle level was used for evaluation of fibrosis. Picro-Sirius red-stained areas of cardiac tissues were detected using Image J (version 1.54j, Wayne Rasband, NIH).

### Frozen sample preparation

Sample preparation was as previously described^74^. Anesthetized mice were perfused with 10 mL of PBS followed by 10 mL of 4% paraformaldehyde (PFA) (FUJIFILM Wako Pure Chemical Production). Bones were post-fixed with 4% PFA for 2 h at room temperature. For cryopreservation, the bones were incubated sequentially in 30% sucrose/PBS overnight at 4 °C, then embedded and flash-frozen in SCEM embedding medium (Section-Lab). Frozen sections were cut into 12-μm-thick sections with a Cryostat (CM3050S, Leica) using Kawamoto’s tape transfer method. The staining procedure is described in the following section.

### Immunohistochemistry

Prior to immunohistochemistry, sections were deparaffinized in xylene and hydrated in an ethanol gradient and distilled water. For immunohistochemical staining of PLIN1, the sections were incubated with 200-fold diluted anti-PLIN1 antibody (Cell Signaling, 9349S) overnight at 4 °C. The sections were then washed and incubated for 1 h with horseradish peroxidase (HRP)-conjugated anti-rabbit IgG (414341, Nichirei Bioscience). PLIN1 signals were visualized using HRP-DAB (EnVision detection kit, Dako). Nuclei were counterstained with hematoxylin.

To observe fluoroimmunochemical sections stained with PLIN1, antigen retrieval was performed in 10 mM sodium citrate buffer (pH 6.0, 15-min, 90–95 °C) prior to blocking in 2% bovine serum albumin (BSA) (Themofisher). Sections were stained with antibodies against PLIN1 (1:200, Cell Signaling, 9349S) in 1.0% BSA for 2 h at room temperature. Secondary antibody (1:200 anti-rabbit Alexa Fluor 555; Thermofisher) in 1.0% BSA was applied for 1 h at room temperature. All washes between steps were performed in purified water. Nuclei were counterstained with DAPI Fluoromount-G (Southern Biotech).

For frozen samples, sections stuck on a glass slide were let dry for at least 30 min, blocked with 2% BSA for 10 min, and incubated with a primary antibody of 200-fold diluted anti-PLIN1 rabbit monoclonal antibody (Cell Signaling, 9349S) in 1% BSA for 2 h at room temperature. After washing, the samples were incubated with secondary antibodies for 1 h at room temperature. For the secondary antibody, donkey anti-rabbit Alexa Fluor 555 (Thermofisher) in 1.0% BSA was used. After washing the samples with PBS, nuclei were counterstained with DAPI Fluoromount-G (SouthernBiotech).

Fluorescent signals were visualized and captured using a confocal laser scanning microscope (FV3000, OLYMPUS).

### In vitro adipogenic differentiation assay

MSCs were cultured as previously described with some modifications^49,50^. Femurs, tibiae, and the pelvis were collected and cleaned thoroughly to remove the adherent muscles, after which the edges of the cleaned bones were cut and the bones were flushed with PBS. The collected samples were passed through a 23-gauge needle several times and filtered through a 70 μm cell strainer. After removing the supernatant solution, unfractionated bone marrow cells were resuspended in culture medium (DMEM (Gibco, 11995-065), 20% FBS (HyClone, SH30396.03), penicillin/streptomycin 50 U/mL, Y27632 10 µM (FUJIFILM Wako Pure Chemical Production, 030-24021)). Cells were plated at a density of 2 × 10^6^ cells/well in 6-well plates (CELLSTAR, 657-160) and incubated for 10 days at 37 °C, 5% CO_2_ without changing the medium. On day 10, the cells were subjected to one passage using 0.25% trypsin, and plated in 6-well plates (CELLSTAR, 657-160), 48-well plates (FALCON, 353078) or 8-well chamber slides (WATSON, 192-008). For adipogenic differentiation, cells were incubated in the same medium described above for the first 2 days, after which they were incubated in StemPro™ Adipogenesis Differentiation Kit (Thermofisher, A1007001) for 7 days with the medium was changed every other day. The cells were then subjected to cytostaining, absorbance measurements and the RNA extraction.

For adipocytes staining, the cells were fixed in 2% paraformaldehyde (MUTO Pure Chemicals Co., 15291) for 10 min, washed with 60% 2-propanol (isopropyl alcohol) (FUJIFILM Wako Pure Chemical Production, 166-04836), stained with oil red O (MUTO Pure Chemicals Co., 40491) for 15 min, and then immediately washed with distilled water. Cell counts in 8-well chamber slides were done visually with a microscopy (Nikon). For absorbance measurements, we extracted the oil red O with 100% isopropyl alcohol for 5 min with gentle rocking in 48-well plates, and the optical density was measured at 530 nm. To subtract the background signal, 100% isopropanol was used as a background control. We performed RNA extractions in the same manner described above, using RLT plus (Qiagen) in 6-well plates.

### Validation of Cre-dependent sh*Hbegf* knockdown in vitro

To validate the efficiency of Cre-dependent sh*Hbegf*-mediated knockdown, we established an in vitro system engineered to overexpress both *Cre* recombinase and murine *Hbegf*. The coding sequences of *Cre* and murine *Hbegf* were cloned under the control of the CMV promoter and packaged into lentiviral vectors. NIH-3T3 cells were transduced with the lentivirus and selected with puromycin to generate stable cell lines overexpressing *Cre* and *Hbegf*. To assess knockdown efficiency, cells were transfected with the AAV-FLEX–sh*Hbegf* or scramble transgene plasmid using Lipofectamine 2000 (Thermo Fisher Scientific) according to the manufacturer’s protocol. Total RNA was harvested 48 h after transfection, and *Hbegf* mRNA levels were quantified by quantitative real-time PCR. Expression levels were normalized to 18S expression.

### AAV vector production and administration

The plasmids pAAV2/9n (#112865) and pAdΔF6 (#112867) were obtained from Addgene. The plasmids pAAV[FLEXon]-CAG > LL:rev(EGFP–sh*Hbegf*):rev(LL):WPRE and pAAV[FLEXon]-CAG > LL:rev(EGFP-scramble shRNA):rev(LL):WPRE were purchased from VectorBuilder. The target sequence for sh*Hbegf* was CTCCGTACTC CCTCTTGCAA ATTAGTGAAG CCACAGATGT AATTTGCAAG AGGGAGTACG GAA, and the scramble control sequence was ACCT AAGGTTAAGT CGCCCTCGTA CATCTGTGGC TTCACTACGA GGGCGACTTA ACCTTAGGG. AAV9 vectors were produced using the AAV-MAX system (Thermo Fisher Scientific) according to the manufacturer’s protocol. Briefly, 293F-derived Viral Production Cells 2.0 cultured in a total volume of 60 mL medium were co-transfected with pAAV2/9n, pAdΔF6, and the respective transgene plasmids using AAV-MAX Transfection Booster and AAV-MAX Transfection Reagent. Seventy-two hours after transfection, cells and medium were collected and treated with AAV-MAX Lysis Buffer (Gibco, A50520) to release viral particles. Viral particles were purified by iodixanol density gradient ultracentrifugation, and virus-containing fractions were collected from the 40% iodixanol layer. The collected fraction was further concentrated and buffer-exchanged using Amicon™ Ultra-15 centrifugal filter units (Millipore) and sterilized through a 0.22-µm GV filter. Viral genome titers were determined by quantitative real-time PCR targeting the inverted terminal repeat (ITR) sequence. For in vivo experiments, a single dose of 2.0 × 10^12^ genome copies of AAV9 in 200 µL PBS was intravenously injected into the tail vein of each mouse. Two weeks after AAV administration, TAC surgery was performed.

### Primary MSC isolation and siRNA-mediated *Hbegf* knockdown

Primary bone marrow-derived MSCs were isolated from murine femurs and tibiae. Bones were fragmented and subjected to enzymatic digestion with 1.5 mg/mL collagenase type IV (Worthington Biochemical Corporation) and 0.1 mg/mL DNase I (Sigma-Aldrich) at 37 °C for 60 min with mechanical agitation, as previously described^75^. The resulting cell suspension was filtered through 100-μm cell strainers (Corning) to remove bone fragments and obtain a heterogeneous bone-derived cell population. Cells were cultured in complete MSC medium consisting of MesenCult basal medium (STEMCELL technologies) supplemented with 10% MesenCult supplement, 0.1% MesenPure, 1% L-glutamine, 100 U/mL penicillin, and 100 μg/mL streptomycin. Cells were seeded at 2.0 × 10^5^ cells in 2 mL per well in 6-well plates and cultured with medium replacement every 5 days. After two passages, cells were plated at 1.0 × 10^5^ cells per well in 24-well plates for knockdown experiments.

For gene silencing, cells were transfected with predesigned Silencer Select siRNA targeting murine *Hbegf* (Thermo Fisher) or Silencer Select Negative Control No. 1 siRNA at a final concentration of 10 nM using Lipofectamine RNAiMAX (Thermo Fisher), according to the manufacturer’s protocol. Total RNA was harvested 48 hours after transfection for quantitative real-time PCR analysis.

### Colony-forming-unit (CFU) assays

HSCs isolated from BM of C57BL/6J mice were cultured ex vivo as previously reported^76^. Briefly, flow-cytometrically sorted Lin^-^CD45.2^+^Sca1^+^cKit^+^CD150^+^ HSCs were resuspended in HemEx™-Type9AØ (Cell Science & Technology Institute) supplemented with 10 ng/mL Stem Cell Factor (579702, BioLegend), 100 ng/mL Thrombopoietin (593302, BioLegend) and penicillin–streptomycin (Gibco), and plated in fibronectin-coated 24-well flat-bottom plates (Corning) at 100–1000 cells per well. Cells were replated every 3–5 days, and detached cells are analyzed by flow cytometry. CFU assay was performed using MethoCult GF M3434 (ST-03434, StemCell Technologies) according to the manufacturer’s instructions. A total of 4000 Sca1^+^cKit^+^CD150^+^ cultured HSCs were directly sorted into 4 mL of aliquots of MethoCult medium. After supplementation with a total 400 µL consisting of penicillin–streptomycin solution, PBS and the indicated amount of recombinant mouse HB-EGF (785704, BioLegend), the cell suspension was vortexed thoroughly and divided into three wells of 12-well plates. For experiments using bone marrow extracts, femoral bone marrow was flushed with 500 µL of PBS and supernatants were collected after centrifugation. A total of 400 µL of bone marrow extract was added to 4 mL of MethoCult medium containing the cultured HSCs before plating. Plates were incubated at 37 °C for 7 days, and whole-well images were captured using APX100 imaging system (Evident). Colonies were counted by experienced investigators in a blinded manner and classified based on size, color, and morphological complexity.

### Bulk RNA sequencing

RNA sequencing was performed as previously described^73^. Total RNA was purified from cells using RNeasy and from tissues using RNeasy Plus Micro kits (Qiagen) according to the manufacturer’s instructions. Prior to RNA sequencing, the cell lysates were passed through a gDNA Eliminator spin column to eliminate potential contamination by genomic DNA. Poly A mRNA was extracted from the total RNA with Oligo dT beads from a NEBNext Poly(A) RNA Magnetic Isolation Module (NEB), after which RNA-seq libraries were prepared using a NEBNext Ultra II RNA Library Prep Kit for Illumina (NEB) according to the manufacturer’s protocols. Libraries were single-end-sequenced or paired-end-sequenced on a HiSeq 1500 sequencer (Illumina). Reads were aligned to the mm10 mouse genome using STAR^77^. Aligned read files were analyzed using HOMER^78^. Differential expression analysis was performed using DESeq2. Gene set enrichment analysis^48^ was performed using GSEApy^79^.

### Single-cell RNA sequencing

For single-cell RNA-seq (scRNA-seq) of MSCs, single-cell suspensions of LepR^+^ cells were prepared from control (*n* = 3) and TAC (*n* = 8) mice and labeled using hashtag antibodies (BioLegend #155831, #155833, #155835, #155837, #155839, #155841, #155843, #155845). These suspensions were then processed using a 10x Genomics platform with Chromium single cell 3’ reagent version 3.1. The sequence data were processed using Cell Ranger and mapped to mm10. This was followed by data filtering and analysis performed mainly on Seurat version 4 and 5^80^ in part through a modified version of SCALA^81^ a web-based interface (https://github.com/Manabe-lab/SCALAplus). We excluded cells when they expressed fewer than 586 unique molecular identifiers (UMI) and the percentage of counts mapped to the mitochondrial genome was ≥5%. To exclude possible doublets, cells with high UMI counts (≥3500) were also excluded. The remaining 3508 control and 4477 TAC cells were analyzed further. Datasets for control and TAC cells were aggregated using fastMNN to remove batch effects^82^. Clusters were identified using a graph-based approach with the Louvain modularity optimization algorithm. We employed Uniform Manifold Approximation and Projection (UMAP) for dimensionality reduction and visualization of our datasets. Initial clustering of MSCs identified a cluster that had fewer UMIs than other clusters and did not conform to the known marker gene expression patterns of MSCs. Because it was likely that the cluster represented low-quality cells, we removed it, and the remaining 3548 control and 4477 post-TAC cells were used in the following analyses. For analysis of genes differentially expressed between control and TAC mice, the log-normalized data from the MSC clusters (clusters 1,2,3,4,5) were analyzed using MAST^83^.

For single-cell RNA-seq (scRNA-seq) of LSK cells, single-cell suspensions of LSK cells were prepared from control or TAC mice and processed using a 10x Genomics platform with Chromium single cell 3’ reagent version 3.1. The sequence data were processed using Cell Ranger and mapped to mm10. This was followed by data filtering and analysis performed mainly with Seurat version 4 through SCALA. We excluded cells when they expressed fewer than 941 unique molecular identifiers (UMI) and the percentage of counts mapped to the mitochondrial genome was ≥7%. To remove possible doublets, cells with high UMI counts (≥3272) were also excluded. The remaining 10,351 control and 8167 TAC cells were analyzed further. We identified HSPC subpopulations based on the expression patterns of marker genes identified in previous reports^26,35,36^.

We used NicheNet^37^ to analyze MSC-to-LSK communications related to the differentially expressed genes in LSKs after TAC. We identified differentially expressed genes in LSKs (LT-HSC and MPP1-4 of the scRNA-seq dataset) through pseudobulk-edgeR analysis^84^. Differentially expressed genes (DEGs) with a false discovery rate (FDR) < 0.05 were used for NicheNet analysis. A high-quality subset of the OmniPath^85^ database was used for the ligand-receptor interactions, as described previously (https://r.omnipathdb.org/articles/nichenet.html). The top 30 ligands were detected with NicheNet^37^.

RNA velocity was analyzed using velocyto^86^ and the dynamical model of scVelo^87^. In addition, we used Dynamo^53^ for in silico perturbation analysis to predict the effects of transcription factor perturbation on MSC differentiation. For *Hes1* knockdown simulation, gene expression of *Hes1* was set to zero, and the resulting changes in the vector field were visualized on UMAP.

For pseudobulk differential expression analysis, we generated pseudobulk data using muscat^88^. As reported previously^84^, we used edgeR^38^ on the pseudobulk count data. For enrichment analysis of PROGENy pathways^89^, we used Python implementation of decoupler^90^ and analyzed ranking files based on the edgeR results using a multivariate linear model.

We used pySCENIC^91^ to infer gene regulatory networks. To identify cluster-specific transcriptional regulators, we first selected the top 5 regulons ranked on their RSS (regulon specificity score) for each cluster in the control and post-TAC datasets. For the heatmap in Fig. 4a, the average regulon activity scores for each cluster were scaled, and the top regulons were hierarchically clustered based on their activity patterns across clusters and conditions. The regulons were divided into six groups by cutting the dendrogram from hierarchical clustering analysis.

The transcriptional regulatory network centered on HBEGF was constructed using SCENIC regulon data to identify direct regulatory relationships between transcription factors. The connection specificity index (CSI), which is a measure of connectedness between regulons, was calculated as previously described (https://github.com/FloWuenne/scFunctions), and CSI scores were used to quantify the strength of the regulatory relationships. The network was filtered using ChIP-Atlas target gene data with a minimum binding score threshold of 20^92^, so that the regulatory edges lacking experimental support for direct binding were removed. Network centrality analysis was performed to identify key regulatory hubs. Weighted degree centrality was calculated by summing the CSI scores of incoming and outgoing edges. Weighted betweenness centrality was calculated using CSI scores as edge weights to measure nodes’ frequency on the shortest paths between other nodes in the network. To identify the most significant nodes, we calculated weighted degree and betweenness centrality for each node and nodes were ranked based on each metric. Nodes appearing in the top 10th percentile for both metrics were selected as network hubs. Network visualization and analysis were conducted using the SCENIC Network module in our NGS analysis suite, METIS.

For human MSCs, we reanalyzed publicly available human BM niche scRNA-seq datasets (GEO accession number, GSE253355)^34^. We used the data precomputed for Seurat. We used fastMNN^82^ to remove batch effects and assembled the 12 human datasets. After initial clustering, we subsetted the data for *LEPR*^+^ clusters (10328 cells). We then characterized the clusters based on expression of the marker genes we identified for mouse *Lepr*^+^ MSCs (Fig. S4). Two clusters that showed similar gene expression patterns were merged as H1. SCENIC analysis was performed as described for the mouse datasets.

For human HSPCs, we analyzed a publicly scRNA-seq dataset of human BM HSPC from patients undergoing coronary artery bypass graft surgery (ArrayExpress, E-MTAB-13509)^54^. Raw FASTQ files were processed with Cell Ranger (10x Genomics) and aligned to the GRCh38 reference genome. Cells were excluded if they had <300 total UMI counts or >9000 total UMI counts, or if the fraction of mitochondrial counts was ≥10%. After quality control, cells were annotated using BoneMarrowMap^93^, and cells labeled as HSC were retained for downstream analyses. For pseudobulk differential expression analysis, we generated pseudobulk count matrices by summing UMI counts across all HSCs within each 10x library, using the decoupleR framework (Python implementations)^90^ via the METIS pipeline (https://github.com/Manabe-lab/metis). E-MTAB-13509 comprises five 10x libraries: two control pooled libraries and three MI libraries (one pooled and two single-donor libraries). We treated each 10x library as a biological replicate (control *n* = 2, MI *n* = 3) and performed differential expression at the library level. Differential expression between MI and control pseudobulk samples was performed using DESeq2^94^ with svaseq^95^ to remove batch effects.

### Human data collection

For our retrospective cohort study, 257 patients who underwent coronary computed tomography between January 2024 and August 2024 were enrolled. Fifteen patients for whom electrical records showed a diagnosis of heart failure were selected. Age- and sex-matched healthy control were also included in this cohort at a ratio of 2:1. Axial slices at the level of Th7 were used to assess bone marrow fat content. Fat tissue in vertebral bone marrow was identified as the area where the CT value (Hounsfield unit) was less than zero. Data acquisition and quantification were performed using Intuition software (version 4.10, TeraRecon, Inc.). The proportion of patients who had fat deposition was compared between the healthy controls and heart failure patients. The study was conducted with the approval of the Research Ethics Committee of the Graduate School of Medicine, the University of Tokyo (reference no. 2650-14). Because this was a retrospective study using existing, de-identified clinical imaging data, the requirement for written informed consent was waived by the ethics committee; instead, an opt-out approach was used, whereby information about the study was made publicly available and patients were given the opportunity to decline participation. The sex of participants was obtained from clinical records; both male and female patients were included, and control patients were matched to heart failure patients by age and sex. Sex was considered in the study design through this matching, but the study was not powered to perform sex-based subgroup analyses.

### Statistics

The sample sizes were not based on power calculations. No animals were excluded from the analyses except for the inappropriate samples. Investigators who performed animal experiments were blinded to the allocation during experiments and assessed outcomes, but they were not blinded to the genotypes during surgery or whether the mice had undergone sham or TAC operations. Animals were randomly assigned to experimental groups.

Homogeneity of variances was assessed using the F-test for comparisons between two groups and Bartlett’s test for comparisons among multiple groups. Two-group comparisons were performed using two-tailed Student’s *t*-test for normally distributed data, and Mann–Whitney U-test for non-normally distributed data. Multiple-group comparisons with a single control group were analyzed using one-way analysis of variance (ANOVA) followed by Dunnett’s post-hoc tests. Comparisons of proportional data were analyzed after logit transformation. Non-parametric data were analyzed using the Kruskal–Wallis test, and categorical variables were analyzed using the chi-square test. A *P* value < 0.05 was considered statistically significant. Data are presented as means ± S.D. unless otherwise indicated.

To compare cluster distributions between control and TAC groups, we first transformed the raw cell counts into proportions by dividing each count by the total number of cells in each sample. To analyze this proportional data, we employed beta regression analysis. Beta regression models were fitted independently for each cell type using the BetaModel in the statsmodels package in Python (version 3.11). The models included group (Control vs. TAC) as a fixed effect, and statistical significance was assessed using Wald tests for the group effect coefficients. To account for multiple comparisons across the six cluster types, we applied Benjamini–Hochberg (BH) corrections to control the FDR. Results were considered statistically significant at an adjusted *p*-value < 0.05.

In figures, box-and-whisker plots show the median, 25th and 75th percentiles, and the minimum and maximum values.

Statistical analyses were performed using GraphPad Prism 9 software (GraphPad Software). Graphs were created using GraphPad Prism, Microsoft Excel, Adobe Illustrator, and BioRender.

### Reporting summary

Further information on research design is available in the Nature Portfolio Reporting Summary linked to this article.

## Supplementary information

Supplementary Information

Reporting Summary

Transparent Peer Review file

## Source data

Source data

## Data Availability

The sequencing data generated in this study have been deposited in the Gene Expression Omnibus (GEO) under accession numbers GSE326308 (bulk RNA-seq of male murine MSCs for Fig. 4b, c), GSE326120 (scRNA-seq of male murine HSPCs and MSCs for Fig. 2), and GSE326121 (scRNA-seq of female murine MSCs for Supplementary Fig. 8), including both raw sequencing data and processed scRNA-seq data. Publicly available datasets used in this study include GSE253355 (human bone marrow niche scRNA-seq) and E-MTAB-13509 (human bone marrow HSPC scRNA-seq). The source data underlying all figures, including the quantified microscopy and flow cytometry measurements, are provided with this paper in the Source data file. The corresponding raw image files and flow cytometry data files (.fcs) are not deposited in a public repository owing to their large size and the absence of a discipline-specific repository for these data types; they are available from the corresponding author (K.F., fujiu-tky@umin.ac.jp) upon request, with an expected response time of 2 weeks, for the purpose of verifying the findings of this study. Clinical imaging data are available under restricted access. These data are not publicly available because they contain potentially identifying patient information and their sharing is restricted by institutional policy and the ethical approval governing the study. Access requests should be directed to the corresponding author (K.F., fujiu-tky@umin.ac.jp). Access can be granted to qualified researchers for non-commercial research purposes, subject to approval by the relevant institutional review board and completion of a data-transfer agreement. Requests will be answered within approximately one month, and data will be available for the duration required to verify the findings of this study. Source data are provided with this paper.

## Code availability

The custom code used in this study is publicly available on GitHub and archived on Zenodo. The modified version of SCALA used for single-cell RNA-seq analysis (SCALAplus) is available at https://github.com/Manabe-lab/SCALAplus and archived at Zenodo (10.5281/zenodo.20566705). The METIS pipeline used for network and pseudobulk analyses is available at https://github.com/Manabe-lab/metis and archived at Zenodo (10.5281/zenodo.20566713). The connection specificity index was calculated using publicly available code at https://github.com/FloWuenne/scFunctions.
