# Supplementary material for: Altered bone marrow niche forms central innate immune memory driving cardiac dysfunction
Source: Nat Commun. 2026 Aug 26;17:8261. doi: 10.1038/s41467-026-76178-z (PMC13518785; doi:10.1038/s41467-026-76178-z)
Supplement: Supplementary file 1 [file 41467_2026_76178_MOESM1_ESM.pdf]

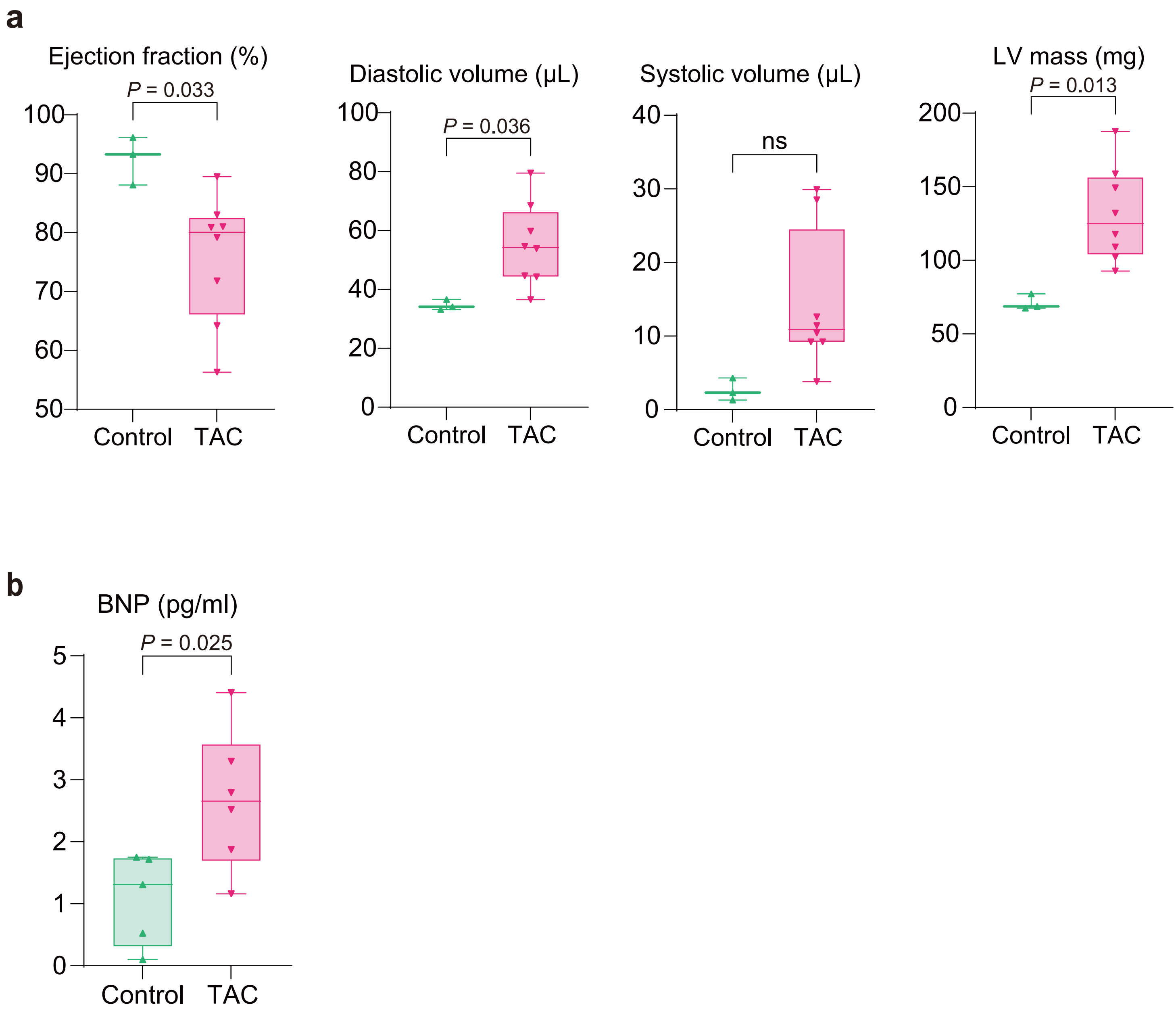

Supplementary Figure 1

Supplementary Figure 1. Cardiac dysfunction caused by TAC procedure.

a. Echocardiographic assessment of cardiac function in male mice 4 weeks after sham or TAC surgery. $n = 3$ for control, $n = 8$ for TAC. Two-tailed unpaired Student's t-test.

b. Plasma BNP levels in sham- and TAC-operated mice. $n = 5$ for control, $n = 6$ for TAC. Two-tailed unpaired Student's t-test.

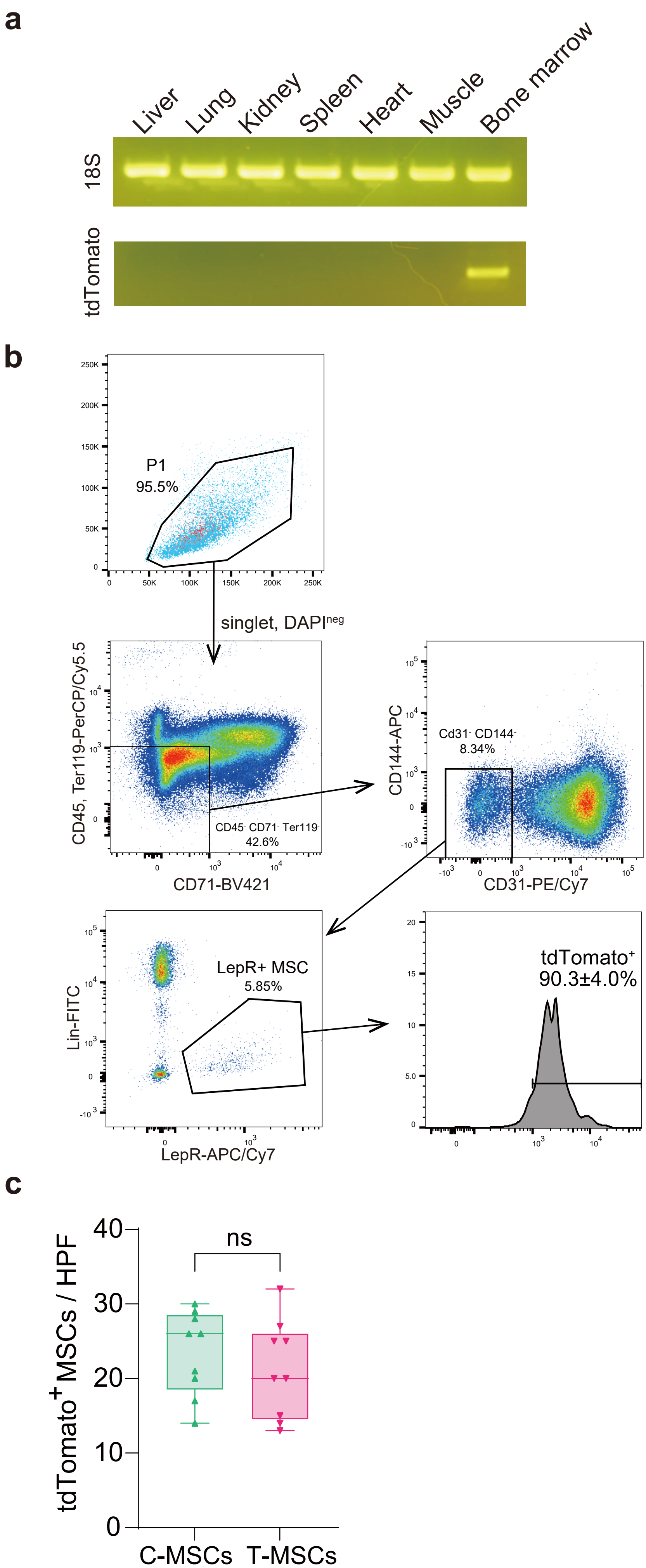

Supplementary Figure 2

Supplementary Figure 2. Engraftment of MSCs in tissues after co-transplantation.

a. RT-PCR analysis of *tdTomato* mRNA expression in the liver, lung, kidney, spleen, heart, muscle, and bone marrow of a recipient of $cKit^+$ BM cells and C-MSCs.

b. Flow cytometric analysis of donor-derived MSCs in recipient BM, including gating strategy for identifying $LepR^+$ MSCs. The proportion of $tdTomato^+$ donor-derived cells within $LepR^+$ MSCs was quantified.

c. Quantification of $tdTomato^+$ MSCs per high-power fields (HPF) seen in immunofluorescent staining of recipient femurs, corresponding to Fig. 1b. $n = 9$. Two-tailed unpaired Student's *t*-test.

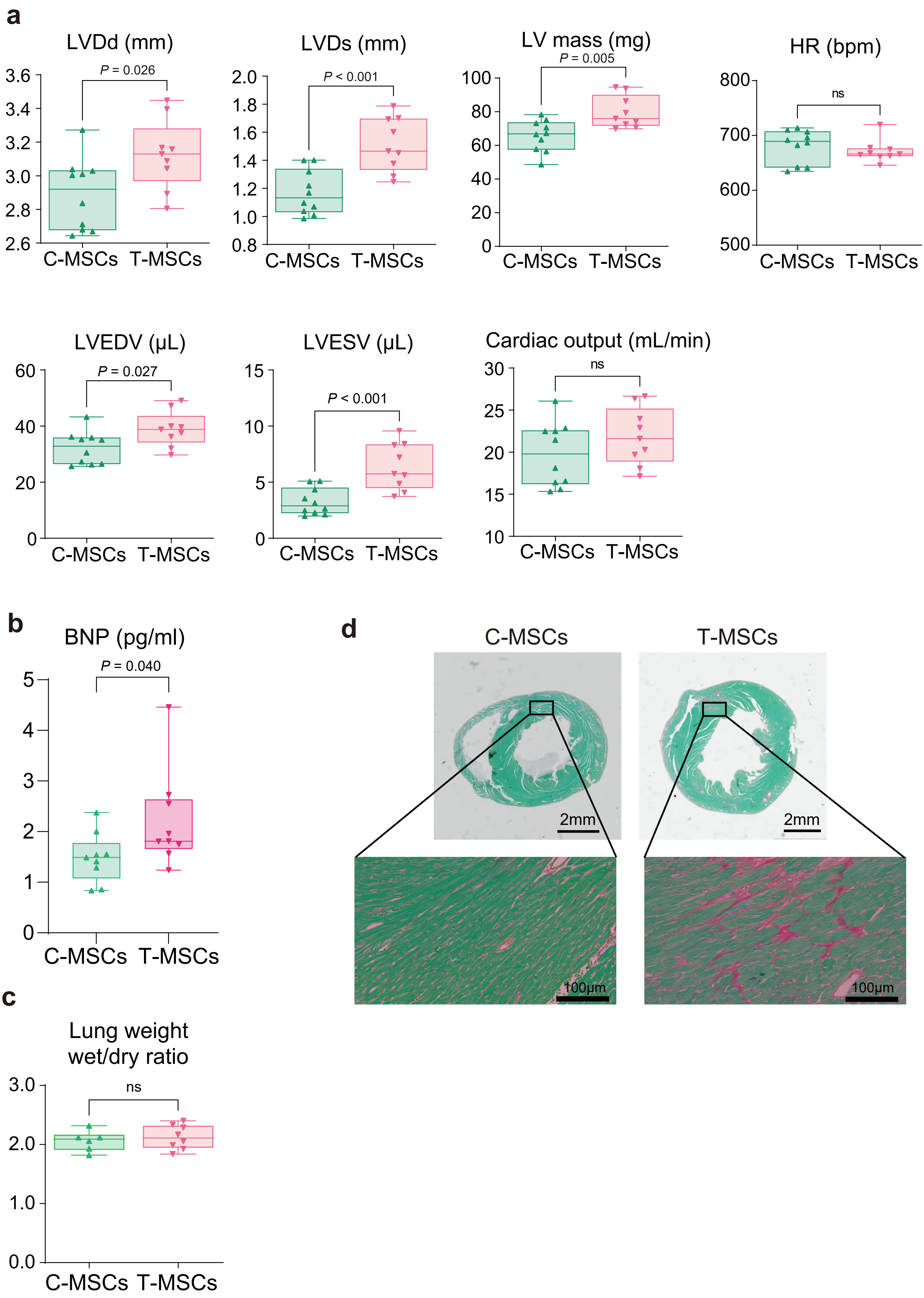

Supplementary Figure 3

Supplementary Figure 3. Cardiac function after co-transplantation of MSCs.

a. Echocardiographic assessment of recipient male mice 6 months after co-transplantation. LVEDV is shown in Fig. 1c. n = 10, 9 for C-MSC and T-MSC groups, respectively. Two-tailed unpaired Student's t-test.

b. Plasma BNP levels of recipient mice 6 months after co-transplantation. n = 9 in each group. Mann Whitney U-test.

c. Lung wet-to-dry weight ratios of recipient mice 6 months after co-transplantation. n = 6, 8 for C-MSC and T-MSC groups, respectively. Two-tailed unpaired Student's t-test.

d. Representative cardiac sections stained with Picrosirius Red/Fast Green; the collagen fibers are red, and the non-collagen proteins are green.

a

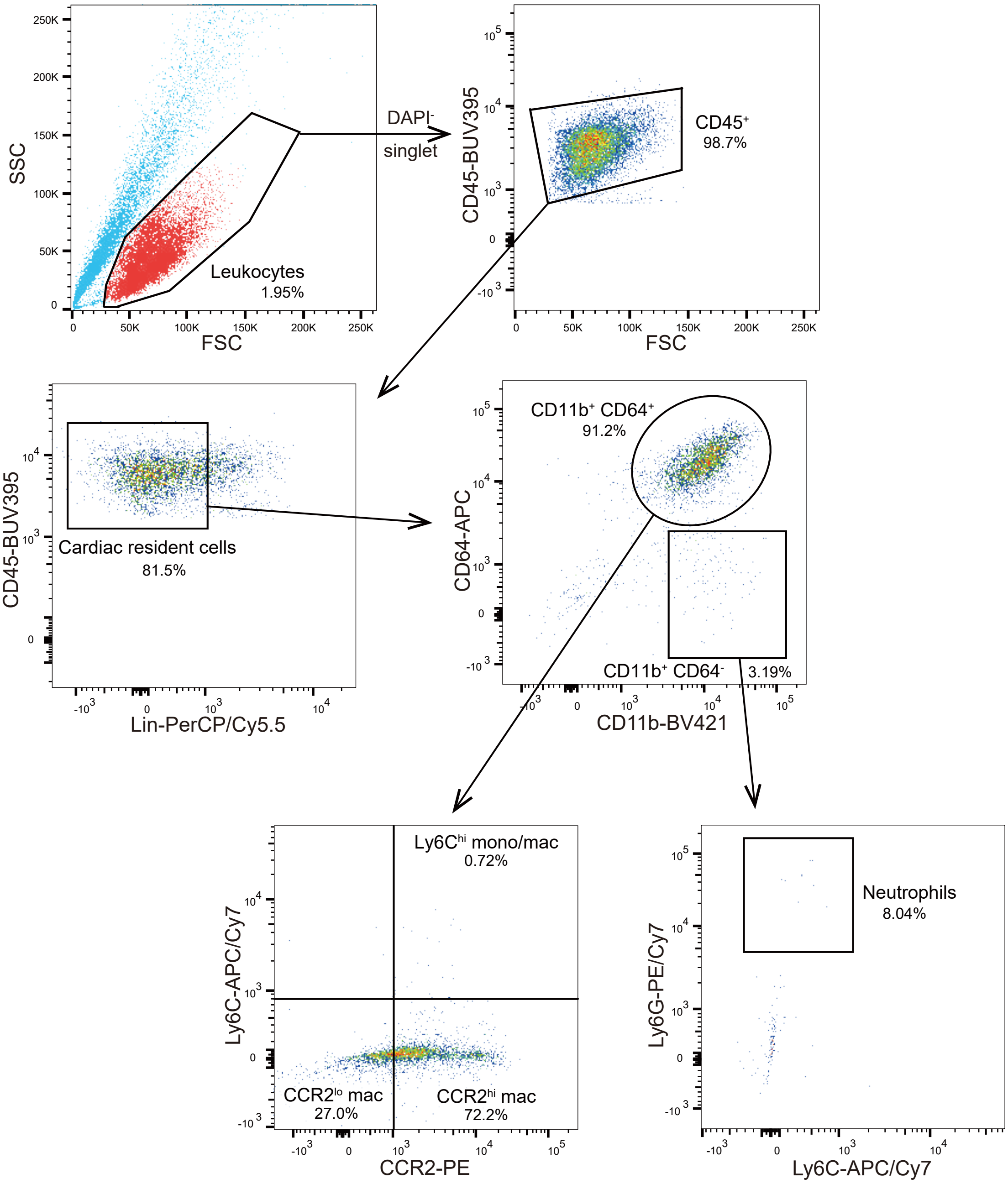

b

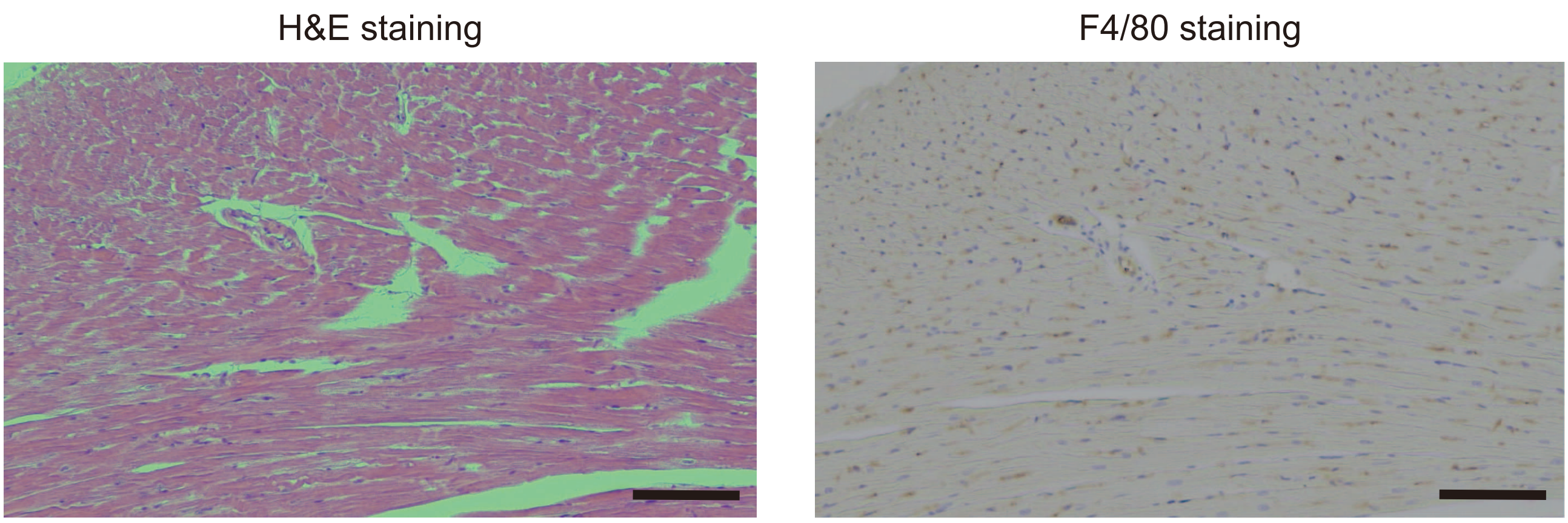

Supplementary Figure 4

Supplementary Figure 4. Gating strategy for flow cytometric analysis of cardiac macrophages.

a. After exclusion of doublets and dead cells, $CD45^+$ leukocytes were identified. Cells labeled by intravenously injected anti-CD45 antibodies (to exclude circulating leukocytes) were removed. $CD11b^+CD64^+$ cells were then gated as cardiac macrophages and further separated into $CCR2^{hi}$ and $CCR2^{lo}$ subsets. Plots corresponding to cardiac macrophages are highlighted in red in the initial FSC–SSC panel.

b. Hematoxylin and eosin (H&E) staining (left) and immunohistochemical staining for F4/80 (right) in left ventricular sections. F4/80 staining reveals abundant cardiac macrophages. Scale bars: 100 μm.

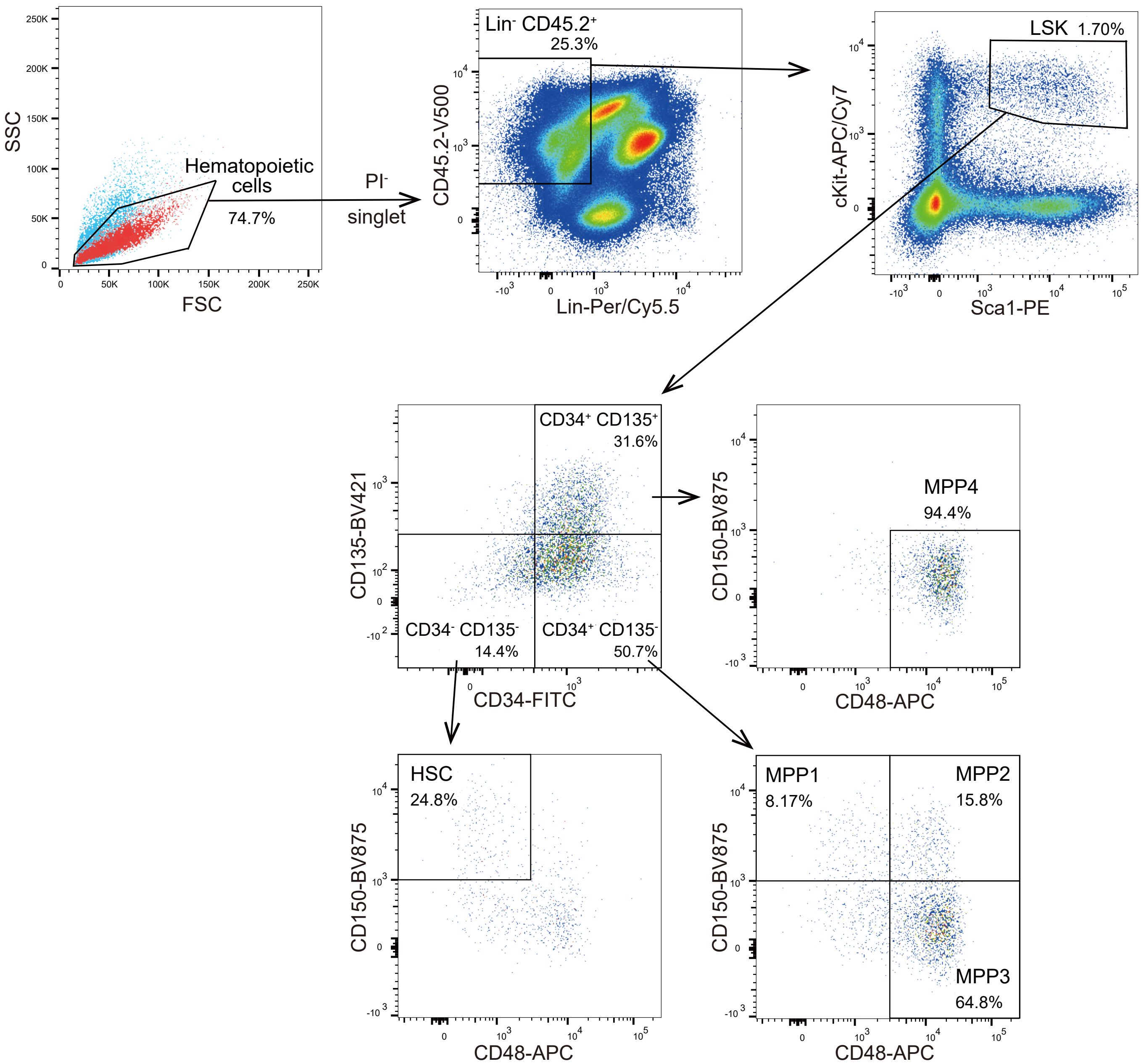

Supplementary Figure 5

Supplementary Figure 5. Gating strategy for flow cytometric analysis of HSPCs.

After exclusion of doublets, dead cells and hematopoietic lineage cells, we identified LSK cells, MPPs and LT-HSCs using described markers. CD4, CD8, CD11b, Ly6G, B220, CD127 and Ter119 were used for lineage markers. Plots corresponding to LSKs are highlighted in red in the initial FSC–SSC panel.

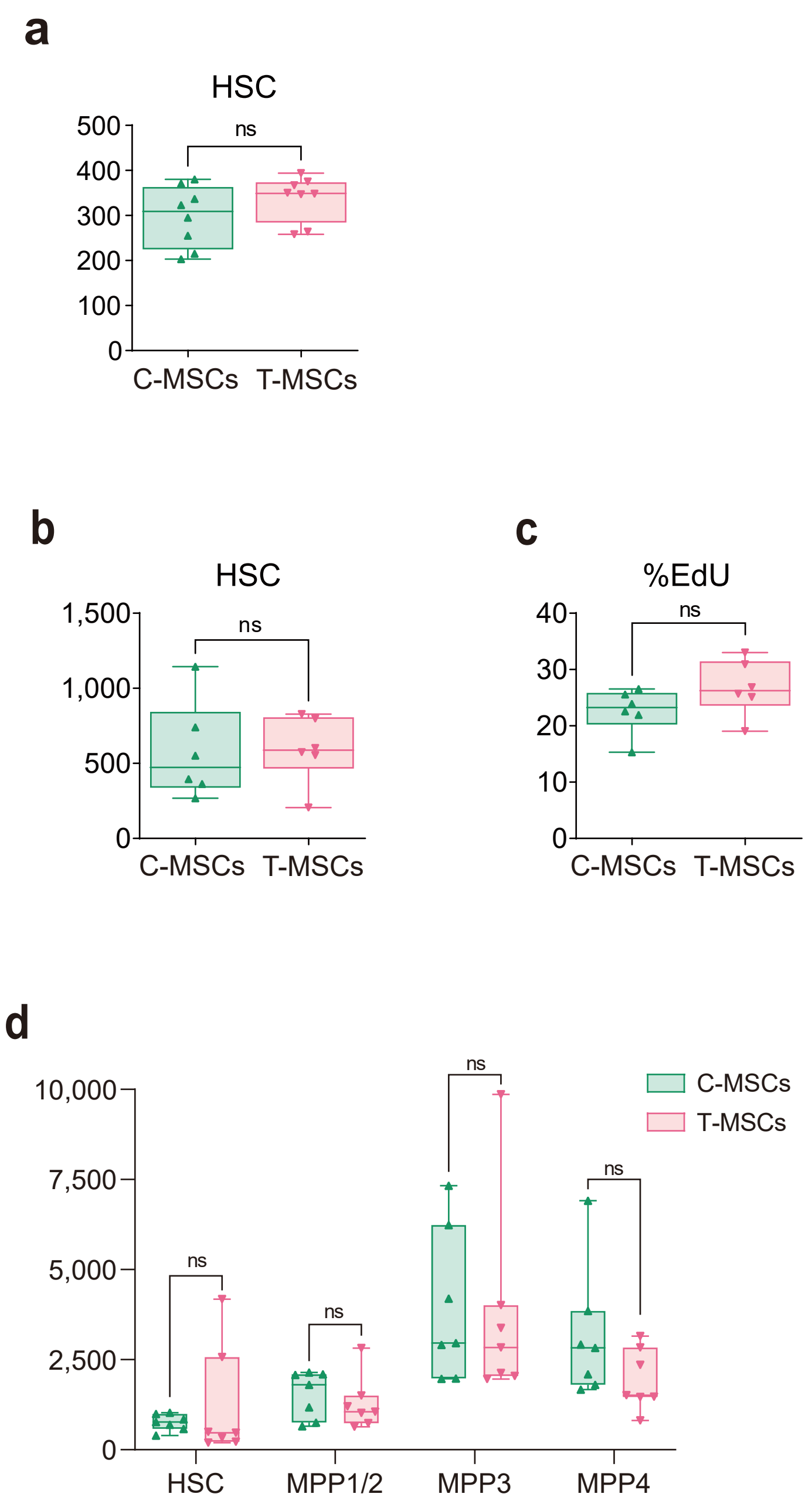

Supplementary Figure 6

Supplementary Figure 6. Early hematopoietic reconstitution after HSPC and MSC co-transplantation.

a. Numbers of $CD45.2^+$ HSCs in the bone marrow per femur 1 day after transplantation of $CD45.2^+$ donor cells together with MSCs into CD45.1 recipient mice. $n = 8$ per group.

b. Numbers of $CD45.2^+$ HSCs in the bone marrow per femur 1 week after transplantation. $n = 6$ per group.

c. Proportion of EdU^+ $CD45.2^+$ HSCs corresponding to the time point shown in panel b.

d. Numbers of $CD45.2^+$ HSPCs in the bone marrow per femur 1 month after transplantation. $n = 7$ per group. Two-tailed unpaired Student's *t*-tests were used.

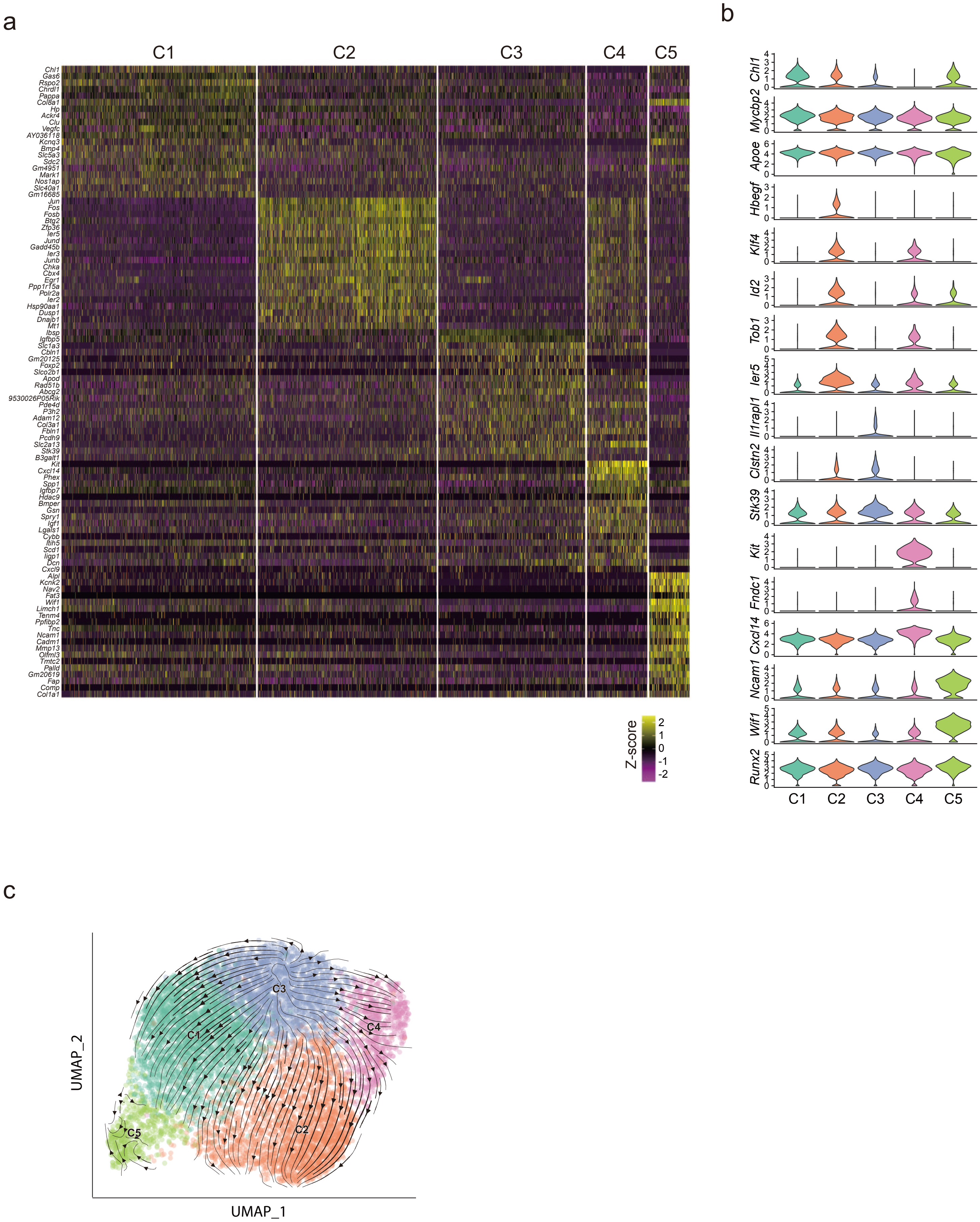

Supplementary Figure 7

Supplementary Figure 7. Clustering and characterization of MSC subpopulations in male mice.

a. Heatmap of representative marker genes for each cluster.

b. Violin plots of marker gene expression across clusters. Additional marker genes not shown in Fig. 2b are included.

c. RNA velocity vectors projected onto the UMAP embedding of MSCs.

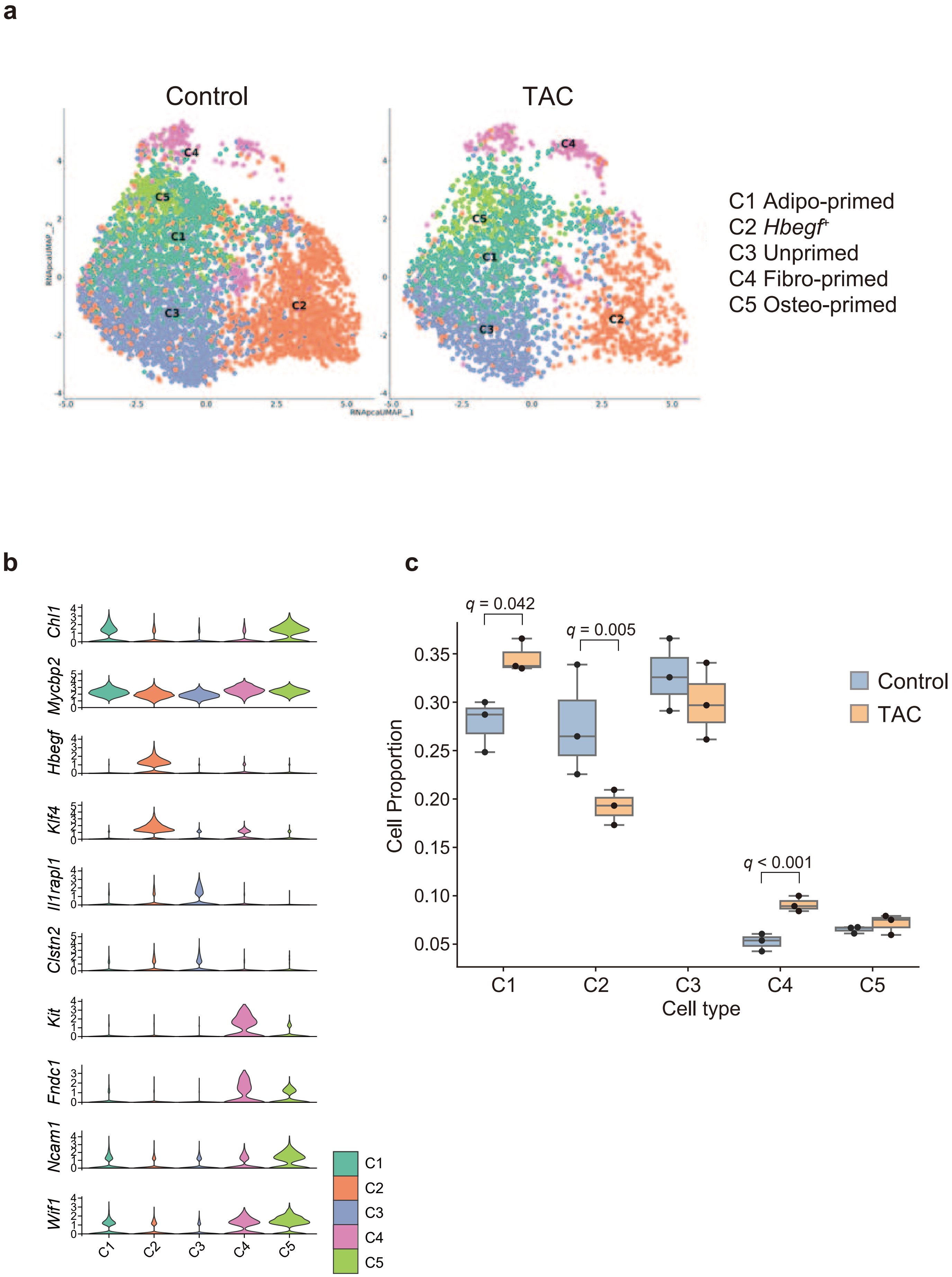

Supplementary Figure 8

Supplementary Figure 8. Clustering and characterization of MSC subpopulations in female mice.

a. UMAP plot of *Lepr*$^{+}$ MSCs from female mice (*n* = 3 control; *n* = 3 TAC) 4 weeks after surgery.

b. Marker gene expression across MSC clusters.

c. Proportions of cells within each MSC cluster in control and TAC groups. Differences in proportions were analyzed using beta regression, and *P* values were adjusted using the Benjamini-Hochberg method. Adjusted *P* values are shown only for significantly different comparisons. *n* = 3 female mice per group.

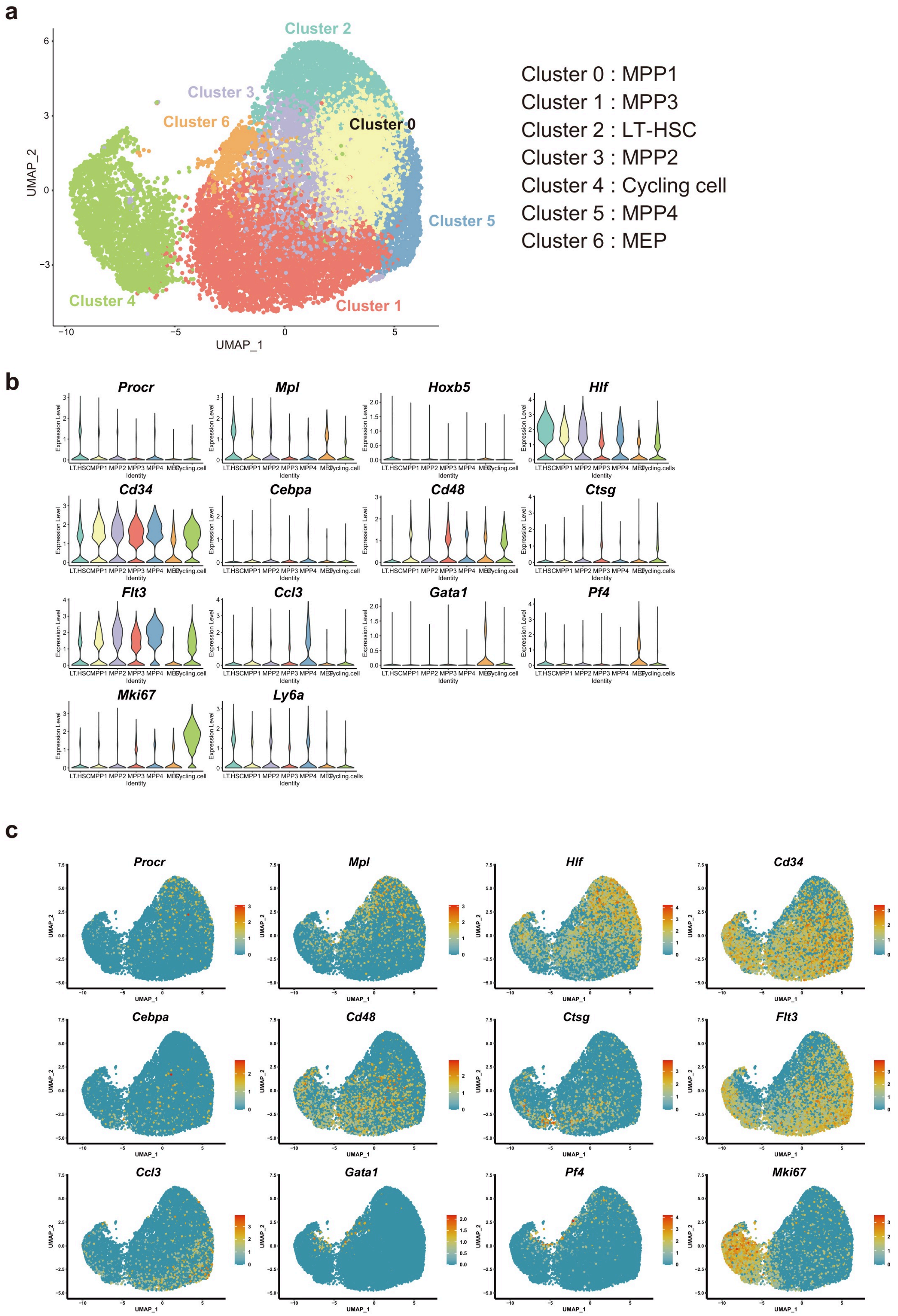

Supplementary Figure 9

Supplementary Figure 9. Clustering and identification of HSPC subpopulations

Clustering of control and post-TAC LSKs identified 7 clusters, as shown in **a**. Each cluster was assigned to a HSPC subpopulation based on expression of previously identified marker genes [26, 35, 36], as shown in violin plots (**b**) and UMAP visualization (**c**). The marker genes for cluster 6 were *Gata1* and *Pf4*, which are megakaryocyte/erythrocyte progenitor (MEP) specific genes. Cluster 4, which strongly expressed *Mki67*, was identified as cycling cells. Based on the high expression of marker genes for LT-HSCs (*Procr*, *Hlf*, *Mpl*) and the $Cd48^{-}Flt3^{-/\mathrm{low}}$ expression pattern, cluster 2 was identified as LT-HSCs. Expression of *Flt3* in Cluster 5 was abundant in MPP4. $Flt3^{\mathrm{low}}Cd48^{\mathrm{low}}Hlf^{\mathrm{int}}$ characteristics identified cluster 0 as MPP1 cells. Higher expression of *Cd48* identified clusters 1 and 3 as MPP2-3. Among them the differential expression of *Ctsg* discriminated clusters 1 and 3 and identified them as MPP3 and MPP2, respectively.

a

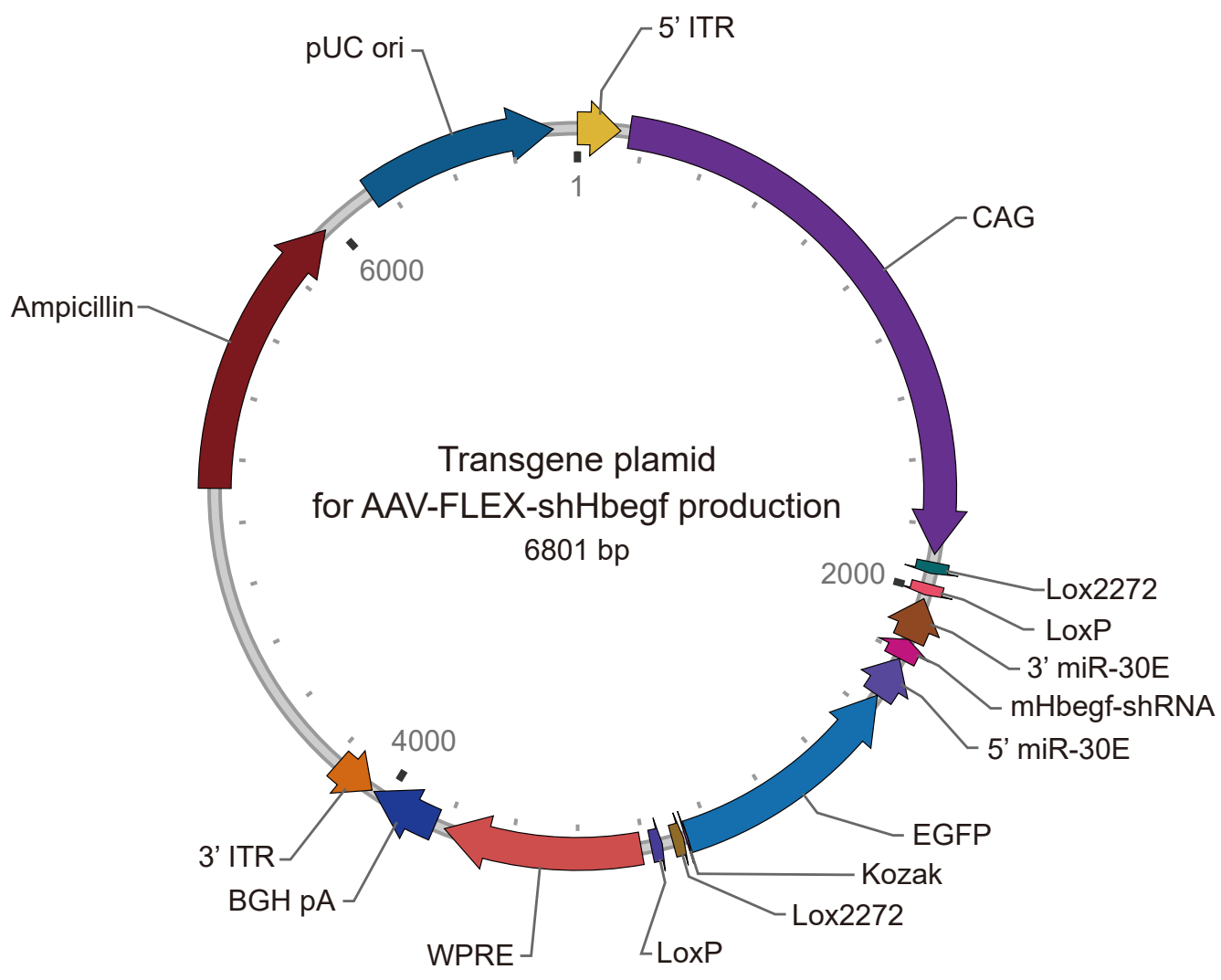

b

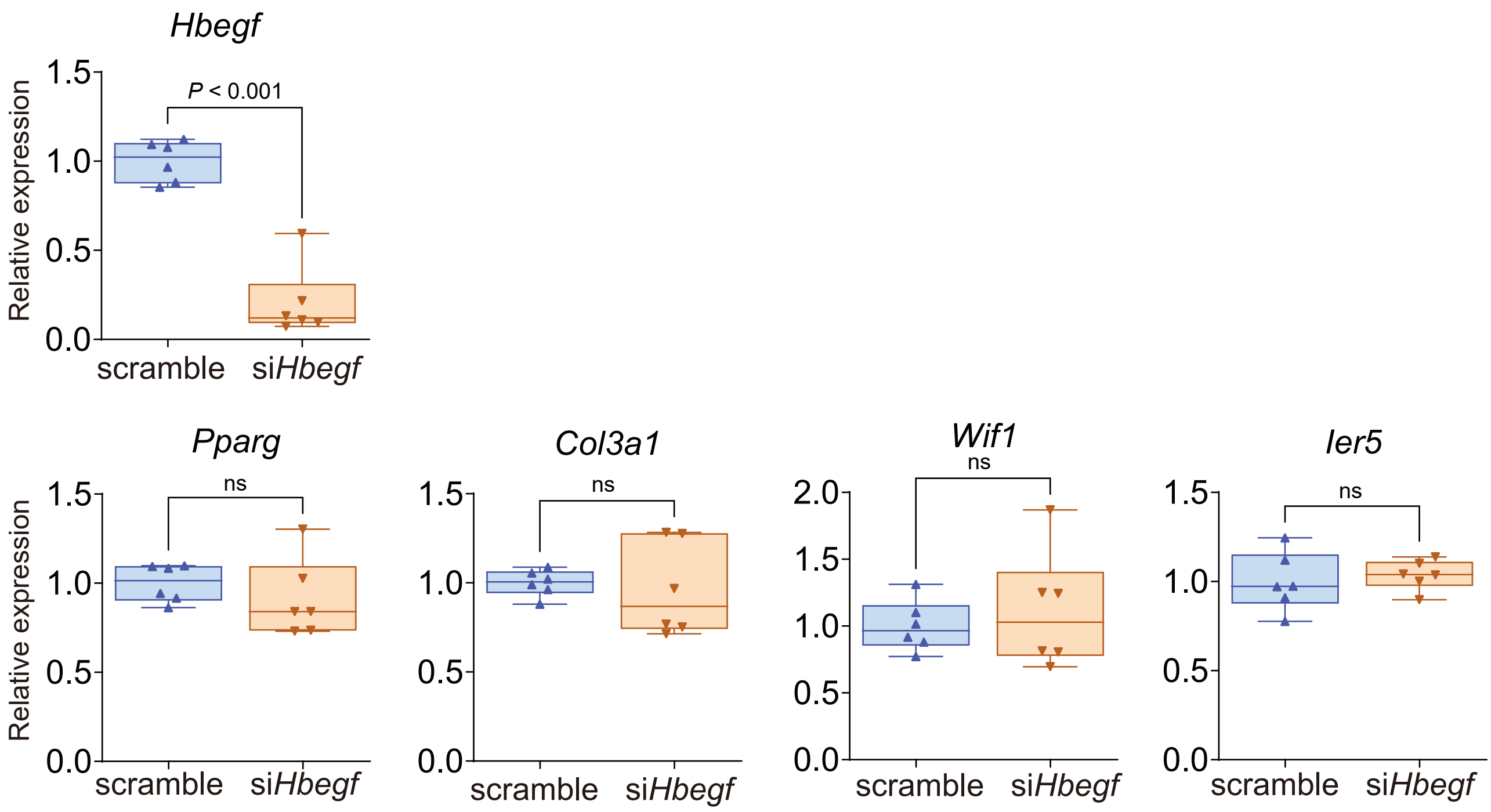

Supplementary Figure 10

Supplementary Figure 10. Effects of *Hbegf* inhibition on transcriptomes of MSCs

a. Vector map of AAV-FLEX-sh*Hbegf* is shown.

b. Expression of *Hbegf* and representative MSC subcluster signature genes in *ex vivo*–cultured primary MSCs following transfection with siRNA targeting *Hbegf* (siHbegf). Markers analyzed included *Pparg* (adipogenic, C1 cluster), *Col3a1* (fibrogenic, C4 cluster), *Wif1* (osteogenic, C5 cluster), and *Ier5* (immediate early gene enriched in the $Hbegf^{+}$ C2 cluster). $n = 6$ per group. Two-tailed unpaired Student's *t*-tests were used.

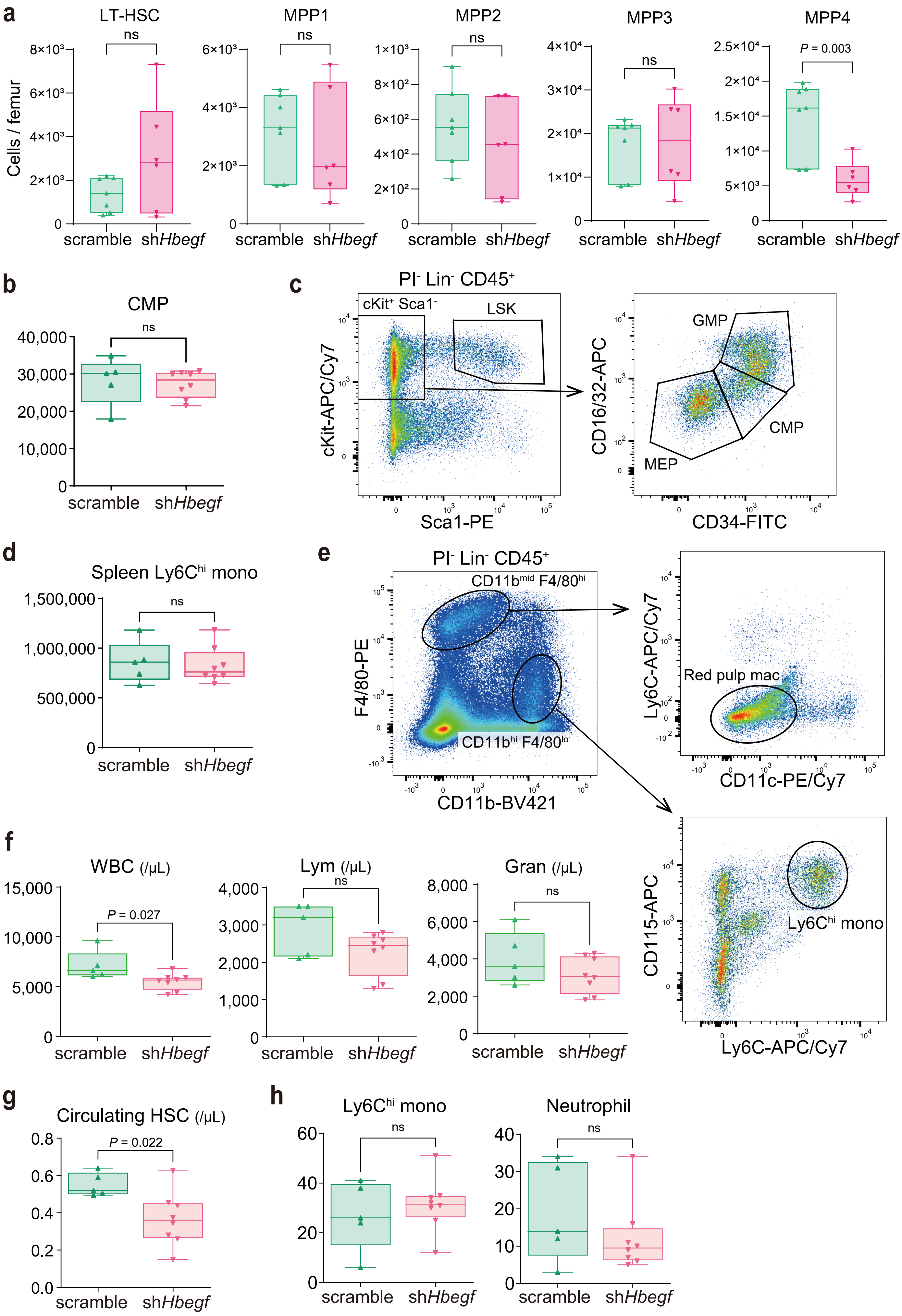

Supplementary Figure 11

Supplementary Figure 11. Effects of MSC-specific *Hbegf* repression on the hematopoietic system

a. Absolute numbers of HSPC subpopulations per femur, corresponding to the fractions shown in Fig. 3c.

b. Numbers of common myeloid progenitors (CMPs) per femur.

c. Gating strategy for flow cytometric analysis of CMP and GMP populations.

d. Number of $Ly6C^{hi}$ monocytes in the spleen.

e. Gating strategy for flow cytometric analysis of $Ly6C^{hi}$ monocytes in spleen.

f. Numbers of circulating leukocytes.

g. Numbers of circulating $CD150^{+}CD48^{-}$ HSCs in peripheral blood.

h. Numbers of $Ly6C^{hi}$ monocytes/macrophages and $Ly6G^{+}$ neutrophils in the left ventricle normalized to heart weight (cells per milligram of heart tissue).

Data in Supplementary Fig. 11a is from the same experiment as Fig. 3c (n = 7 for scramble, n = 6 for sh*Hbegf*). Data in Supplementary Figs. 11b-h were obtained from independent experiments corresponding to Fig. 3e and 3i (n = 5 for scramble, n = 8 for sh*Hbegf*). Statistical comparisons were performed using two-tailed unpaired Student's t-tests.

a

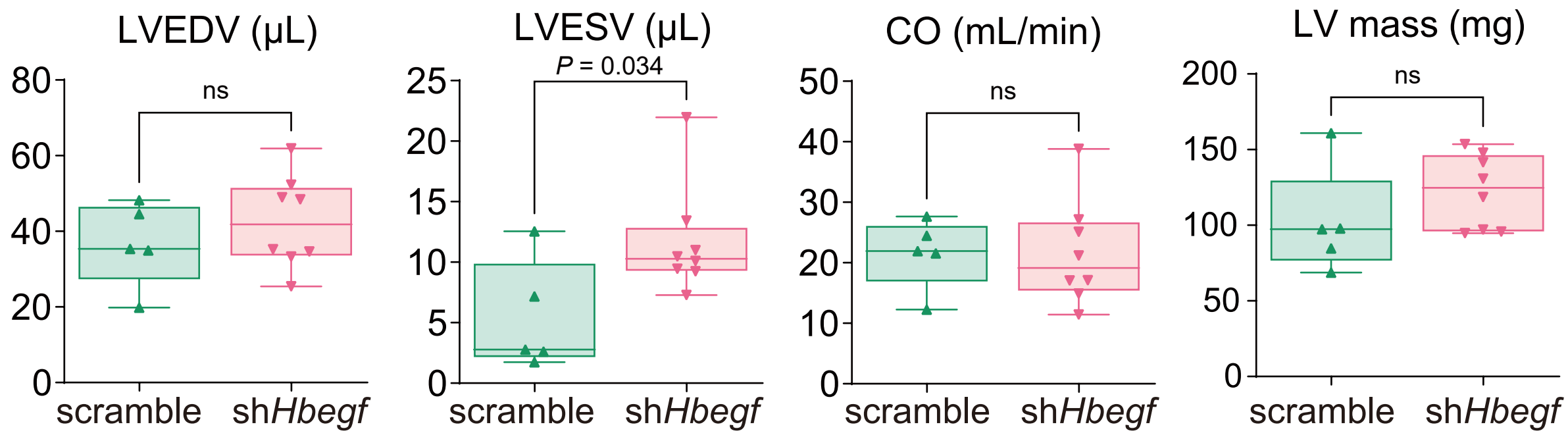

b

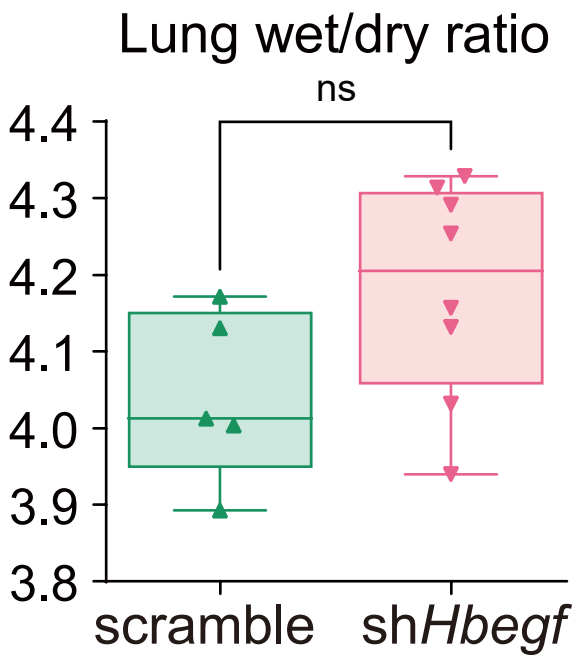

Supplementary Figure 12

Supplementary Figure 12. Effects of MSC-specific *Hbegf* repression on cardiac function.

a. Echocardiographic measurements of left ventricular ejection fraction, end-diastolic volume, end-systolic volume, cardiac output, and left ventricular mass.

b. Wet-to-dry lung weight ratios. Data were obtained from the same experiments as those used for Figs. 3e, 3f, and 3i (n = 5 scramble, n = 8 sh*Hbegf*). Statistical comparisons were performed using two-tailed unpaired Student's *t*-tests.

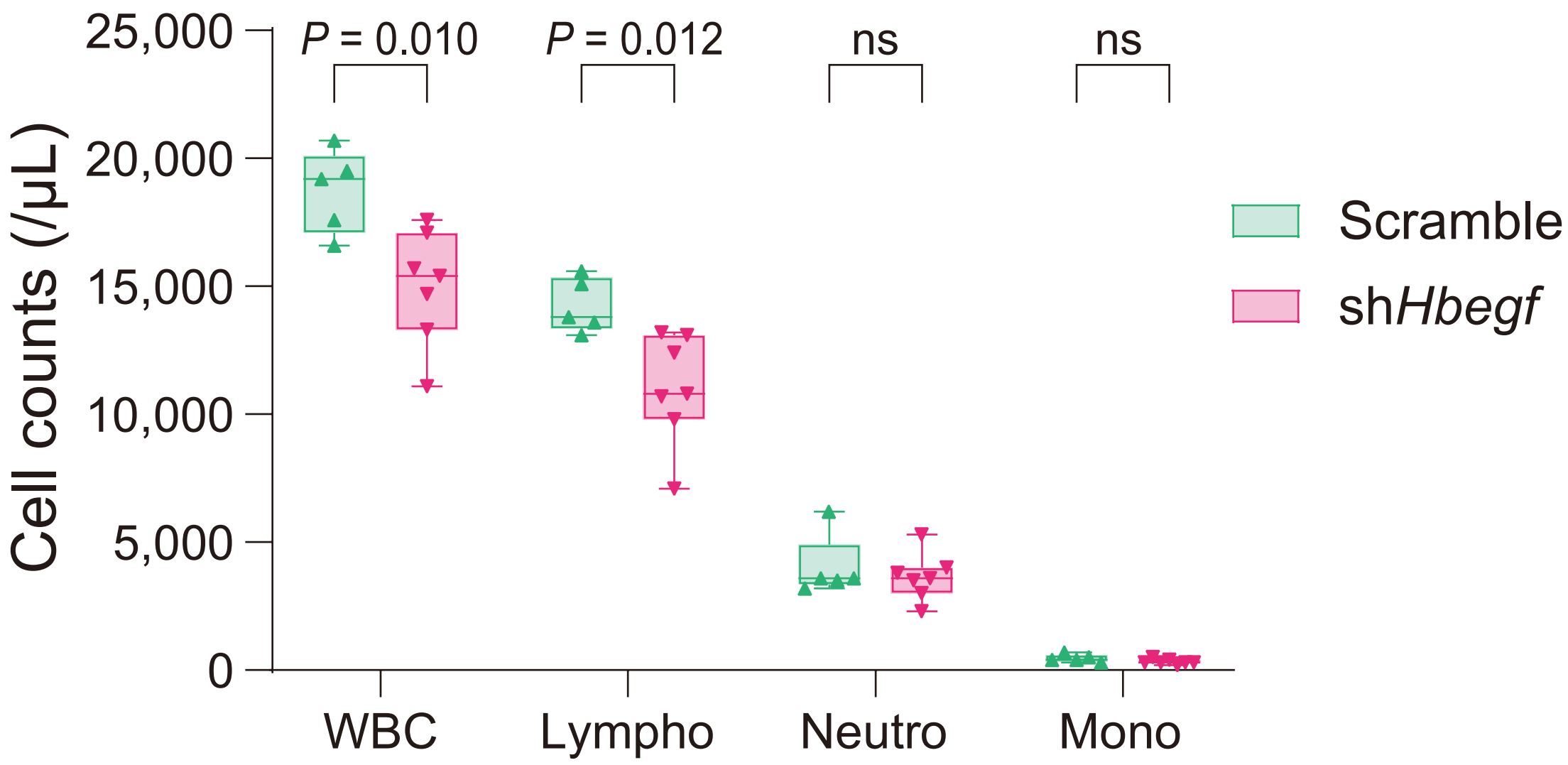

Supplementary Figure 13

Supplementary Figure 13. Effects of transplantation of *Hbegf*-repressed MSCs on the hematopoietic system.

Numbers of circulating leukocytes in recipients 3 months after transplantation of LSKs from male WT mice together with AAV-FLEX-scramble- or AAV-FLEX-sh*Hbegf*-transduced MSCs from male *Lepr*-Cre mice 2 weeks after AAV administration. *n* = 5 (scramble) and *n* = 7 (sh*Hbegf*). Two-tailed unpaired Student's *t*-test.

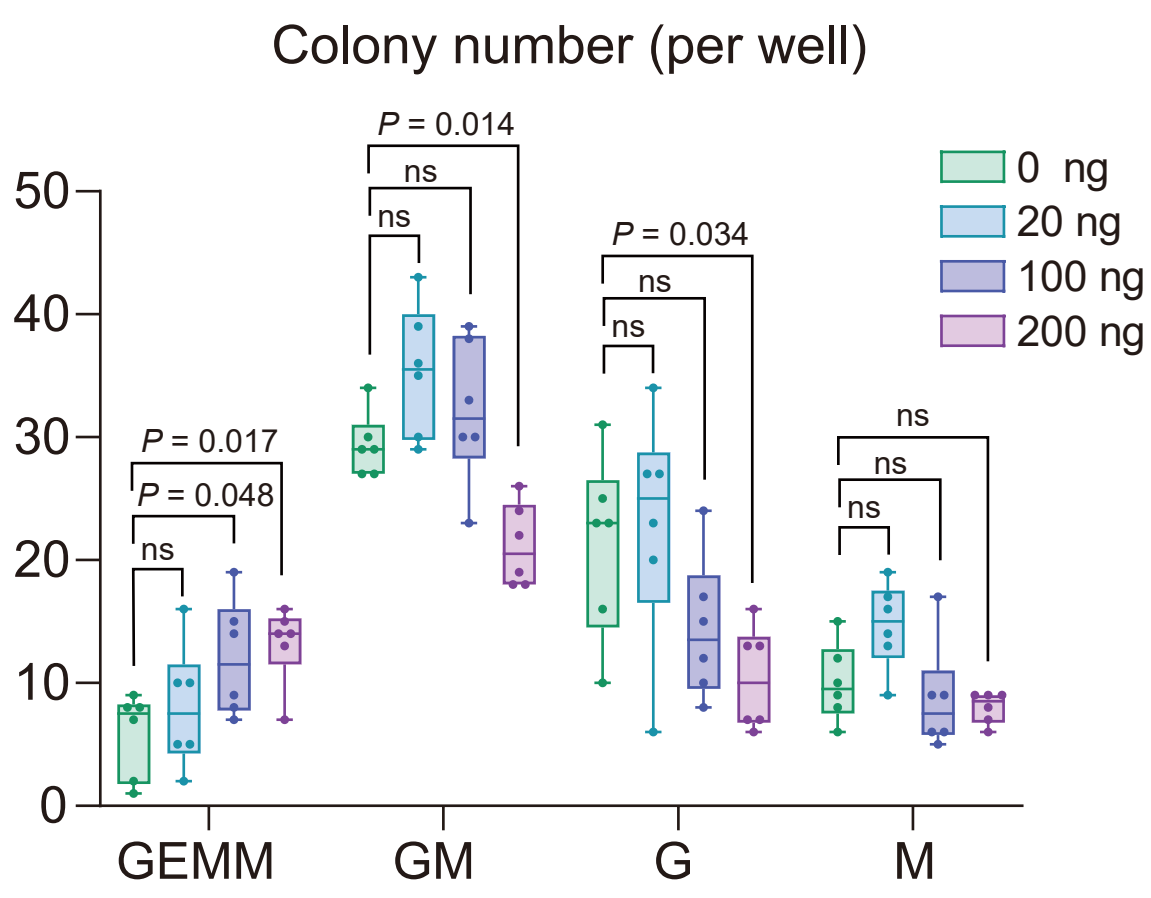

Supplementary Figure 14

Supplementary Figure 14. Effects of HBEGF on colony-forming activity of HSCs. Numbers of colony types generated in colony-forming unit assays. A total of 1,000 cultured HSCs were plated into each well of a 12-well plate and treated with vehicle or recombinant HBEGF (20 ng, 100 ng, or 200 ng per well). n = 6 wells per condition. Statistical comparisons with vehicle-treated wells were performed using ordinary one-way ANOVA followed by Dunnett's post-hoc tests.

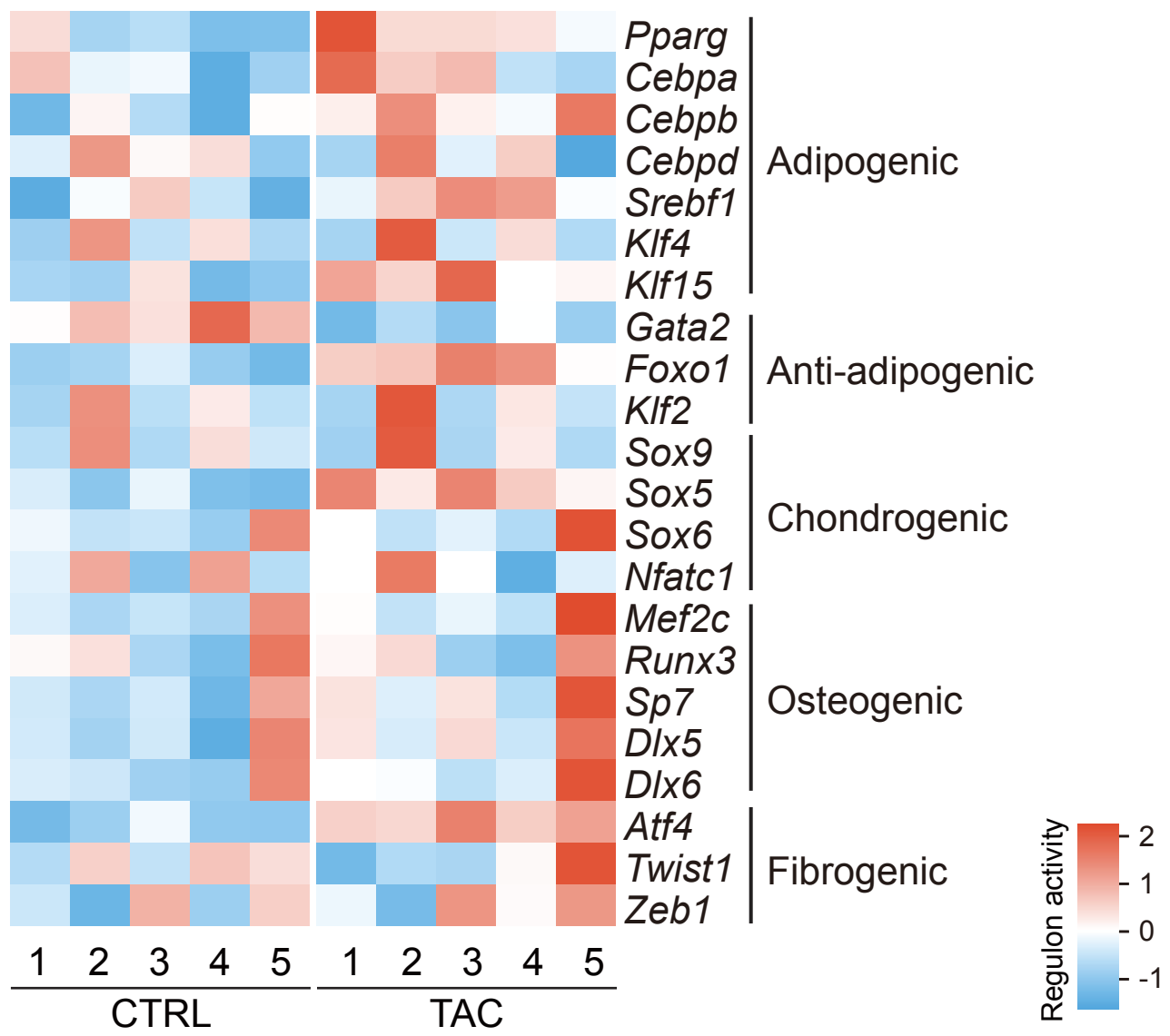

Supplementary Figure 15

Supplementary Figure 15. Regulon activity in MSC subpopulations.

Heatmap showing regulon activities of TFs known to be involved in the differentiation of MSCs.

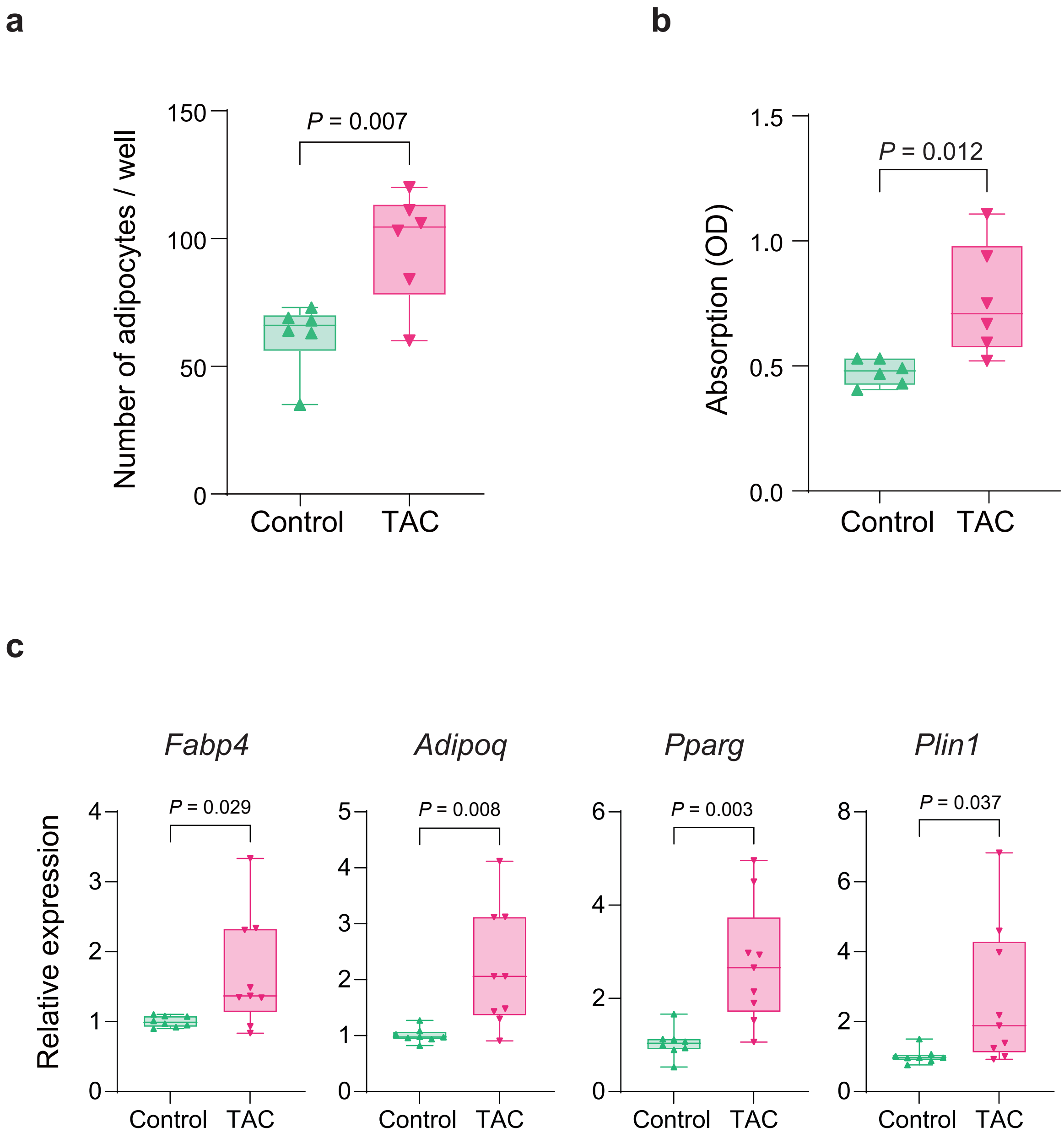

Supplementary Figure 16

Supplementary Figure 16. *In vitro* adipogenic differentiation of MSCs.

a. Numbers of Oil red O-positive adipocytes in MSC cultures derived from bone marrow cells of control and TAC male mice after 7 days of adipogenic induction. $n = 6$ per group. Two-tailed unpaired Student's t-test. Representative figures are shown in Fig. 4d.

b. Quantification of oil red O staining by optical absorbance at 530 nm. $n = 6$ per group. Two-tailed unpaired Student's t-test.

c. Expression of adipocyte marker genes (*Fabp4*, *Adipoq*, *Pparg*, *Plin1*) in MSCs from control or TAC male MSCs. $n = 8$ for control, $n = 9$ for TAC (from two independent experiments). Two-tailed unpaired Student's t-tests.

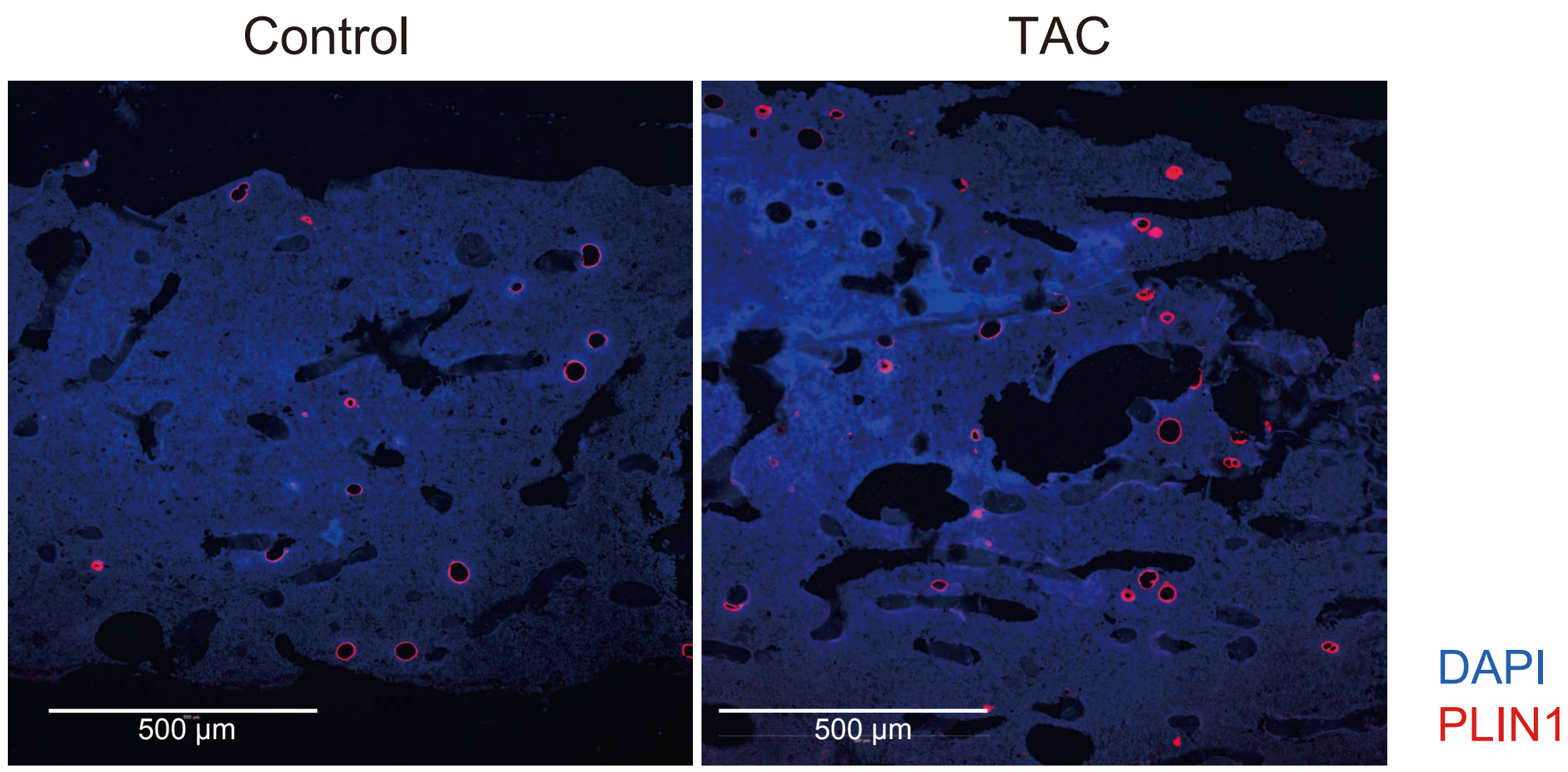

Supplementary Figure 17

Supplementary Figure 17. Adipogenesis in BM after TAC.

Representative immunofluorescence images of distal femurs from male mice 4 weeks after TAC and sham-operation. Nuclei are stained with 4',6-diamidino-2-phenylindole (DAPI), and adipocytes are labeled with perilipin-1 (PLIN1).

a

Mouse

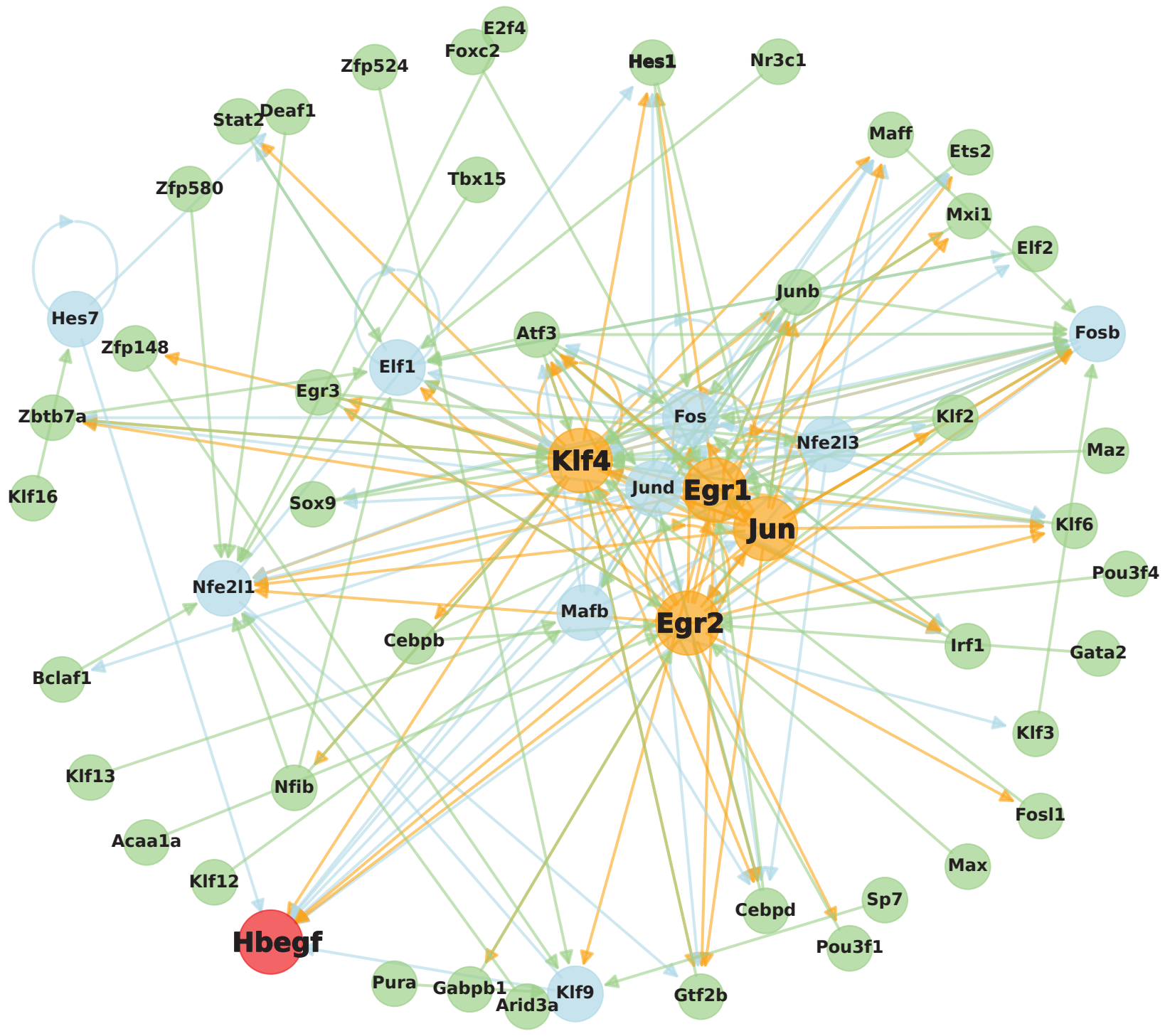

b

Human

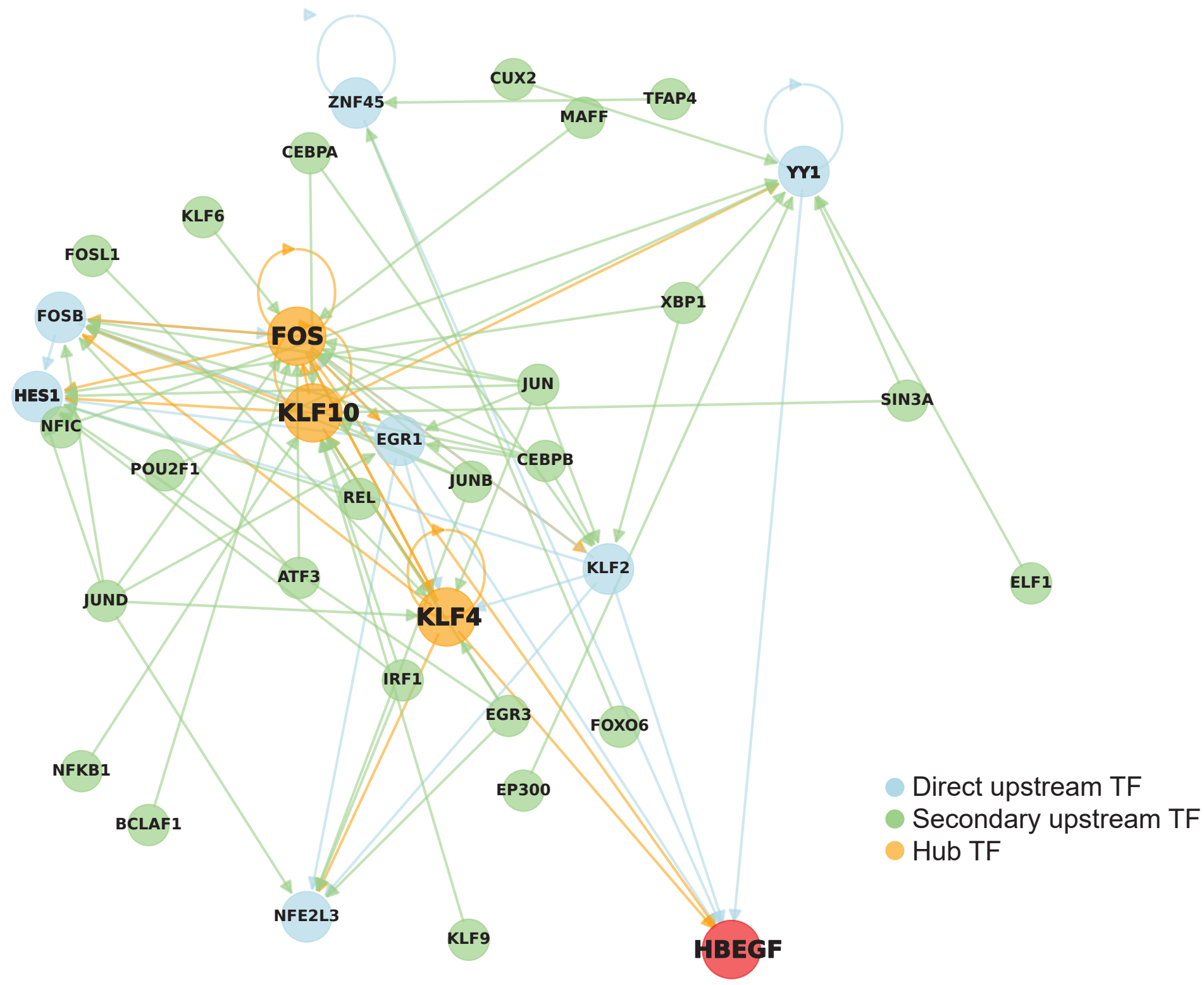

Supplementary Figure 18

Supplementary Figure 18. Transcriptional networks regulating HBEGF.

Transcriptional regulatory networks centered on HBEGF. SCENIC regulon data analyzed using mouse (a) and human (b) MSCs were used to construct the regulatory relationships among TFs involved in regulating HBEGF. The network was filtered using ChIP-Atlas binding data [92] to retain experimentally validated interactions. Orange nodes represent network hub TFs, the red node indicates HBEGF, and the light blue and light green nodes represent first-layer and second-layer upstream regulators, respectively. The edge width corresponds to the connection specificity index (CSI).

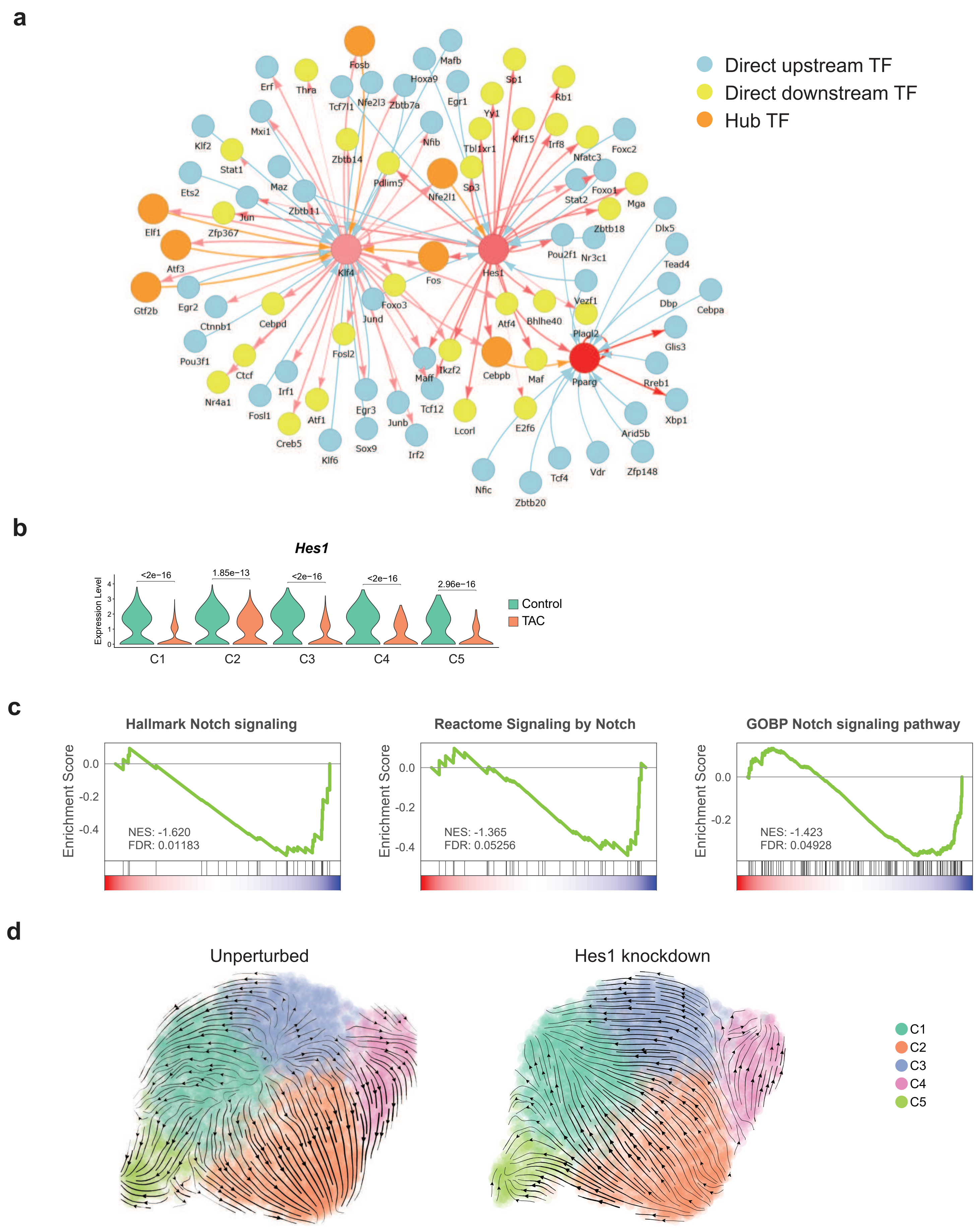

Supplementary Figure 19

Supplementary Figure 19. Regulatory network controlling MSC response to TAC.

a. SCENIC regulon analysis of mouse MSCs depicting regulatory relationships among TFs associated with KLF4 and PPARγ. The network was filtered using ChIP-Atlas binding data [92] to retain experimentally validated interactions. Orange nodes indicate hub TFs, the red node denotes PPARγ, and light blue and yellow nodes represent upstream and downstream regulators, respectively. Hes1 is positioned between KLF4 and PPARγ within the network.

b. Violin plots showing *Hes1* expression in MSC subpopulations. Wilcoxon rank-sum test with BH adjustment.

c. Gene set enrichment analysis (GSEA) of MSCs comparing TAC using a custom gene set collection comprising three MSigDB mouse Notch-related gene sets from Hallmark, Reactome, and GO biological process collections.

d. *In silico* perturbation analysis using Dynamo[53]. RNA velocity analysis identified differentiation trajectories from C3 cells into all other subsets, which is consistent with scVelo analysis (Supplementary Fig. 7c). *Hes1* knockdown shifted the vector field away from *Hbegf*$^{+}$ C2 cells toward adipogenic C1 cells.

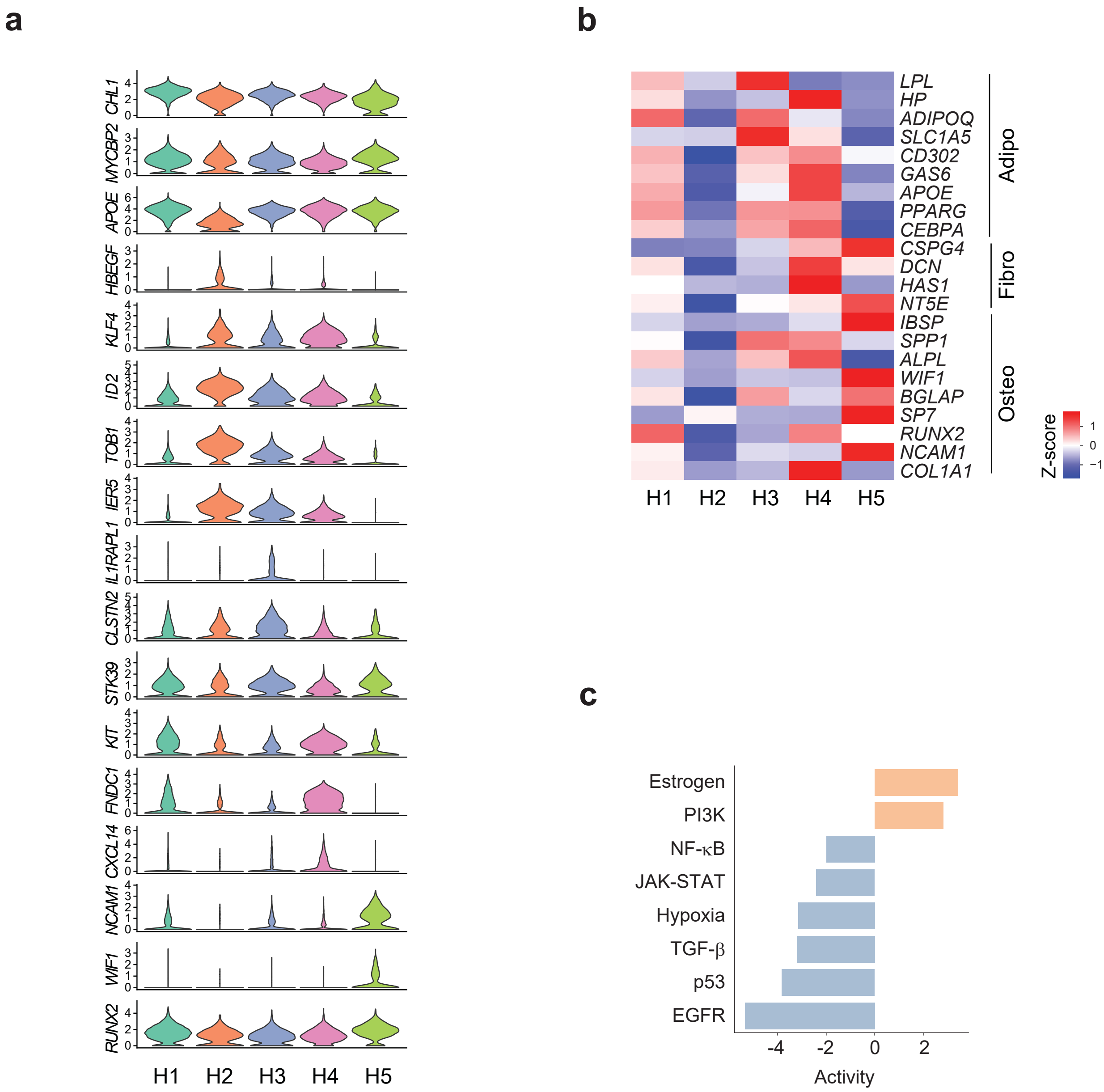

Supplementary Figure 20

Supplementary Figure 20. Human MSC and HSC analysis.

a. Violin plot of cluster marker genes in *LEPR*$^+$ MSCs.

b. Heatmap of expression of the known subpopulation marker genes in *LEPR*$^+$ MSCs.

c. PROGENy pathway enrichment analysis in HSCs from MI vs. control patients. A human BM HSPC dataset (E-MTAB-13509)[54] was analyzed for differentially expressed genes, and PROGENy pathway enrichment score was calculated. Positive scores indicate increased pathway activity in MI HSCs compared with controls. Top 8 pathways are shown.

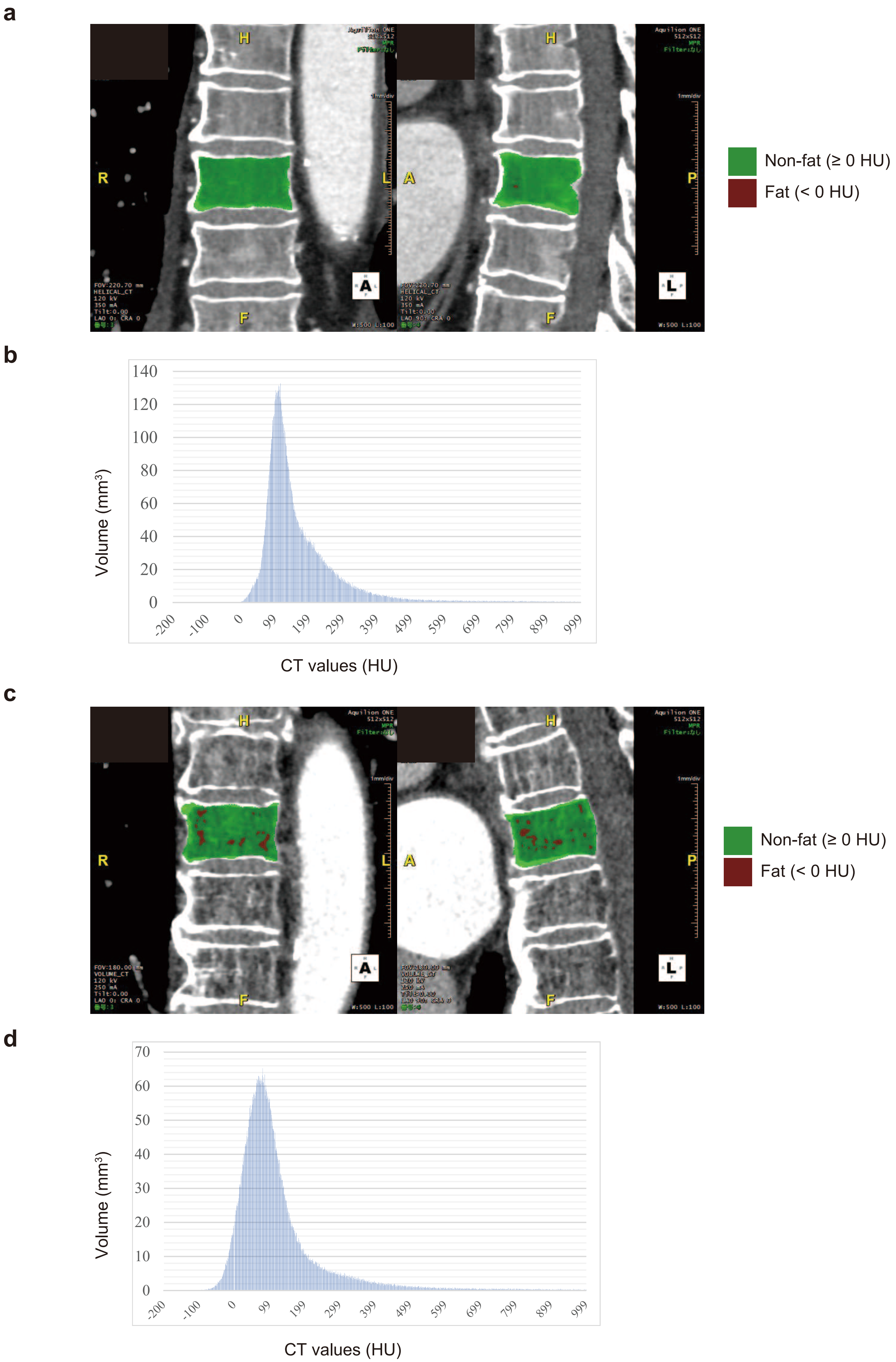

Supplementary Figure 21

Supplementary Figure 21. Quantitative analysis of bone marrow CT attenuation in human subjects.

a. Axial, coronal and sagittal views of the 7th thoracic vertebra (T7) in a healthy control subject.

b. Histogram of voxel volumes across CT attenuation values ranging from -200 to 1000 Hounsfield units (HU) in the BM of T7 from a healthy control.

c. Axial, coronal and sagittal views of T7 in a patient with HF.

d. Histogram of voxel volumes across CT attenuation values ranging from -200 to 1000 HU in the BM of T7 from a patient with HF.

In a and c, bone marrow regions with CT attenuation below 0 HU, corresponding to fat, are shown in red, and regions with attenuation at or above 0 HU are shown in green.

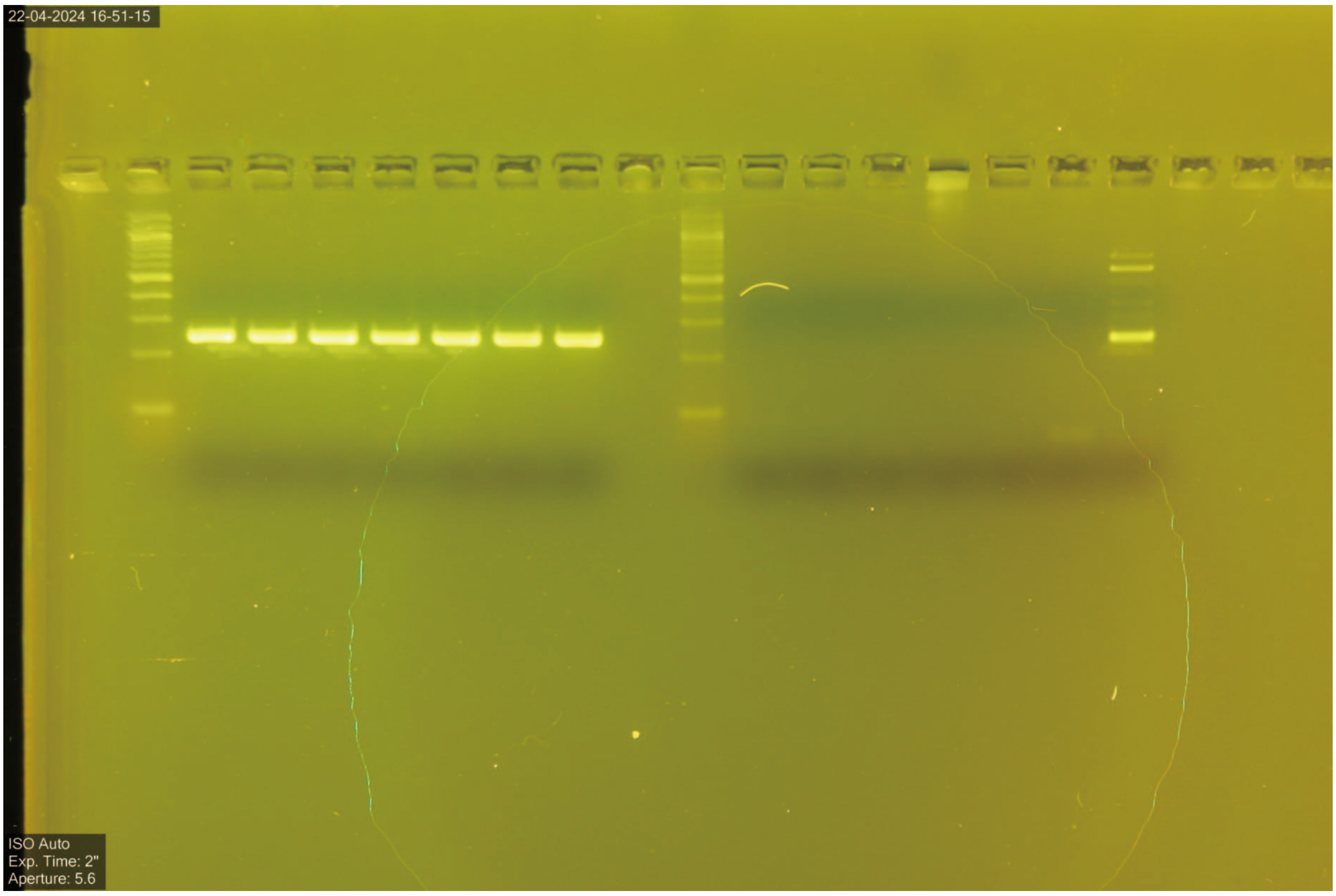

Supplementary Figure 22

Supplementary Figure 22. Uncropped gel image for Supplementary Fig. 2a.

Full, uncropped agarose gel image corresponding to the RT-PCR analysis shown in Supplementary Fig. 2a. cDNA was prepared from the liver, lung, kidney, spleen, heart, muscle and bone marrow of a recipient of $cKit^+$ bone marrow cells and C-MSCs, and the amplified products were resolved on a single agarose gel.

Left, 18S ribosomal RNA (internal control); right, tdTomato. Within each set, the lanes from left to right are liver, lung, kidney, spleen, heart, muscle and bone marrow. The regions shown in Supplementary Fig. 2a are indicated by the boxes/dashed outlines. The gel was run once.
