# Supplementary material for: Altered bone marrow niche forms central innate immune memory driving cardiac dysfunction
Source: Nat Commun. 2026 Aug 26;17:8261. doi: 10.1038/s41467-026-76178-z (PMC13518785; doi:10.1038/s41467-026-76178-z)
Supplement: Supplementary file 2 [file 41467_2026_76178_MOESM2_ESM.pdf]

nature portfolio

Corresponding author(s): Katsuhito Fujiu

Last updated by author(s): Mar 24, 2026

Reporting Summary

Nature Portfolio wishes to improve the reproducibility of the work that we publish. This form provides structure for consistency and transparency in reporting. For further information on Nature Portfolio policies, see our Editorial Policies and the Editorial Policy Checklist.

Statistics

For all statistical analyses, confirm that the following items are present in the figure legend, table legend, main text, or Methods section.

n/a	Confirmed	
☐	☒	The exact sample size (*n*) for each experimental group/condition, given as a discrete number and unit of measurement
☐	☒	A statement on whether measurements were taken from distinct samples or whether the same sample was measured repeatedly
☐	☒	The statistical test(s) used AND whether they are one- or two-sided<br>*Only common tests should be described solely by name; describe more complex techniques in the Methods section.*
☐	☒	A description of all covariates tested
☐	☒	A description of any assumptions or corrections, such as tests of normality and adjustment for multiple comparisons
☐	☒	A full description of the statistical parameters including central tendency (e.g. means) or other basic estimates (e.g. regression coefficient) AND variation (e.g. standard deviation) or associated estimates of uncertainty (e.g. confidence intervals)
☐	☒	For null hypothesis testing, the test statistic (e.g. *F*, *t*, *r*) with confidence intervals, effect sizes, degrees of freedom and *P* value noted<br>*Give P values as exact values whenever suitable.*
☒	☐	For Bayesian analysis, information on the choice of priors and Markov chain Monte Carlo settings
☒	☐	For hierarchical and complex designs, identification of the appropriate level for tests and full reporting of outcomes
☒	☐	Estimates of effect sizes (e.g. Cohen's *d*, Pearson's *r*), indicating how they were calculated

Our web collection on statistics for biologists contains articles on many of the points above.

Software and code

Policy information about availability of computer code

Data collection	Data were collected using the following software and platforms:<br>FACSDiva (BD Biosciences) for flow cytometry data acquisition, Cell Ranger (10x Genomics) for initial processing of single-cell RNA sequencing data, Vevo 3100 imaging system software (FUJIFILM VisualSonics) for echocardiographic measurements, LightCycler 480 software (Roche) for quantitative real-time PCR data acquisition, and instrument-specific software associated with microscopy systems (Olympus FV3000, Olympus APX100, Nikon microscopy) for image acquisition.
Data analysis	Data analysis was performed using the following software and computational tools:<br>FlowJo (BD Biosciences) for flow cytometry analysis, GraphPad Prism 9 (GraphPad Software) for statistical analysis and data visualization;<br>R and Python-based packages for statistical and computational analyses, Seurat version 4 and 5, in part through a modified version of SCALA (SCALAplus; https://github.com/Manabe-lab/SCALAplus, archived at https://doi.org/10.5281/zenodo.20566705) for single-cell RNA sequencing data processing and clustering, Cell Ranger (10x Genomics) for read alignment and gene quantification, fastMNN for batch correction, MAST, edgeR, and DESeq2 for differential expression analyses, NicheNet for ligand–receptor interaction analysis, scVelo and velocyto for RNA velocity analysis, Dynamo for in silico perturbation analysis, pySCENIC for gene regulatory network inference, decoupler (Python implementation) for pathway activity inference including PROGENy, muscat for pseudobulk analysis, statsmodels (Python) for beta regression analysis; network and pseudobulk analyses were additionally performed using the METIS pipeline (https://github.com/Manabe-lab/metis, archived at https://doi.org/10.5281/zenodo.20566713);<br>Intuition software (TeraRecon) for data acquisition and quantification from computed tomography ;<br>ImageJ (NIH) for histological image quantification, and Adobe Illustrator, Microsoft Excel, and BioRender for figure preparation.

For manuscripts utilizing custom algorithms or software that are central to the research but not yet described in published literature, software must be made available to editors and reviewers. We strongly encourage code deposition in a community repository (e.g. GitHub). See the Nature Portfolio guidelines for submitting code & software for further information.

Data

Policy information about availability of data

All manuscripts must include a data availability statement. This statement should provide the following information, where applicable:

- Accession codes, unique identifiers, or web links for publicly available datasets
- A description of any restrictions on data availability
- For clinical datasets or third party data, please ensure that the statement adheres to our policy

The sequencing data generated in this study have been deposited in the Gene Expression Omnibus (GEO) under accession number GSE326308 (bulk RNA-seq of murine MSCs), GSE326120 (scRNA-seq of male murine HSPCs and MSCs) and GSE326121 (scRNA-seq of female murine MSCs).
Publicly available datasets used in this study include GSE253355 (human bone marrow niche scRNA-seq) and E-MTAB-13509 (human bone marrow HSPC scRNA-seq). Source data for all figures are provided with this paper.
Clinical imaging data used in this study are not publicly available due to institutional and ethical restrictions but are available from the corresponding author upon reasonable request and with permission of the institutional review board.

Research involving human participants, their data, or biological material

Policy information about studies with human participants or human data. See also policy information about sex, gender (identity/presentation), and sexual orientation and race, ethnicity and racism.

Reporting on sex and gender	This study included a retrospective cohort of patients who underwent coronary computed tomography at the University of Tokyo Hospital. The study protocol was approved by the institutional review board (approval no. 2650-14), and the requirement for informed consent was waived due to the retrospective nature of the study. Patient characteristics, including age and sex, are summarized in Table 1 and Table 2. Age- and sex-matched controls were included for comparison. Sex was recorded based on clinical records. Both male and female participants were included, and analyses were not stratified by sex due to the limited sample size.
Reporting on race, ethnicity, or other socially relevant groupings	All participants were recruited from a single institution in Japan and were of Japanese ethnicity. No analyses were performed based on race or ethnicity.
Population characteristics	The study included 257 patients who underwent coronary computed tomography between January 2024 and August 2024 at a single institution in Japan. Among these, 15 patients with a clinical diagnosis of heart failure were identified based on medical records. Age- and sex-matched healthy controls were included at a 2:1 ratio. Patient characteristics, including age and sex distribution, are summarized in Table 1 and Table 2. All participants were of Japanese ethnicity, reflecting the population served by the institution. Analyses were not stratified by sex due to the limited sample size.
Recruitment	Participants were retrospectively identified from clinical records of patients who underwent coronary computed tomography at the University of Tokyo Hospital during the study period. Patients with heart failure were identified based on electronic medical records, and control participants were selected to match age and sex. No prospective recruitment or intervention was performed.
Ethics oversight	This study was approved by the Research Ethics Committee of the Graduate School of Medicine, the University of Tokyo (approval no. 2650-14). The requirement for informed consent was waived due to the retrospective nature of the study and the use of anonymized clinical data.

Note that full information on the approval of the study protocol must also be provided in the manuscript.

Field-specific reporting

Please select the one below that is the best fit for your research. If you are not sure, read the appropriate sections before making your selection.

[x] Life sciences [ ] Behavioural & social sciences [ ] Ecological, evolutionary & environmental sciences

For a reference copy of the document with all sections, see nature.com/documents/nr-reporting-summary-flat.pdf

Life sciences study design

All studies must disclose on these points even when the disclosure is negative.

Sample size	Sample sizes were determined based on the type of measurements performed and prior experience with variability in each experimental system. No formal power calculations were performed. The sample sizes used are consistent with those reported in previous studies in the field and were sufficient to detect statistically significant differences. Statistical analyses were performed using GraphPad Prism (version 9).
Data exclusions	No data were excluded from the analyses unless technical issues occurred (e.g., inadequate sample quality). Any exclusion criteria were applied consistently across all experimental groups.
Replication	All experiments were performed with independent biological replicates, as indicated in the figure legends. Key findings were confirmed in at least two independent experiments.

Randomization	Animals were randomly assigned to experimental groups.
Blinding	Investigators were not blinded to group allocation during experiments. However, outcome assessments, including echocardiographic measurements, histological analyses, and flow cytometry data analysis, were performed by independent investigators using standardized procedures.

Reporting for specific materials, systems and methods

We require information from authors about some types of materials, experimental systems and methods used in many studies. Here, indicate whether each material, system or method listed is relevant to your study. If you are not sure if a list item applies to your research, read the appropriate section before selecting a response.

Materials & experimental systems

n/a	Involved in the study
☐	☒ Antibodies
☐	☒ Eukaryotic cell lines
☒	☐ Palaeontology and archaeology
☐	☒ Animals and other organisms
☐	☒ Clinical data
☒	☐ Dual use research of concern
☒	☐ Plants

Methods

n/a	Involved in the study
☒	☐ ChIP-seq
☐	☒ Flow cytometry
☒	☐ MRI-based neuroimaging

Antibodies

Antibodies used	For FACS analysis, Ter119-PerCP/Cy5.5 (clone TER-119, 116228, BioLegend), CD45-PerCP/Cy5.5 (clone 30-F11, 103132, BioLegend), CD45-FITC (clone 30-F11, 103108, BioLegend), CD45.2-V500 (clone 104, 562130, BD Biosciences), CD45.2-BUV395 (clone 104, 564616, BD), CD45.1-BV711 (clone A20, 110739, BioLegend), CD71-BV421 (clone R17217, 113813, BioLegend), CD144-APC (clone BV13, 1380112, BioLegend), CD31-PE/Cy7 (clone 390, 102418, BioLegend), CD41-FITC (clone MWReg30, 133904, BioLegend), CD61-FITC (clone 2C9.G2, 104306, BioLegend), CD11b-FITC (clone M1/70, 101206, BioLegend), CD11b-BV421 (clone M1/70, 101236, BioLegend), CD11b-PerCP/Cy5.5 (clone M1/70, 101228, BioLegend), Ly6G-PE/Cy7 (clone 1A8, 127618, BioLegend), Ly6G-PerCP/Cy5.5 (clone 1A8, 127616, BioLegend), Ly6C-APC/Cy7 (clone HK1.4, 128026, BioLegend), Gr1-FITC (clone RB6-8C5, 108406, BioLegend), CD45R-FITC (clone RA3-6B2, 103206, BioLegend), CD5-FITC (clone 53-7.3, 100606, BioLegend), Leptin R Biotinylated Antibody (BAF497, R&D), Sca1-PE (clone D7, 108108, BioLegend), cKit-APC/Cy7 (clone 2B8, 105814, BioLegend), CD34-FITC (clone RAM34, 553733, BD Biosciences), CD135-BV421 (clone A2F10, 135313, BioLegend), CD48-PE/Cy7 (clone HM48-1, 103424, BioLegend), CD150-BV785 (clone TC15-12F12.2, 115937, BioLegend), CD16/32-APC (clone 93, 101326, BioLegend), CD4-PerCP/Cy5.5 (clone GK1.5, 100433, BioLegend), CD8a-PerCP/Cy5.5 (clone 53-6.7, 100734, BioLegend), B220-PerCP/Cy5.5 (clone RA3-6B2, 103236, BioLegend), CD127-PerCP/Cy5.5 (clone SB/199, 121113, BioLegend), CCR2-PE (FAB5538P, R&D Systems), CD64-APC (clone X54-5/7.1, 139306, BioLegend), Streptavidin-PerCP/Cy5.5 (405214, Biolegend) antibodies were used.<br>For immunohistochemistry, anti-PLIN1 antibody (9349S, Cell signaling) and horseradish peroxidase (HRP)-conjugated anti-rabbit IgG (414341, Nichirei Bioscience) were used.<br>For immunofluoresence study, anti-PLIN1 antibody (9349S, Cell signaling) and anti-rabbit Alexa Fluor Plus555 (A32732, Thermo fisher) were used.
Validation	All antibodies had validation statement provided on the website of the manufacturer.

Eukaryotic cell lines

Policy information about cell lines and Sex and Gender in Research

Cell line source(s)	No established or newly generated cell lines were used as experimental models in this study. Primary mesenchymal stromal cells (MSCs) were isolated from mouse bone marrow as described in the Methods.<br>293F-derived Viral Production Cells 2.0 were used solely for adeno-associated virus (AAV) production.
Authentication	293F-derived Viral Production Cells 2.0 were purchased from Thermo Fisher Scientific and were not further authenticated.
Mycoplasma contamination	Mycoplasma contamination testing was not performed.
Commonly misidentified lines (See ICLAC register)	No commonly misidentified cell lines were used in this study.

Animals and other research organisms

Policy information about studies involving animals; ARRIVE guidelines recommended for reporting animal research, and Sex and Gender in Research

Laboratory animals	C57BL/6J mice were purchased from CLEA Japan, Inc. (Tokyo, Japan). B6.129-Leprtm3(cre)Mgmj/J (#032457) and B6.Cg-Gt(ROSA) 26Sortm14(CAG-tdTomato)Hze/J (#007914) were from The Jackson Laboratory (USA). C57BL/6 mice congenic for the CD45 locus

	(CD45.1 mice) were purchased from Sankyo Labo Service Co (Tsukuba, Japan). Male mice aged 8–12 weeks and female mice aged 11–15 weeks were used in the experiments, as indicated. All mice were housed under specific pathogen-free conditions at the University of Tokyo animal facility, with controlled temperature and a 12-hour light/dark cycle. Mice had ad libitum access to standard chow and water.
Wild animals	This study did not involve wild animals.
Reporting on sex	Both male and female mice were used in experiments assessing the effects of TAC on MSC phenotypes, and sex was reported for all relevant experiments in the main text and figure legends.For MSC transplantation experiments evaluating hematopoietic and cardiac phenotypes, only male mice were used. This approach was taken to avoid potential confounding effects arising from sex mismatch between donor and recipient cells, and to ensure consistency in TAC severity and donor cell engraftment. While Hbegf downregulation in MSCs was observed in both sexes, functional analyses of MSC-mediated effects on hematopoiesis and cardiac remodeling were performed only in male mice. Therefore, whether these effects are sex-independent remains to be determined.
Field-collected samples	This study did not involve field-collected samples.
Ethics oversight	All animal experiments were approved by the University of Tokyo Ethics Committee for Animal Experiments and conducted in accordance with institutional guidelines and relevant regulations.

Note that full information on the approval of the study protocol must also be provided in the manuscript.

Clinical data

Policy information about clinical studies
All manuscripts should comply with the ICMJE guidelines for publication of clinical research and a completed CONSORT checklist must be included with all submissions.

Clinical trial registration	This study was a retrospective observational study and was not registered as a clinical trial. A total of 257 patients who underwent coronary computed tomography between January 2024 and August 2024 were included. Among these, 15 patients with a diagnosis of heart failure were identified based on electronic medical records. Age- and sex-matched healthy controls were included at a ratio of 2:1. The study was approved by the Research Ethics Committee of the Graduate School of Medicine, the University of Tokyo (reference no. 2650-14).
Study protocol	Axial slices at the level of Th7 were used to assess bone marrow fat content. The proportion of patients with fat deposition was compared between patients with heart failure and healthy controls.
Data collection	Fat tissue in vertebral bone marrow was defined as regions with CT attenuation values below 0 Hounsfield units. Data acquisition and quantification were performed using Intuition software (version 4.10, TeraRecon, Inc.).
Outcomes	The primary outcome of the study was the presence of fat deposition in vertebral bone marrow at the Th7 level, as assessed by computed tomography. The proportion of individuals with bone marrow fat deposition was compared between patients with heart failure and age- and sex-matched healthy controls.

Plants

Seed stocks	Not applicable
Novel plant genotypes	Not applicable
Authentication	Not applicable

Flow Cytometry

Plots

Confirm that:

- [x] The axis labels state the marker and fluorochrome used (e.g. CD4-FITC).
- [x] The axis scales are clearly visible. Include numbers along axes only for bottom left plot of group (a 'group' is an analysis of identical markers).
- [x] All plots are contour plots with outliers or pseudocolor plots.
- [x] A numerical value for number of cells or percentage (with statistics) is provided.

Methodology

Sample preparation	For preparation of cardiac single-cell suspensions, mice were anesthetized and intravenously injected with biotin-conjugated

anti-CD45 antibody (2 mg) prior to tissue collection. Hearts were perfused with 10 mL PBS via the left ventricle, excised, and the atria and atrioventricular valves were removed. Ventricular tissues were minced and enzymatically digested in DMEM containing collagenase I (450 U/mL), hyaluronidase (60 U/mL), and DNase I (60 U/mL) for 45 min at 37 °C with gentle agitation. The resulting cell suspensions were filtered through a 40-µm strainer, washed with HBSS supplemented with 2% FBS and 0.2% BSA, and resuspended in FACS buffer.

For bone marrow samples, tibiae, femurs, and pelvis were collected and processed for isolation of hematopoietic stem and progenitor cells (HSPCs) and mesenchymal stromal cells (MSCs).

For HSPC isolation, bone marrow cells were obtained by mechanical dissociation in MACS buffer, passed through a 23-gauge needle, and filtered through a 70-µm strainer. Red blood cells were lysed using PharmLyse solution (BD Biosciences). Cells were enriched for c-Kit+ populations using CD117 MicroBeads (Miltenyi Biotec) and autoMACS separation, followed by staining with fluorochrome-conjugated antibodies.

For MSC isolation, bone marrow was flushed and enzymatically digested with collagenase type II (1 mg/mL) and DNase I (25 U/mL) for 30 min at 37 °C. Residual bone fragments were further digested with collagenase type I (2.5 mg/mL) and DNase I (25 U/mL) for 45 min. Cells were filtered through a 100-µm strainer, subjected to red blood cell lysis, and depleted of hematopoietic and endothelial cells using CD45, CD31, and lineage depletion kits (Miltenyi Biotec) via autoMACS. Cells were washed and resuspended in FACS buffer prior to antibody staining.

Instrument

Flow cytometric analysis and cell sorting were performed using a FACSAria Fusion cell sorter (BD Biosciences).

Software

Data acquisition and instrument control were conducted using FACSDiva software (ver 9.4, BD Biosciences). Flow cytometry data were analyzed using FlowJo software (version 10.10, BD Biosciences).

Cell population abundance

For scRNA-seq of MSCs, $Lepr^+$ cells were enriched by fluorescence-activated cell sorting (FACS) using a sequential sorting strategy (yield mode followed by purity mode) to improve sorting purity. In other assays, cells were isolated based on established surface marker definitions using high-purity sorting mode. The quality of sorted populations was further assessed through downstream single-cell RNA sequencing analyses, which confirmed expected marker gene expression patterns for the respective cell populations. Any low-quality or contaminating cells were excluded during standard quality control filtering steps.

Gating strategy

Initial gating was performed to exclude debris and dead cells. Forward scatter (FSC) and side scatter (SSC) parameters were used to identify the main cell population, followed by exclusion of dead cells using DAPI staining (BD Biosciences, 564907). Doublets were excluded based on FSC-A/FSC-H gating.

Hematopoietic populations and MSCs were defined using established marker combinations.

LSK cells were defined as $CD45.2^+$ $lineage^-$ (CD4, CD8, CD11b, Ly6G, B220, CD127, Ter119) $Sca1^+$ $cKit^+$.

HSCs were defined as $CD45.2^+$ $lineage^-$ $Sca1^+$ $cKit^+$ $CD34^-$ $CD135^-$ $CD150^+$ $CD48^-$.

Multipotent progenitor (MPP) subsets were defined as follows:

MPP1: $CD34^+$ $CD135^-$ $CD150^+$ $CD48^-$

MPP2: $CD34^+$ $CD135^-$ $CD150^+$ $CD48^+$

MPP3: $CD34^+$ $CD135^-$ $CD150^-$ $CD48^+$

MPP4: $CD34^+$ $CD135^+$ $CD150^-$ $CD48^-$ (all within $CD45.2^+$ $lineage^-$ $Sca1^+$ $cKit^+$ cells).

Granulocyte-Macrophage Progenitors (GMPs) were defined as $CD45.2^+$ $lineage^-$ $Sca1^-$ $cKit^+$ $CD34^+$ $CD16/32^+$.

MSCs were defined as $LepR^+$ $lineage^-$ cells ($Ter119^-$, $CD5^-$, $CD45^-$, $Gr1^-$, $CD45R^-$, $CD71^-$, $CD11b^-$, $CD144^-$, $CD31^-$, $CD41^-$, $CD61^-$).

Cardiac macrophages were defined as $CD45.2^+$ $CD11b^+$ $CD64^+$ $Ly6G^-$ Ly6C^lo CCR2^hi or CCR2^lo.

Splenic monocytes were defined as $CD45.2^+$ $CD11b^+$ F4/80^mid $CD115^+$ Ly6C^hi.

Representative gating strategies are shown in Supplementary Figures 2b, 4a, 5, and 11.

☒ Tick this box to confirm that a figure exemplifying the gating strategy is provided in the Supplementary Information.
