# Supplementary material for: Altered bone marrow niche forms central innate immune memory driving cardiac dysfunction
Source: Nat Commun. 2026 Aug 26;17:8261. doi: 10.1038/s41467-026-76178-z (PMC13518785; doi:10.1038/s41467-026-76178-z)
Supplement: Supplementary file 3 [file 41467_2026_76178_MOESM3_ESM.pdf]

nature portfolio

Peer Review File

Altered Bone Marrow Niche Forms Central Innate Immune Memory Driving Cardiac Dysfunction

Corresponding Author: Professor Katsuhito Fujiu

This file contains all reviewer reports in order by version, followed by all author rebuttals in order by version.

Version 0:

Reviewer comments:

Reviewer #1

(Remarks to the Author)
In this study Goto and co-Authors provide novel insights on the role of BM-derived Lepr+ MSC in modulating HSPC activation and differentiation into a pro-inflammatory macrophage phenotype following myocardial injury. Of note, Authors addressed their working hypothesis by performing elegant co-transplantation experiments of BM-MSC primed by pressure overload-derived cardiac dysfunction together with healthy HSPCs and further assessing HSPC skewing towards pathological myelopoiesis.
The detrimental priming by heart failure condition was functionally linked to a decreased expression of HBEGF into MSC, thus suggesting a new candidate therapeutic target for the modulation of inflammation in heart failure.
While this work addresses a very interesting topic by means of strong methods and convincing data, I would suggest the Authors to consider whether the extracellular vesicles (EVs) released by TAC-primed BM-derived Lepr+ MSC subpopulations may have a role in reprogramming HSPC behaviour. This could be achieved by either using Lepr+ MSC-EVs, as separated and concentrated by TAC-primed BM-derived Lepr+ MSC-conditioned medium, on HSPC in vitro or by using using Lepr+ MSC-EVs in the co-transplantation experiment.
Lastly, I would suggest Authors to speculate more in details whether the mechanism of action they have identified in BM-derived Lepr+ MSC modulation of trained immunity may be also expected in heart failure consitions arising from ischemic injury, such as for acute myocardial infarct.

Reviewer #2

(Remarks to the Author)
The current study demonstrates that in heart failure induced by transverse aortic constriction (TAC), bone marrow stromal cells lose expression of HBEGF, which activates a trained immunity phenotype in hematopoietic stem and progenitor cells (HSPCs). This activation promotes pathogenic myeloid skewing and leads to the accumulation of $CCR2^+$ macrophages in the heart, contributing to cardiac fibrosis and dysfunction. Remarkably, co-transplantation of $Lepr^+$ bone marrow stromal cells from TAC mice alongside healthy HSPCs was sufficient to reprogram hematopoiesis and induce cardiac pathology in otherwise healthy recipients. Transcriptomic analysis revealed that HBEGF expression is downregulated in bone marrow stromal cells following TAC, suggesting that the absence of HBEGF may drive the maladaptive hematopoietic response. Supporting this, shRNA-mediated knockdown of HBEGF in TAC mice led to worsened cardiac fibrosis and dysfunction. While the study offers a conceptually novel mechanism of stromal cell signaling to HSPCs driving cardiac pathology, the findings are still preliminary and require further validation to support the authors' conclusions. The authors should consider:
1. Inadequate Characterization of TAC/Sham BM MSC Donor Mice:
The manuscript does not provide functional evidence confirming that the donor mice were indeed experiencing heart failure. Additionally, it lacks data demonstrating that HSPCs derived from these donors exhibit characteristics of trained immunity.
2. Insufficient Characterization of Recipient Mice Co-Transplanted with MSCs and HSPCs:
The current data do not convincingly demonstrate successful engraftment of MSCs into the bone marrow niche. The provided images fail to clearly show MSC integration, and the extent of chimerism among $Lepr^+$ bone marrow MSCs remains unaddressed. Furthermore, the conclusion that co-transplantation of MSCs with HSPCs induces cardiac dysfunction is not sufficiently supported. Additional echocardiographic parameters should be included, along with a comprehensive histological analysis of heart tissues.
3. Lack of Evidence for Equal HSPC Homing and Hematopoietic Recovery:
The manuscript does not demonstrate comparable acute homing of HSPCs to the bone marrow or equivalent hematopoietic

recovery across experimental groups. It is plausible that co-transplantation of MSCs from heart failure (HF) mice may have impaired HSPC homing or engraftment, potentially contributing to the observed immunosenescence phenotype six months post-transplantation.

4. Unvalidated AAV-FLEX Intervention:
The study lacks validation of the specificity and efficiency of the AAV-FLEX-mediated knockdown.

5. Hypothesis Testing Requires Mechanistic Validation:
The proposed hypothesis—that heart failure leads to reduced HBEGF expression, which contributes to ventricular dysfunction through trained immunity and heightened inflammation—requires further support. Overexpression of BM MSC-derived HBEGF in TAC mice, combined with detailed mechanistic analyses of immune cell phenotypes, functional assessments, and heart failure indices, would help substantiate the proposed model.

Reviewer #3

(Remarks to the Author)
In the present study, Goto K et al. show that BM Lepr+ mesenchymal stromal cells (MSCs) play a critical role in how HSPCs contribute to heart damage in heart failure (HF). When they co-transplanted Lepr+ MSCs from HF mice with healthy HSPCs, it led to the build-up of pro-inflammatory macrophages in the heart and worsened heart remodeling. This suggests that MSCs push HSPCs to differentiate into cells that promote disease. Further investigation showed that HF reduces heparin-binding EGF-like growth factor (HBEGF) in MSCs by decreasing a specific HBEGF-producing MSC group. This, in turn, suppresses EGF signaling in HSPCs. Knocking down Hbegf in MSCs specifically worsened heart function after pressure overload. HF also activated fat-producing pathways in MSCs, which aligned with the loss of Hbegf+ MSCs. Importantly, a similar HBEGF+ MSC subpopulation exists in human bone marrow, and HF in humans was linked to increased BM fat.

This is an interesting study showing that the bone marrow niche is a crucial controller of trained immunity.

1) Figure 1. The authors state that "Numerous tdTomato+ cells were observed in the BM (Fig. 1b), indicating successful engraftment of tdTomato+ MSCs into the BM." Do the authors have an idea of the efficiency of their engraftment? While numerous cells were observed, this doesn't quantify the efficiency.
Why did the authors choose to perform their analysis six months after transplantation? This timeframe needs justification.
The analysis of cardiac function could be complemented by evaluating ejection volume and other relevant parameters.
The authors should also measure the lung wet weight-to-dry weight ratio, which is a true index of heart failure.
Cardiac fibrosis should also be evaluated at the protein level, for example, by Sirius Red staining of histological sections.It would be interesting to include the gating strategy for the analysis of CCR2+ and CCR2- macrophages.

2) Figure 2: The approach is elegant, but can the authors specify why they chose four weeks as the analysis time? A central question is the identification of factors that, post-TAC, lead to the specific suppression of HBEGF signaling from MSCs. While this might be outside the scope of the current paper, it's an interesting point for future consideration.

3) Figure 3: Again, it's important to better characterize cardiac function and demonstrate if the heart is failing by measuring, for example, lung weight (refer to comments for Figure 1). Have the authors evaluated the levels of CCR2+ and CCR2- macrophages here?

4) Figure 4: The authors state that "The results summarized so far suggest that changes in MSC populations, including reduced numbers of Hbegf+ MSCs, modulate HSCs after TAC." If I'm not mistaken, where are these results presented? The connection between changes in MSC populations and HSC modulation needs clearer presentation.

5) The authors could better characterize the impact of heart failure (HF) on the phenotype of MSCs in vitro, including survival, proliferation potential, and differentiation potential. They could particularly measure the differentiation potential into fibroblasts. What is the impact of HBEGF inhibition on Lepr+ MSCs? The authors could isolate Lepr+ MSCs and at least in vitro, evaluate the effects of HBEGF invalidation on the cellular phenotype of these cells (survival, proliferation, differentiation potential, etc.). In the experimental readouts presented in Figure 4, the authors should add an experimental group with HBEGF inhibition.

6) Since the authors reveal a medullary effect, it would be important to evaluate the effect on other cardiac inflammatory cells besides macrophages, and to analyze the impact on other immune reservoirs like the spleen.

Reviewer #4

(Remarks to the Author)
Goto and Nakayama et al. describe in this manuscript how heart failure reshapes the bone marrow niche (particularly MSCs) which in turn alters HSPCs and their progeny to promote cardiac dysfunction. They link their findings mechanistically to HBEGF producing MSC, which alter HSPCs into a myeloid-bias leading to excess of CCR2+ and heart failure.

The authors use sophisticated murine models and selected human data to study their findings and the manuscript draws conclusion which are relevant for the field. The manuscript is timely and novel by connecting heart failure and immune memory. To exploit the full potential of the manuscript, I recommend a major revision and provide the following suggestions as orientation:

Major comments:
- Figure 2 and 3: It would be more elegant if the authors could perform these experiments in an unbiased scRNAseq readout of the entire bone marrow niche (thereby not preselecting specific niche cells). There are established protocols (digestion of entire bone marrow niche and FACS sorting of alive cells) for this readout - for example by the Scadden group (DOI: 10.1016/j.cell.2019.04.040). This approach would allow investigation of all niche cells (e.g. endothelial cells, fibroblasts, pericytes etc) and niche factors.
- Figure 3: Please perform a full readout including i. FACS of bone marrow (HSC, LSK, GMP, MDP), blood (WBC, Neutrophils, Monocytes) and heart (CCR2-/+ macrophages (resident vs recruited), monocytes, neutrophils, endothelial cells, fibroblasts), ii. HSPC numbers in blood (CFU assay) to assess increased mobilization of HSPC into circulation iii. add further echo data (stroke volume, ESV/EDV, cardiac mass).
- Please discuss in more detail how the authors and literature believe the bone marrow niche and LSK/HSC 'sense' heart failure. The authors should discuss the different types of heart failure (diastolic vs systolic, HFpEF vs HFrEF) and how their findings might be different depending on the type of heart failure. Describe the limitations of the TAC model. Discuss the interaction between the bone marrow marrow and heart - what signals the niche to increase myelopoesis?
- Figure 5: The manuscript would be strengthened by more human data. Could the authors collect human bone marrow cells (e.g. from sternum during sternotomy), blood collection for isolation of HSPC and/or heart tissues (e.g. LV or LAA during LVAD implantation) to verify their key findings in control (sometimes hard to find) vs. HF patients? This is a suggestion to increased the validity of the manuscript but should not be a barrier to publication.

Minor comments:
- Figure 1C: The EF of the C-MSC seems high (Fig 3 E values seem more usual for the field). Usually 'normal' EF should be around 50-80% in humans and similar in mice. Also the difference between both groups seems very minor. Please add in the figure or supplement other heart failure relevant parameters such as diastolic/systolic volumes, stroke volume and cardiac output (these can be obtained from the echo data and they authors do not have to do new experiments)
- Figure 1E-F, 2B: Please specify if this is FACS or histology data? Please show representative dot plots (in main figure) and entire gating strategy (in supplement). If possible, its nice to confirm FACS data with selected histology data (only for cardiac macrophage quantification)
- Figure 1E: If possible, please show numbers of cardiac neutrophils, endothelial cells and fibroblasts.
- Figure 1F and 3B: Does Hbegf affect HSPC proliferation or how do the authors explain the increased HSPC numbers? Have the authors accessed proliferation of HSPC in their readouts? This could be done with FACS using a BrdU dye.
- Figure 3B: Please show absolute numbers and representative dot plots.
- In general, its better to show dot plots (not heat map) for all FACS gating strategy
- If possible, include cardiovascular parameter of TAC model

Version 1:

Reviewer comments:

Reviewer #1

(Remarks to the Author)
I believe Authors addressed the Reviewers' comments properly and in a satisfactory way.

Yet, I will suggest them to expand more their discussion on the putative contribution of extracellular vesicles in the biological effects they observed in terms on intercellular communication. While I appreciate that from the methodological point of view it may be challenging to address the initial comment about suggesting to investigate the effect of Lepr+ MSC-EVs in their model, I believe the discussion on this important aspect can be further expanded (line 445-454 of the manuscript).

Reviewer #2

(Remarks to the Author)
The authors have adequately addressed the reviewers’ comments.

Reviewer #3

(Remarks to the Author)
No more comments

Reviewer #4

(Remarks to the Author)
Goto and colleagues have undertaken a substantial revision that has significantly improved the manuscript. Although not all points raised during the initial review have been addressed experimentally, the authors have responded to my concerns satisfactorily. The expanded discussion adds depth and more effectively situates the findings within the relevant context and existing literature.

My remaining suggestion is to further refine the main figures to enhance their clarity and visual appeal. I offer the following

specific recommendations:
- Several supplemental figures contain material that would strengthen the main figures if incorporated appropriately. For example, an abbreviated version of Supplemental Figure 21 could be integrated into Figure 5.
- Consider adding more schematic illustrations, similar to those in Figures 1A and 3B, to other figures. This would facilitate comprehension without requiring frequent reference to the figure legends or main text.
- Reduce unnecessary white space across all figures, particularly in Figures 4 and 5.

In summary, I commend the authors on a well-executed study and recommend the manuscript for publication once these minor revisions have been addressed.

Open Access This Peer Review File is licensed under a Creative Commons Attribution 4.0 International License, which permits use, sharing, adaptation, distribution and reproduction in any medium or format, as long as you give appropriate credit to the original author(s) and the source, provide a link to the Creative Commons license, and indicate if changes were made.
In cases where reviewers are anonymous, credit should be given to 'Anonymous Referee' and the source.
The images or other third party material in this Peer Review File are included in the article’s Creative Commons license, unless indicated otherwise in a credit line to the material. If material is not included in the article’s Creative Commons license and your intended use is not permitted by statutory regulation or exceeds the permitted use, you will need to obtain permission directly from the copyright holder.
To view a copy of this license, visit https://creativecommons.org/licenses/by/4.0/

RESPONSE TO REVIEWERS' COMMENTS

Reviewer #1 (Remarks to the Author):

In this study Goto and co-Authors provide novel insights on the role of BM-derived Lepr+ MSC in modulating HSPC activation and differentiation into a pro-inflammatory macrophage phenotype following myocardial injury. Of note, Authors addressed their working hypothesis by performing elegant co-transplantation experiments of BM-MSC primed by pressure overload-derived cardiac dysfunction together with healthy HSPCs and further assessing HSPC skewing towards pathological myelopoiesis.
The detrimental priming by heart failure condition was functionally linked to a decreased expression of HBEGF into MSC, thus suggesting a new candidate therapeutic target for the modulation of inflammation in heart failure.
While this work addresses a very interesting topic by means of strong methods and convincing data, I would suggest the Authors to consider whether the extracellular vesicles (EVs) released by TAC-primed BM-derived Lepr+ MSC subpopulations may have a role in reprogramming HSPC behaviour. This could be achieved by either using Lepr+ MSC-EVs, as separated and concentrated by TAC-primed BM-derived Lepr+ MSC-conditioned medium, on HSPC in vitro or by using using Lepr+ MSC-EVs in the co-transplantation experiment.
Lastly, I would suggest Authors to speculate more in details whether the mechanism of action they have identified in BM-derived Lepr+ MSC modulation of trained immunity may be also expected in heart failure conditions arising from ischemic injury, such as for acute myocardial infarct.

We sincerely appreciate the reviewer's insightful comment regarding the possible involvement of EVs. Previous studies have shown that HBEGF can be transferred via EVs[1] and that HBEGF-loaded nanovesicles activate EGFR signaling[2]. As the reviewer suggested, we first attempted to test the effects of EVs. However, obtaining sufficient numbers of *Lepr*$^{+}$ MSCs from post-TAC mice proved technically challenging. We could isolate only approximately 10,000 viable MSCs per mouse, which was insufficient for reliable conditioned medium preparation or EV isolation. To the best of our knowledge, previous studies characterizing MSC-derived EVs have used *in vitro* expanded MSCs from bone marrow or adipose tissue[3]. It is likely that effects of TAC on MSCs are diminished or lost during culture. We have therefore added discussion on the signaling mechanism of HBEGF, in which we address the possible involvement of EVs (p. 21). We thank the reviewer again for this valuable comment.

We greatly appreciate the reviewer's suggestion to discuss whether our findings on *Lepr*$^{+}$

MSCs and trained immunity may also apply to heart failure caused by ischemic conditions, such as myocardial infarction (MI). To the best of our knowledge, no reports or datasets specifically examining MSCs after MI have been published. However, several studies suggest similarities in HSC responses to TAC and MI. In our previous study[4], we showed that HSPC proliferation was progressively activated at 1, 2, and 4 weeks after TAC (Fig. S17). Similarly, Hoffmann *et al*. demonstrated that LT-HSCs gradually increased until day 28 after MI in mice that developed HF[5]. They also reported increased myeloid-biased progenitors after MI, consistent with our finding that reduced HBEGF signaling promotes myeloid-biased hematopoiesis. More recently, Rettkowski *et al*. reported scRNA-seq analysis of HSPCs from patients with a history of MI undergoing coronary artery bypass graft surgery[6], showing upregulation of myeloid-related genes in HSPCs. Our reanalysis of their published dataset showed that EGFR and TGF-β signaling were downregulated in HSCs from MI patients (new Suppl Fig. S20c), suggesting that alterations in these pathways may contribute to HSC responses to MI in humans.

While these findings suggest mechanistic similarities in BM responses to TAC and MI, it is important to note that acute BM responses to MI differ from our findings at subacute to chronic phase after TAC. Acute MI triggers a rapid and transient increase in bone marrow sympathetic activity and marked reduction of retention factors such as CXCL12 and SCF, resulting in prompt HSPC mobilization [7]. In contrast, we found no changes in CXCL12 or SCF at 4 weeks after TAC[4] and observed sustained impairment of BM sympathetic nerves at 1 and 4 weeks after TAC[4]. Of note, Hoffmann *et al*. showed that in their MI-induced HF model, continuous administration of a β2 adrenergic receptor blocker did not alter numbers of BM endothelial cells or HSCs 4 weeks after MI[5], supporting the notion that impairment, rather than activation, of sympathetic nerves contributes to chronic BM responses in HF. Because we have not analyzed acute BM responses to TAC, and chronic changes in BM after MI remain poorly characterized, the extent of similarity between BM responses to TAC and MI requires further investigation. We have added discussion emphasizing the need to compare how hematopoietic lineage and niche cells respond to different cardiac insults across different time points, and how innate immune memory is shaped in various HF etiologies (p. 20).

Reviewer #2 (Remarks to the Author):

The current study demonstrates that in heart failure induced by transverse aortic constriction (TAC), bone marrow stromal cells lose expression of HBEGF, which activates a trained immunity phenotype in hematopoietic stem and progenitor cells (HSPCs). This activation promotes pathogenic myeloid skewing and leads to the accumulation of CCR2$^+$ macrophages in the heart, contributing to cardiac fibrosis and dysfunction. Remarkably, co-transplantation of Lepr$^+$ bone marrow stromal cells from TAC mice alongside healthy HSPCs was sufficient to reprogram hematopoiesis and induce cardiac pathology in otherwise healthy recipients. Transcriptomic analysis revealed that HBEGF expression is downregulated in bone marrow stromal cells following TAC, suggesting that the absence of HBEGF may drive the maladaptive hematopoietic response. Supporting this, shRNA-mediated knockdown of HBEGF in TAC mice led to worsened cardiac fibrosis and dysfunction. While the study offers a conceptually novel mechanism of stromal cell signaling to HSPCs driving cardiac pathology, the findings are still preliminary and require further validation to support the authors' conclusions. The authors should consider:

1. Inadequate Characterization of TAC/Sham BM MSC Donor Mice:

The manuscript does not provide functional evidence confirming that the donor mice were indeed experiencing heart failure. Additionally, it lacks data demonstrating that HSPCs derived from these donors exhibit characteristics of trained immunity.

We appreciate the reviewer's constructive comment regarding the characterization of heart failure. We have added data demonstrating cardiac dysfunction in the post-TAC donor mice, including echocardiographic findings showing left ventricular dilation and systolic dysfunction (Fig. S1a) and elevated BNP levels (Fig. S1b). Note that because we performed echo on awake mice, control mice exhibited high ejection fraction (approximately 90%)[8]. Compared to control mice, post-TAC mice had significantly reduced ejection fractions. We found evaluation of conscious mice gave consistent and reproducible echo results as previously described[9].

Regarding trained immunity in HSPCs, we previously showed that HSPCs from the same TAC model exhibit maladaptive trained immunity[4]. In that study, transplantation of HSPCs from post-TAC mice induced cardiac dysfunction in recipients. We showed that altered differentiation potential in HSPCs shifts the balance in cardiac tissue macrophage subsets, leading to pathological cardiac remodeling. To better illustrate the rationale of the present study, we have also revised the first paragraph of Results (p.7).

2. Insufficient Characterization of Recipient Mice Co-Transplanted with MSCs and HSPCs:

The current data do not convincingly demonstrate successful engraftment of MSCs into the bone marrow niche. The provided images fail to clearly show MSC integration, and the extent of chimerism among Lepr$^+$ bone marrow MSCs remains unaddressed. Furthermore, the conclusion that co-transplantation of MSCs with HSPCs induces cardiac dysfunction is not sufficiently supported. Additional echocardiographic parameters should be included, along with a comprehensive histological analysis of heart tissues.

We appreciate the reviewer's insightful comments. As suggested, we have added quantitative assessment of MSC engraftment efficiency in recipient bone marrow by flow cytometry (Supplementary Fig. 2b). Approximately 90% of LepR$^+$ MSCs in recipients were tdTomato$^+$, indicating robust engraftment of donor MSCs from LepR-Cre; Rosa26-tdTomato mice. Fluorescence images of bone marrow sections also showed many tdTomato$^+$ cells, further supporting successful engraftment (Fig. 1b and Supplementary Fig. 2c).

We have also added echocardiography data and representative histological images (Supplementary Fig. 3a). Data were obtained from independent experiments separate from those shown in the original figure. Compared with recipients of control MSCs, recipients of TAC MSCs exhibited increased left ventricular dilation and reduced ejection fraction. Histologically, interstitial fibrosis was more pronounced in mice that received TAC MSCs as shown in Fig. 1e and Supplementary Fig. 3d. These results demonstrate that transplantation of TAC MSCs promoted left ventricular remodeling and contractile dysfunction.

3. Lack of Evidence for Equal HSPC Homing and Hematopoietic Recovery:

The manuscript does not demonstrate comparable acute homing of HSPCs to the bone marrow or equivalent hematopoietic recovery across experimental groups. It is plausible that co-transplantation of MSCs from heart failure (HF) mice may have impaired HSPC homing or engraftment, potentially contributing to the observed immunosenescence phenotype six months post-transplantation.

We appreciate the reviewer's concern regarding the potential differences in HSPC homing or engraftment efficiency. To directly address this point, we assessed HSPC numbers at early time points (1 day, 1 week, and 4 weeks) after co-transplantation of control or TAC MSCs together with CD45.2$^+$ bone marrow hematopoietic cells into CD45.1 recipient mice. As shown in Supplementary Fig. 6, the numbers of CD45.2$^+$ HSCs in the bone marrow were comparable between groups at all early time points, encompassing both the homing phase (day 1) and the initial proliferative phase (week 1 and week 4). These findings indicate that early HSC lodgment, engraftment efficiency, and initial hematopoietic recovery were similar in recipients of control and TAC MSCs. These results suggest

that TAC did not alter the ability of MSCs to support HSPC engraftment, and that the divergence in HSPC populations observed at 3 months after transplantation reflected the effects of MSCs on engrafted HSPCs. We have added these findings and clarification to the revised manuscript (p.8).

4. Unvalidated AAV-FLEX Intervention:

The study lacks validation of the specificity and efficiency of the AAV-FLEX-mediated knockdown.

We thank the reviewer for pointing out this important point. We evaluated the knockdown efficiency of the sh*Hbegf* construct *in vitro*. NIH-3T3 cells stably overexpressing murine *Hbegf* and Cre recombinase were transfected with the AAV-FLEX–sh*Hbegf* transgene plasmid. In the cells, *Hbegf* mRNA levels were reduced by approximately 50%, confirming effective repression of *Hbegf* (Fig. 3a).

For co-transplantation experiments, we isolated MSCs from LepR-Cre mice infected with either AAV-FLEX–sh*Hbegf* or control AAV carrying a scrambled sequence. These MSCs were co-transplanted with the same wild-type hematopoietic cells into recipient mice. Recipients of MSCs from AAV-FLEX-sh*Hbegf*-treated mice exhibited altered hematopoietic composition, including reduced lymphoid output and relative myeloid expansion, compared with recipients of MSCs from the scramble AAV (Supplementary Fig. 13). Because these differences emerged despite identical donor hematopoietic cells and the same AAV delivery system, they are consistent with sh*Hbegf*-mediated *Hbegf* repression in MSCs. We have added these data and clarifications to the revised manuscript (p.12).

5. Hypothesis Testing Requires Mechanistic Validation:

The proposed hypothesis—that heart failure leads to reduced HBEGF expression, which contributes to ventricular dysfunction through trained immunity and heightened inflammation—requires further support. Overexpression of BM MSC-derived HBEGF in TAC mice, combined with detailed mechanistic analyses of immune cell phenotypes, functional assessments, and heart failure indices, would help substantiate the proposed model.

We appreciate the reviewer's valuable suggestions for additional analyses to corroborate our model. We agree that MSC-specific overexpression of HBEGF after TAC would further support our model. However, MSC-specific overexpression of full-length *Hbegf in vivo* presents substantial technical difficulties. In contrast to shRNA-mediated knockdown, which requires only short hairpin sequences, overexpression necessitates delivery of the full-length *Hbegf* coding sequence under control of an MSC-specific regulatory element. The size constraints of AAV vectors, particularly when combined with Cre-dependent or MSC-specific promoter systems, make this approach technically challenging.

To further test the role of HBEGF, we analyzed the effects of HBEGF on colony forming unit (CFU) assay of HSCs. HBEGF increased multilineage CFU-granulocyte, erythrocyte, monocyte/macrophage, megakaryocyte (CFU-GEMM) and reduced CFU-granulocyte, macrophage (CFU-GM) in a dose-dependent manner (Supplementary Fig. 14). These results support a direct role of HBEGF in maintaining HSC multipotency and suppressing myeloid-lineage commitment.

Reviewer #3 (Remarks to the Author):

In the present study, Goto K et al. show that BM Lepr+ mesenchymal stromal cells (MSCs) play a critical role in how HSPCs contribute to heart damage in heart failure (HF). When they co-transplanted Lepr+ MSCs from HF mice with healthy HSPCs, it led to the build-up of pro-inflammatory macrophages in the heart and worsened heart remodeling. This suggests that MSCs push HSPCs to differentiate into cells that promote disease. Further investigation showed that HF reduces heparin-binding EGF-like growth factor (HBEGF) in MSCs by decreasing a specific HBEGF-producing MSC group. This, in turn, suppresses EGF signaling in HSPCs. Knocking down Hbegf in MSCs specifically worsened heart function after pressure overload. HF also activated fat-producing pathways in MSCs, which aligned with the loss of Hbegf+ MSCs. Importantly, a similar HBEGF+ MSC subpopulation exists in human bone marrow, and HF in humans was linked to increased BM fat.

This is an interesting study showing that the bone marrow niche is a crucial controller of trained immunity.

1) Figure 1. The authors state that "Numerous tdTomato+ cells were observed in the BM (Fig. 1b), indicating successful engraftment of tdTomato+ MSCs into the BM." Do the authors have an idea of the efficiency of their engraftment? While numerous cells were observed, this doesn't quantify the efficiency.

We thank the reviewer for this important comment. As suggested, we assessed MSC engraftment efficiency in recipient bone marrow by flow cytometry (Supplementary Figure 2b). Approximately 90% of $LepR^+$ MSCs in recipients of control or TAC MSCs were $tdTomato^+$, indicating efficient and comparable engraftment across groups.

Why did the authors choose to perform their analysis six months after transplantation? This timeframe needs justification.

We appreciate the reviewer's request for clarification regarding the timing of our analyses. In our previous study, transplantation of TAC-experienced HSCs induced progressive cardiac dysfunction that became evident several months after transplantation, with clear cardiac dysfunction observed at 6 months. To maintain consistency with that established model of long-term hematopoietic reprogramming, we selected the same 6-month time point for cardiac functional assessment in the current study.

Previous studies have reported that donor-derived HSCs establish stable, long-term hematopoiesis approximately 3–4 months after bone marrow transplantation[10]. Based on this understanding, we evaluated hematopoietic and immune phenotypes at 3 months (Fig. 1f-i), when reconstitution is considered largely stabilized. In contrast, cardiac functional deterioration develops more gradually and therefore requires longer follow-up for reliable detection (Fig. 1c, e).

To exclude early engraftment differences as a confounding factor, we quantified donor HSC numbers at early time points (day 1, week 1, and week 4; Supplementary Fig. 6) and confirmed comparable initial homing and engraftment between groups. Divergence in hematopoietic and macrophage phenotypes became evident at 3 months, whereas measurable contractile dysfunction was most robustly detected at 6 months. Accordingly, the selected time points allow distinction between early transplantation effects, stabilized hematopoietic reconstitution, and long-term cardiac remodeling. We have clearly stated these points in the revised manuscript (p. 8).

The analysis of cardiac function could be complemented by evaluating ejection volume and other relevant parameters.

We appreciate the reviewer's helpful suggestions. As suggested, we have now added additional echocardiographic parameters in Supplementary Fig. 3, including cardiac output (CO), left ventricular end-diastolic and end-systolic volumes (LVEDV, LVESV), and left ventricular mass. These additional parameters collectively demonstrate cardiac hypertrophy, chamber dilation, and impaired pump function in TAC MSC–recipient mice.

The authors should also measure the lung wet weight-to-dry weight ratio, which is a true index of heart failure.

As suggested, we measured the wet-to-dry weight ratio of the lungs in TAC MSC–recipient mice. We did not observe significant differences in the ratio between the two groups (Supplementary Fig. 3c). It has been reported that C57BL/6 mice are resistant to developing pulmonary congestion despite significant LV remodeling and systolic dysfunction after TAC or myocardial infarction [11,12]. To further substantiate cardiac dysfunction in our model, we measured plasma BNP levels. BNP was significantly elevated in recipient mice receiving TAC MSCs, consistent with worsened cardiac dysfunction (Supplementary Fig. 3c). We thank the reviewer again for prompting us to clarify these findings in the revised manuscript.

Cardiac fibrosis should also be evaluated at the protein level, for example, by Sirius Red staining of histological sections. It would be interesting to include the gating strategy for the analysis of

CCR2+ and CCR2- macrophages.

We thank the reviewer for this valuable suggestion. As suggested, we have conducted quantitative morphometric assessment of interstitial fibrosis using Picrosirius red staining. As shown in Fig. 1e and Supplementary Fig. 3d, the fibrotic area was significantly increased in the hearts of TAC MSC–recipient mice, supporting the conclusion that fibrosis is exacerbated in this group.

We have also added the flow cytometry gating strategy for distinguishing $CCR2^{hi}$ and $CCR2^{lo}$ cardiac macrophages in Supplementary Fig. 4.

2) Figure 2: The approach is elegant, but can the authors specify why they chose four weeks as the analysis time? A central question is the identification of factors that, post-TAC, lead to the specific suppression of HBEGF signaling from MSCs. While this might be outside the scope of the current paper, it's an interesting point for future consideration.

We selected the 4-week time point to analyze the effects of cardiac dysfunction on bone marrow because this represents an established phase of cardiac dysfunction following the early adaptation phase in our TAC model (Supplementary Fig. 1). In our previous study[13], we showed that phenotypic and functional alterations in HSPCs, as well as bone marrow sympathetic impairment, were clearly established by 4 weeks after TAC, suggesting that remodeling of the bone marrow microenvironment was also established at this stage. We therefore reasoned that changes in bone marrow niche cells, including MSCs, in response to cardiac dysfunction would also manifest at this time point. Indeed, our present data demonstrate changes in MSCs, including a reduction in the *Hbegf*-expressing MSC subpopulation.

We fully agree with the reviewer that we need to further elucidate the mechanism controlling MSC fate after TAC. We have expanded our analysis of regulatory networks in MSCs. Our analysis identified Hes1, a key downstream transcription factor of Notch signaling, as a hub transcription factor that mediates both the KLF4 network controlling *Hbegf* and the adipogenic PPARγ network (Supplementary Fig. 19a). *Hes1* expression levels were decreased across MSC subpopulations after TAC (Supplementary Fig. 19b), and a previous study has shown that inhibition of Notch signaling promotes adipocyte differentiation[14]. Notch signaling pathways were downregulated in MSCs after TAC (Supplementary Fig. 19c). More importantly, in silico perturbation to reduce *Hes1* altered differentiation trajectories away from the $Hbegf^{+}$ population toward the adipogenic population (Supplementary Fig. 19d). These results suggest that inhibition of Notch signaling in the BM microenvironment after TAC skews differentiation of MSCs toward adipocytes. However, this model needs to be experimentally validated, and future studies will also need to elucidate how Notch signaling is suppressed in BM after TAC. We clearly stated these limitations in p. 22.

3) Figure 3: Again, it's important to better characterize cardiac function and demonstrate if the heart is failing by measuring, for example, lung weight (refer to comments for Figure 1). Have the authors evaluated the levels of CCR2+ and CCR2- macrophages here?

We appreciate the reviewer's constructive comments. As suggested, we have further characterized cardiac function and phenotype. Suppression of *Hbegf* in bone marrow MSCs impaired systolic function (Fig. 3i and Supplementary Fig. 12a) and promoted myocardial fibrosis (Fig. 3g, h), indicating enhanced myocardial remodeling. However, the lung wet-to-dry ratio did not differ between groups (Supplementary Fig. 12b), which may reflect the resistance of C57BL/6 mice to pulmonary congestion, as discussed above.

We found accumulation of $CCR2^{hi}$, but not $CCR2^{lo}$, macrophages in the left ventricle. We have now included quantitative comparison of these macrophage subsets in Fig. 3f, with corresponding clarifications in the Results and figure legends.

4) Figure 4: The authors state that "The results summarized so far suggest that changes in MSC populations, including reduced numbers of Hbegf+ MSCs, modulate HSCs after TAC." If I'm not mistaken, where are these results presented? The connection between changes in MSC populations and HSC modulation needs clearer presentation.

We greatly appreciate the reviewer's helpful comment highlighting the need for clearer presentation of the flow. Accordingly, we have revised the manuscript as follows (Page 14, Line 1):

Our results so far demonstrated that TAC specifically impaired the inferred interaction between MSCs and HSCs via HBEGF-EGFR signaling and reduced the *Hbegf*-expressing MSC population in BM. Furthermore, MSC-specific knockdown of *Hbegf* promoted myeloid-skewed differentiation in the hematopoietic lineage, similarly to that observed after TAC, and enhanced myocardial remodeling. Collectively, these findings strongly suggest that changes in MSC populations after TAC play a pivotal role in modulating HSCs.

We believe this revision clarifies the flow of logic and thank the reviewer again for this valuable suggestion.

5) The authors could better characterize the impact of heart failure (HF) on the phenotype of MSCs in vitro, including survival, proliferation potential, and differentiation potential. They could particularly measure the differentiation potential into fibroblasts. What is the impact of HBEGF inhibition on Lepr+ MSCs? The authors could isolate Lepr+ MSCs and at least in vitro, evaluate the effects of HBEGF invalidation on the cellular phenotype of these cells (survival,

proliferation, differentiation potential, etc.). In the experimental readouts presented in Figure 4, the authors should add an experimental group with HBEGF inhibition.

We thank the reviewer for this important suggestion. To determine whether HBEGF inhibition alters MSC phenotype, we performed *ex vivo* analyses using primary bone marrow MSCs transfected with siRNA targeting *Hbegf* (siHbegf). We evaluated expression of lineage-associated markers, including *Col3a1* (fibrocyte-associated), *Wif1* (osteogenic lineage), *Pparg* (adipogenic lineage), and *Ier5* (a marker enriched in the *Hbegf*$^{+}$ MSC cluster identified in our scRNA-seq analysis). As shown in Supplementary Fig. 10b, despite the efficient knockdown of *Hbegf*, expression levels of these lineage or cluster-associated markers were not altered. These results indicate that HBEGF repression does not affect MSC differentiation potential in culture. We have added these data to the revised manuscript and clarified this point in the Results section (p. 12).

Importantly, our *in vivo* and *ex vivo* data (Fig. 4) demonstrate that heart failure promotes adipogenesis of MSCs, whereas *Hbegf* inhibition alone does not recapitulate this phenotype. Together with our TF network analysis, these findings support a model in which cardiac stress–induced upstream signals (e.g., suppression of Notch signaling) drive both adipogenic priming and reduction of *Hbegf* expression in parallel, rather than HBEGF acting as a direct autocrine regulator of MSC fate.

We have added these data and clarified this interpretation in the revised manuscript.

6) Since the authors reveal a medullary effect, it would be important to evaluate the effect on other cardiac inflammatory cells besides macrophages, and to analyze the impact on other immune reservoirs like the spleen.

As suggested, we analyzed splenic Ly6C^{hi} monocytes and found no change following *Hbegf* knockdown in MSCs (Supplementary Fig. 11d). Regarding cardiac immune cells, we additionally analyzed Ly6C^{hi} monocyte/macrophages (Supplementary Fig. 11h), Ly6G^{+} neutrophils (Supplementary Fig. 11h), and CCR2^{hi} and CCR2^{lo} macrophage subsets (Fig. 3f). Among these populations, only CCR2^{hi} macrophages were significantly increased in the sh*Hbegf* group. Consistent with the co-transplantation experiments (Fig. 1f), MSC alterations primarily promote CCR2^{hi} macrophage accumulation among cardiac myeloid cells. Although alterations in bone marrow may potentially affect lymphocyte lineages, the present study focused on the myeloid lineage because myeloid-skewed hematopoiesis was the predominant response observed after TAC and because macrophages represent the major immune cell population in the heart.

Reviewer #4 (Remarks to the Author):

Goto and Nakayama et al. describe in this manuscript how heart failure reshapes the bone marrow niche (particularly MSCs) which in turn alters HSPCs and their progeny to promote cardiac dysfunction. They link their findings mechanistically to HBEGF producing MSC, which alter HSPCs into a myeloid-bias leading to excess of CCR2+ and heart failure.

The authors use sophisticated murine models and selected human data to study their findings and the manuscript draws conclusion which are relevant for the field. The manuscript is timely and novel by connecting heart failure and immune memory. To exploit the full potential of the manuscript, I recommend a major revision and provide the following suggestions as orientation:

Major comments:

- Figure 2 and 3: It would be more elegant if the authors could perform these experiments in an unbiased scRNAseq readout of the entire bone marrow niche (thereby not preselecting specific niche cells). There are established protocols (digestion of entire bone marrow niche and FACS sorting of alive cells) for this readout - for example by the Scadden group (DOI: 10.1016/j.cell.2019.04.040). This approach would allow investigation of all niche cells (e.g. endothelial cells, fibroblasts, pericytes etc) and niche factors.

We thank the reviewer for this excellent suggestion. We fully agree that an unbiased scRNA-seq analysis would provide more comprehensive information on niche cell changes. In fact, we initially attempted scRNA-seq of lineage-negative (Lin^{-}) cells enriched by magnetic bead selection to focus on niche cells. However, as often observed with this approach, the dataset was dominated by hematopoietic cells despite enrichment. Given the limited yield of non-hematopoietic cells in our initial attempt and the inherently sparse read coverage per cell in 10x-based scRNA-seq, accurate clustering of rare populations such as MSC subsets requires sufficient cell numbers, which is difficult to achieve without enrichment. We therefore decided to enrich *Lepr*+ MSCs for our scRNA-seq analyses. We agree with the reviewer that future studies will need to characterize all niche cells to provide a clearer picture of the cellular interactions among them.

- Figure 3: Please perform a full readout including i. FACS of bone marrow (HSC, LSK, GMP, MDP), blood (WBC, Neutrophils, Monocytes) and heart (CCR2-/+ macrophages (resident vs recruited), monocytes, neutrophils, endothelial cells, fibroblasts), ii. HSPC numbers in blood (CFU assay) to assess increased mobilization of HSPC into circulation iii. add further echo data (stroke volume, ESV/EDV, cardiac mass).

We sincerely thank the reviewer for the excellent suggestions. As requested, we repeated the experiment and expanded our flow cytometric analyses across multiple tissues. In bone marrow, we quantified HSCs, LSKs, and myeloid progenitors including CMPs and GMPs. GMPs were significantly increased in the AAV-sh*Hbegf* group (Fig. 3e, Supplementary Fig. 11b), supporting myeloid-skewed hematopoiesis. We acknowledge that analysis of MDPs would have been informative. However, due to technical limitations in resolving this population with sufficient reliability, we were unable to include this subset. Nonetheless, the observed expansion of GMPs and downstream myeloid populations provides consistent evidence for enhanced myelopoiesis.

In the heart, we assessed Ly6C^{hi} monocytes/macrophages, Ly6G^{+} neutrophils, and CCR2^{hi}/CCR2^{lo} macrophage subsets. Only CCR2^{hi} macrophages were significantly increased, while other populations remained unchanged (Fig. 3f, Supplementary Fig. 11h), indicating a selective accumulation of CCR2^{hi} macrophages among myeloid cells. Splenic Ly6C^{hi} monocyte numbers were also unchanged (Supplementary Fig. 11d).

Regarding HSPC mobilization, circulating CD150^{+} HSCs and peripheral blood leukocytes were reduced in the sh*Hbegf* group (Supplementary Fig. 11g). A previous study reported enhancement of HSC mobilization by systemic EGFR blockade [15]. This discrepancy may reflect that MSC-specific *Hbegf* suppression restricts its effects on neighboring cells, including HSCs, which may differ from systemic EGFR inhibition.

We also repeated echocardiography and added cardiac output, LVEDV, LVESV, and LV mass data. These parameters showed enhanced systolic dysfunction in the sh*Hbegf* group, whereas chamber dilation and hypertrophy remained modest (Supplementary Fig. 12a).

- Please discuss in more detail how the authors and literature believe the bone marrow niche and LSK/HSC 'sense' heart failure. The authors should discuss the different types of heart failure (diastolic vs systolic, HFpEF vs HFrEF) and how their findings might be different depending on the type of heart failure. Describe the limitations of the TAC model. Discuss the interaction between the bone marrow and heart - what signals the niche to increase myelopoesis?

We greatly appreciate the reviewer's insightful suggestions. We have expanded Discussion to address the points raised by the reviewer (p. 20).

Regarding the mechanism by which BM responds to cardiac stress, we previously showed that impaired sympathetic nerve activity reduces TGF-β production by Schwann cells and subsequently promotes HSPC proliferation and skewed differentiation[4]. Maryanovich et al. showed that sympathetic denervation and constitutive deletion of *Adrb3* reduced expression of *Cxcl12*, a key retention factor for HSCs, in MSCs, while β3 agonist restored it[16]. These results suggest that sympathetic neuropathy

is involved in the response of MSCs to HF.

In addition, we have expanded our analysis of transcription factor regulatory networks that control MSCs. Our analysis identified Hes1, a key downstream transcription factor of Notch signaling, which links the KLF4 network controlling Hbegf to the adipogenic PPARγ network (Supplementary Fig. 19a). Hes1 expression was reduced across MSC subpopulations after TAC (Supplementary Fig. 19b) and *in silico* perturbation showed that Hes1 knockdown altered differentiation trajectories away from Hbegf-expressing cells toward adipogenic cells (Supplementary Fig. 19d). Moreover, inhibition of Notch signaling was shown to promote adipogenesis in BM MSCs [14]. Collectively, these results suggest that reduced Notch signaling in BM skews MSC differentiation after TAC, although how Notch signaling is impaired after TAC remains to be elucidated. Previous studies have shown that β-adrenergic receptor signaling controls Notch ligand expression in several cell types [17-19], suggesting possible regulation of Notch signaling by sympathetic impairment in HF. Importantly, we previously showed by parabiosis experiments that circulating factors are not sufficient to induce hematopoietic changes in the parabiont of mice that underwent TAC [4]. Taken together, these findings strongly suggest that sympathetic nerve dysfunction serves as a key signal that communicates cardiac stress to the BM niche.

Regarding types of HF and limitations of the TAC model, TAC causes pressure overload-induced cardiac remodeling, which progressed to systolic dysfunction (HFrEF) in our experimental setting, although diastolic dysfunction preceded systolic dysfunction. The TAC model primarily recapitulates aortic stenosis-like pathophysiology with relatively rapid progression, which may not fully represent the gradual course of human HF. Furthermore, HFpEF is a heterogeneous syndrome associated with various dysregulations including metabolic disorders such as obesity and diabetes, and chronic inflammation, which may affect the BM through mechanisms distinct from our model. Accordingly, our results may not be generalizable to all forms of HF. We have addressed these points in the Discussion (p.20).

- Figure 5: The manuscript would be strengthened by more human data. Could the authors collect human bone marrow cells (e.g. from sternum during sternotomy), blood collection for isolation of HSPC and/or heart tissues (e.g. LV or LAA during LVAD implantation) to verify their key findings in control (sometimes hard to find) vs. HF patients? This is a suggestion to increased the validity of the manuscript but should not be a barrier to publication.

We fully agree with the reviewer that additional human data would further corroborate the clinical relevance of our model. In the present study, we showed human coronary CT data demonstrating vertebral marrow adipose expansion in HF patients, consistent with the murine phenotype of adipogenic MSC remodeling. We identified *HBEGF*-expressing *LEPR*$^{+}$ MSCs in a human dataset (Fig.

5a, b). Ideally, these changes in MSCs should be further validated in humans. However, obtaining human bone marrow cells or cardiac tissues would require new institutional ethical review and patient recruitment. Moreover, because of the heterogeneous nature of HF, we are likely to need to analyze a large number of subjects. We appreciate the reviewer's recognition that such studies are beyond the scope of the present revision.

While no MSC datasets that suit our analysis are available, Rettkowski et al. very recently reported an analysis of HSPCs in patients with a history of myocardial infarction[6]. We reanalyzed the dataset and found that EGFR signaling, in addition to TGF-β signaling, was downregulated in HSCs from those patients, which is consistent with our mouse HSC analysis and points to the involvement of EGFR signaling in the response to myocardial infarction (Supplementary Fig. 20c).

Minor comments:

- Figure 1C: The EF of the C-MSC seems high (Fig 3 E values seem more usual for the field). Usually 'normal' EF should be around 50-80% in humans and similar in mice. Also the difference between both groups seems very minor. Please add in the figure or supplement other heart failure relevant parameters such as diastolic/systolic volumes, stroke volume and cardiac output (these can be obtained from the echo data and they authors do not have to do new experiments)

We thank the reviewer for these important comments. We have now added additional cardiac function parameters to Supplementary Figure 3a, including LVESV / LVEDV, Cardiac output and LV mass. These indices consistently confirmed systolic dysfunction in TAC MSC–recipient mice across replicated experiments (including those shown in Figures 1 and 3).

Regarding the high EF values (80–90%), we note that EF in mice is strongly influenced by anesthetic conditions, particularly heart rate suppression under deep anesthesia. We noted that echocardiography was performed in awake mice with heart rates maintained between 650–700 beats per minute (Supplementary Fig. 3a), resulting in uniformly elevated EF values. Thus, relative differences, rather than absolute values, should be used for interpretation[8]. Importantly, the EF values observed in our study correspond to approximately 50–60% fractional shortening (FS), which is consistent with values reported in previous studies using similar experimental conditions[9].

Although EF differences appear to be modest, the multi-parameter echocardiographic evidence (SV, LV volumes, and cardiac output), several fibrotic markers, including Picrosirius Red staining, and plasma BNP levels collectively support development of cardiac remodeling in TAC MSC-recipient mice. We have clarified these points in the revised manuscript and figure legends.

- Figure 1E-F, 2B: Please specify if this is FACS or histology data? Please show representative

dot plots (in main figure) and entire gating strategy (in supplement). If possible, its nice to confirm FACS data with selected histology data (only for cardiac macrophage quantification)

We thank the reviewer for the helpful suggestions. The data presented in original Fig. 1E–F (new Fig. 1f, h) and original Fig. 3B (new Fig. 3c) in the revised manuscript) were obtained by flow cytometry. We have now added representative dot plots to the corresponding main figures in Fig. 1i and 3d, and the complete gating strategy in Supplementary Fig. 4a and 5.

In addition, as suggested, we have included representative histological images to confirm cardiac macrophage accumulation, which supports the flow cytometric findings (Supplementary Fig. 4a and 4b). We have clarified these methodological details in the revised figure legends.

- Figure 1E: If possible, please show numbers of cardiac neutrophils, endothelial cells and fibroblasts.

We thank the reviewer for this valuable suggestion. In the MSC transplantation experiment shown in Fig. 1, we prioritized histological analysis and therefore did not quantify cardiac neutrophils, endothelial cells, or fibroblasts at that specific time. We have now clarified this point in the manuscript. However, we evaluated cardiac immune cell subsets in the AAV-infected TAC model, including Ly6G$^+$ neutrophils and Ly6C$^+$ monocytes. Both populations were detected at much lower abundance relative to macrophages, and no significant differences were observed between groups (Supplementary Fig. 11h). Whether endothelial cells and fibroblasts are similarly affected remains an open question.

- Figure 1F and 3B: Does Hbegf affect HSPC proliferation or how do the authors explain the increased HSPC numbers? Have the authors accessed proliferation of HSPC in their readouts? This could be done with FACS using a BrdU dye.

As shown in original Fig. 1F (new Fig. 1h) and 3B (new Fig. 3c), TAC-MSCs and *Hbegf*-inhibited MSCs led to increased HSC numbers in the bone marrow at 3 months after the transplantation. While this phenotype is consistent with enhanced maintenance or retention of HSCs, we acknowledge that it does not directly demonstrate proliferation. Importantly, to exclude the possibility that increased HSC numbers resulted from differences in engraftment or homing, we assessed early-stage LSK numbers after MSC transplantation (Supplementary Fig. 6) and found no significant differences between groups. At the early proliferative phase following transplantation (1 week), no significant differences in EdU$^+$ HSCs were observed between groups. These results suggest that the later increase in HSC numbers reflects changes in *in vivo* regulation rather than initial engraftment efficiency. However, we did not assess EdU incorporation at later time points, because it is unknown when differences in HSC numbers

might arise. It is likely that a low-level increase in proliferation rate accumulates over a long time period, which would require longitudinal time-course analysis to reliably detect. Consequently, we now clearly state in the Discussion that the observed increase in HSC numbers may result from altered quiescence, survival, differentiation bias, or mobilization, in addition to potential effects on proliferation. We also highlight that future studies incorporating direct proliferation assays will be valuable to dissect these mechanisms. We thank the reviewer for pointing out this important point, which has been incorporated into the revised manuscript.

- Figure 3B: Please show absolute numbers and representative dot plots.

We thank the reviewer for this helpful suggestion. We have added absolute numbers of HSPCs and representative dot plots in Supplementary Fig. 11a.

- In general, its better to show dot plots (not heat map) for all FACS gating strategy

We thank the reviewer for this helpful suggestion. We have replaced heatmap-based gating displays with dot plots.

MSCs: Supplementary Fig. 2b
Cardiac macrophages: Supplementary Fig. 4a
HSCs and MPPs: Supplementary Fig. 5
CMP and GMP: Supplementary Fig. 11c
Splenic monocytes: Supplementary Fig. 11e

- If possible, include cardiovascular parameter of TAC model

We thank the reviewer for this helpful suggestion. To better characterize the TAC model, we have now added additional cardiovascular parameters of donor TAC mice, including LVEF, Left ventricular volumes (LVEDV/LVESV) and cardiac mass. These data are included in Supplementary Fig. 1, and the corresponding descriptions have been added to the revised manuscript.

References

1 Sethuraman, A. *et al.* BHLHE40 confers a pro-survival and pro-metastatic phenotype to breast cancer cells by modulating HBEGF secretion. *Breast Cancer Res* **20**, 117 (2018). https://doi.org/10.1186/s13058-018-1046-3

2 Poh, Q. H., Rai, A., Cross, J. & Greening, D. W. HB-EGF-loaded nanovesicles enhance trophectodermal spheroid attachment and invasion. *Proteomics* **24**, e2200145 (2024). https://doi.org/10.1002/pmic.202200145

3 Goloviznina, N. A. *et al.* Mesenchymal Stromal Cell-derived Extracellular Vesicles Promote Myeloid-biased Multipotent Hematopoietic Progenitor Expansion via Toll-Like Receptor Engagement. *J Biol Chem* **291**, 24607-24617 (2016). https://doi.org/10.1074/jbc.M116.745653

4 Nakayama, Y. *et al.* Heart failure promotes multimorbidity through innate immune memory. *Sci Immunol* **9**, eade3814 (2024). https://doi.org/10.1126/sciimmunol.ade3814

5 Hoffmann, J. *et al.* Post-myocardial infarction heart failure dysregulates the bone vascular niche. *Nat Commun* **12**, 3964 (2021). https://doi.org/10.1038/s41467-021-24045-4

6 Rettkowski, J. *et al.* Modulation of bone marrow haematopoietic stem cell activity as a therapeutic strategy after myocardial infarction: a preclinical study. *Nat Cell Biol* **27**, 591-604 (2025). https://doi.org/10.1038/s41556-025-01639-4

7 Dutta, P. *et al.* Myocardial infarction accelerates atherosclerosis. *Nature* **487**, 325-329 (2012). https://doi.org/10.1038/nature11260

8 Lairez, O. *et al.* Anesthetic regimen for cardiac function evaluation by echocardiography in mice: comparison between ketamine, etomidate and isoflurane versus conscious state. *Lab Anim* **47**, 284-290 (2013). https://doi.org/10.1177/0023677213496236

9 Rottman, J. N. *et al.* Temporal changes in ventricular function assessed echocardiographically in conscious and anesthetized mice. *J Am Soc Echocardiogr* **16**, 1150-1157 (2003). https://doi.org/10.1067/S0894-7317(03)00471-1

10 Cabezas-Wallscheid, N. *et al.* Identification of regulatory networks in HSCs and their immediate progeny via integrated proteome, transcriptome, and DNA methylome analysis. *Cell Stem Cell* **15**, 507-522 (2014). https://doi.org/10.1016/j.stem.2014.07.005

11 Tannu, S., Allocco, J., Yarde, M., Wong, P. & Ma, X. Experimental model of congestive heart failure induced by transverse aortic constriction in BALB/c mice. *J Pharmacol Toxicol Methods* **106**, 106935 (2020). https://doi.org/10.1016/j.vascn.2020.106935

12 Mohammed, S. F. *et al.* Variable phenotype in murine transverse aortic constriction. *Cardiovasc Pathol* **21**, 188-198 (2012). https://doi.org/10.1016/j.carpath.2011.05.002

13 Nakayama, Y. *et al.* A long noncoding RNA regulates inflammation resolution by mouse macrophages through fatty acid oxidation activation. *Proc Natl Acad Sci U S A* **117**, 14365-14375 (2020). https://doi.org/10.1073/pnas.2005924117

14 Song, B. Q. *et al.* Inhibition of Notch Signaling Promotes the Adipogenic Differentiation of Mesenchymal Stem Cells Through Autophagy Activation and PTEN-PI3K/AKT/mTOR Pathway. *Cell Physiol Biochem* **36**, 1991-2002 (2015). https://doi.org/10.1159/000430167

15 Ryan, M. A. *et al.* Pharmacological inhibition of EGFR signaling enhances G-CSF-induced hematopoietic stem cell mobilization. *Nat Med* **16**, 1141-1146 (2010). https://doi.org/10.1038/nm.2217

16 Maryanovich, M. *et al.* Adrenergic nerve degeneration in bone marrow drives aging of the hematopoietic stem cell niche. *Nat Med* **24**, 782-791 (2018). https://doi.org/10.1038/s41591-018-0030-x

17 Chen, H. *et al.* Adrenergic signaling promotes angiogenesis through endothelial cell-tumor cell crosstalk. *Endocr Relat Cancer* **21**, 783-795 (2014). https://doi.org/10.1530/ERC-14-0236

18 Ma, X., Lv, K., Wu, L. & Ouyang, T. Propranolol inhibits stemness of hemangioma through Jagged1. *Ann Transl Med* **9**, 1682 (2021). https://doi.org/10.21037/atm-21-5563

19 Wang, X. *et al.* beta-Adrenergic signaling induces Notch-mediated salivary gland progenitor cell control. *Stem Cell Reports* **16**, 2813-2824 (2021). https://doi.org/10.1016/j.stemcr.2021.09.015
